# Recent Advances in Carbon‐Based Electrodes for Energy Storage and Conversion

**DOI:** 10.1002/advs.202301045

**Published:** 2023-04-25

**Authors:** Gopalakrishnan Kothandam, Gurwinder Singh, Xinwei Guan, Jang Mee Lee, Kavitha Ramadass, Stalin Joseph, Mercy Benzigar, Ajay Karakoti, Jiabao Yi, Prashant Kumar, Ajayan Vinu

**Affiliations:** ^1^ Global Innovative Centre for Advanced Nanomaterials (GICAN) College of Engineering, Science and Environment (CESE) The University of Newcastle Callaghan NSW 2308 Australia

**Keywords:** carbon nanotubes, fullerene, graphene, hydrogen evolution reaction, Li‐ion batteries, Na‐ion batteries, supercapacitors

## Abstract

Carbon‐based nanomaterials, including graphene, fullerenes, and carbon nanotubes, are attracting significant attention as promising materials for next‐generation energy storage and conversion applications. They possess unique physicochemical properties, such as structural stability and flexibility, high porosity, and tunable physicochemical features, which render them well suited in these hot research fields. Technological advances at atomic and electronic levels are crucial for developing more efficient and durable devices. This comprehensive review provides a state‐of‐the‐art overview of these advanced carbon‐based nanomaterials for various energy storage and conversion applications, focusing on supercapacitors, lithium as well as sodium‐ion batteries, and hydrogen evolution reactions. Particular emphasis is placed on the strategies employed to enhance performance through nonmetallic elemental doping of N, B, S, and P in either individual doping or codoping, as well as structural modifications such as the creation of defect sites, edge functionalization, and inter‐layer distance manipulation, aiming to provide the general guidelines for designing these devices by the above approaches to achieve optimal performance. Furthermore, this review delves into the challenges and future prospects for the advancement of carbon‐based electrodes in energy storage and conversion.

## Graphical abstract

Carbon‐based nanomaterials, including graphene, fullerenes, and carbon nanotubes, are among the most rapidly emerging building blocks for nanotechnologies. This review elucidates the advantages and the crucial role of these family materials and summarizes the prevailing strategies for achieving high‐performance energy storage and conversion applications.

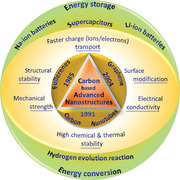

## 1 Introduction

The growing energy consumption, excessive use of fossil fuels, and the deteriorating environment have driven the need for sustainable energy solutions.^[^ ^1^ ^]^ Renewable energy sources such as solar, wind, and tidal have received significant attention, but their production cost, efficiency, and intermittent supply continue to pose challenges to widespread adoption.^[^ ^2^ ^]^ In addition, renewable energy sources such as solar and wind are limited at night or on cloudy and windless days. In light of these challenges, efficient energy storage has become crucial in the quest for sustainable energy, particularly when integrating renewable energy sources. Electrochemical energy generation (batteries) and storage (supercapacitors) technologies have witnessed exponential growth in the recent past and have proved to be promising technologies ranging from small‐scale portable electronics to vehicles.^[^ ^3^ ^]^ Despite their potential, the large‐scale commercial use of energy storage using these technologies is still unsatisfactory, though the energy storage system seems perfect to integrate with renewable energy.^[^ ^4^ ^]^

Batteries and supercapacitors are currently the primary devices for energy storage. The use of batteries has revolutionized the field of energy storage due to their high energy density which is lacking in supercapacitors.^[^ ^5^ ^]^ Supercapacitors do possess high power density and are good candidates for immediate power supply and recharging.^[^ ^6^, ^7^, ^8^ ^]^ Despite the ongoing developments in supercapacitors and batteries, continuous improvements related to their cyclic efficiency, rate performance, cost, and safety are required.^[^ ^9^, ^10^ ^]^ Energy conversion is another important criterion for harnessing renewable energy, wherein it can be transformed into chemical energy and stored in the form of hydrogen produced by the electrochemical splitting of water. When required, this energy can be utilized in devices like fuel cells.^[^ ^11^ ^]^ Therefore, new advances in energy storage and electrocatalytic hydrogen evolution reaction (HER) are of prime importance in addressing the most prevalent issues of modern‐day society, including climate change due to the greenhouse effect and the depletion of fossil fuels.^[^ ^12^, ^13^, ^14^ ^]^

Energy storage and conversion systems using supercapacitors, batteries, and HER hinge heavily on the chemistry of materials employed for electrodes and electrocatalysts.^[^ ^8^, ^15^, ^16^, ^17^, ^18^, ^19^, ^20^, ^21^ ^]^ The chemical bonds of these materials determine the capacity to store electrical energy in the form of chemical energy. The charge storage and conversion efficiency are controlled by several factors, including the electrochemical activity, conductivity, and structural stability of materials. Generally speaking, the superior the electrochemical properties of the material, the higher efficient the system is in the storage and conversion of energy. Therefore, the design and development of materials tailored to meet specific energy storage applications become a critical aspect of materials science research. As a representative example, the discovery of LiCoO_2_/graphite and LiFePO_4_ led to their commercialization for lithium‐ion batteries, which is a perfect testament to the impact that optimized material design has on energy storage performance.^[^ ^22^ ^]^ Over the years, several types of materials have been developed as electrodes for energy storage systems. However, the limitations in terms of low energy density, low power density, and/or low durability are the confronting issues that need to be addressed on an ongoing basis.^[^ ^3^, ^23^, ^24^, ^25^, ^26^, ^27^ ^]^ In particular, under high cyclability and load, batteries with the anode coating of inorganic materials catch fire. In this context, carbon‐based nanostructures have emerged as leading materials in energy storage and conversion technologies due to their electrical, mechanical, and optical properties, easily tunable morphologies, high surface area, and high thermal and chemical stabilities.^[^ ^18^, ^28^, ^29^, ^30^, ^31^ ^]^

In the last decades, three sp^2^ hybrid forms of carbon, i.e., graphene, carbon nanotubes (CNTs), and fullerenes, have been extensively investigated for energy storage and conversion applications. To begin with, the discovery of graphene has triggered the explosive growth of graphene‐based materials for applications in these hot fields. The appealing properties of graphene, including 2D layered structure, large thermal conductivity (5000–6000 W m^−1^ K^−1^), high electrical conductivity (≈10^6^ S cm^−1^), high intrinsic carrier mobility (300 000 cm^2^ V^−1^ s^−1^), high mechanical strength (Young's modulus 1.0 TPa), and a high theoretical surface area of 2630 m^2^ g^−1^, put it into the topmost category of materials for electrochemical energy storage and conversion.^[^ ^32^, ^33^ ^]^ Especially, the properties of graphene can be further improved through advanced strategies such as heteroatom doping which serves the purpose of increasing active sites through the introduction of defects in the structure.^[^ ^34^ ^]^ The introduction of porosity into graphene nanosheets to yield holey graphene (HG) and the further hybridization of HG with nanomaterials is another facile approach to unraveling its full potential for electrochemical storage purposes.^[^ ^35^ ^]^ A high surface area is required for diffusion‐controlled reactions in the energy storage system and surface manipulation allows for the enhancement of its properties.^[^ ^36^ ^]^ The second type of materials, CNTs, which can be considered as rolled graphene layers, possess the flexibility to exist as single‐walled carbon nanotubes (SWCNTs) or multi‐walled carbon nanotubes (MWCNTs) with an adjustable diameter (in the nm range) and length (in the µm range), enabling them with different shapes ranging from nano horns to nano coils.^[^ ^37^ ^]^ CNTs exhibit exciting features, including their lightweight features, high surface areas (SWCNTs, 1315 m^2^ g^−1^), ease of transformation into nanostructures with desirable properties, high electrical conductivity (10^2^–10^6^ S cm^−1^), high chemical and thermal stability, and easy production in bulk quantities.^[^ ^38^, ^39^, ^40^ ^]^ Owing to their excellent features, CNTs are also good candidates for energy storage and conversion applications.^[^ ^41^, ^42^ ^]^ The third type of carbon‐based nanostructures are fullerenes, also known as balls of carbon (buckyballs), which hold potential advantages over graphene and CNT in terms of ease of controlling their structures at the molecular level.^[^ ^43^ ^]^ Fullerenes are highly conductive materials owing to their small bandgaps that are beneficial to fast charge transfer. Their high surface area and round structure facilitate the to‐and‐fro movement of ions around the ball‐shaped structure.^[^ ^44^ ^]^ Nevertheless, fullerenes remain the least explored form of carbon among the three forms for energy storage and conversion.^[^ ^30^ ^]^

**Figure 1:**
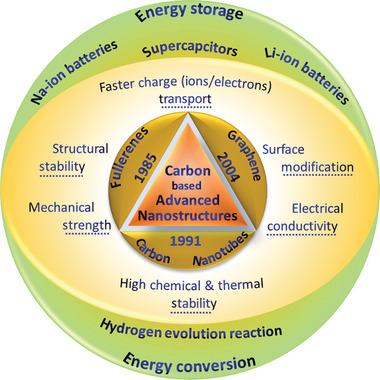
The development, properties, and applications of graphene, CNTs, and fullerenes.

In this review, we have explored the latest advancements in these three types of carbon nanostructures (graphene, CNTs, and fullerenes) for electrochemical energy storage, including supercapacitors, Li‐ion/Na‐ion batteries, and HER. The development and various properties of these three carbon forms are depicted in **Figure** **1**. With the rise in demand for renewables and with broadening the scope of usage (in electronic gadgets, displays, drones, electric vehicles, and etc.), high load and rate capability, high cyclability, and fire safety become crucial issues. Graphene and CNTs having high crystallinity, electronic mobility, and importantly high thermal conductivity, are uniquely suited for energy storage applications. It should be mentioned that although the applications of carbon nanostructures in energy storage and conversion have been reviewed on several occasions in the past few years,^[^ ^3^, ^10^, ^45^, ^46^, ^47^, ^48^, ^49^, ^50^, ^51^, ^52^, ^53^, ^54^, ^55^, ^56^, ^57^, ^58^, ^59^, ^60^, ^61^, ^62^, ^63^, ^64^, ^65^ ^]^ it is a rapidly evolving and highly active field, and the vast amount of research carried out worldwide has accumulated very quickly. Moreover, the present status of the state‐of‐the‐art design of carbon‐based pure/doped/hybrid nanomaterials, their functionalities with a better in‐depth understanding of materials, as well as their interfaces and phenomena occurring therein, can help design novel next‐generation batteries, supercapacitor or hybrid devices with new applications. Therefore, it becomes significant to review the ongoing progress in the field, including the new synthesis strategies, structure–property evaluations and application criteria which help to provide updated insights for future research. Most importantly, the new trends and concepts in the use of these three materials for energy storage via the battery and supercapacitor‐based systems and their role as electrocatalysts for HER are systematically discussed.

## 2 Carbon‐Based Nanomaterials

Carbon is one of the most important and abundant materials in the earth's crust. Carbon has several kinds of allotropes, such as graphite, diamond, fullerenes, nanotubes, and wonder material graphene, mono/few‐layered slices of graphite, which has been material of intense research in recent times.^[^ ^66^ ^]^ The physicochemical properties of these allotropes are unique and highly dependent on how the carbon atoms are arranged. It was believed that graphite and diamond were the only stable forms of carbon until the discovery of fullerenes in 1985 by Richard E. Smalley, who was awarded the Nobel Prize in 1996.^[^ ^67^ ^]^ Fullerenes are electron‐deficient materials and thus can act as an electron‐transferring media with mobility from 10^−4^ to 10^−3^ cm^2^ V^−1^ s^−1^, widely used in solar cells as electron acceptors.^[^ ^68^ ^]^ In addition, fullerenes have been extensively used for drug delivery, supercapacitors, fuel cells, hydrogen storage, and antibacterial shield on water pipelines and lubricants.^[^ ^69^, ^70^, ^71^, ^72^, ^73^, ^74^ ^]^

Later in 1991, Ijima first observed 1D CNTs containing multiple walls.^[^ ^75^ ^]^ CNTs were first observed as one part of fullerenes, called cylindrical fullerenes or buckytubes. Shortly after the observation, Ijima reported the synthesis of CNTs by an arc‐discharge evaporation method.^[^ ^76^ ^]^ Later, SWCNTs were synthesized on substrates using chemical vapor deposition (CVD) with the transition metal as catalysts on various substrates such as SiO_2_, Al_2_O_3_, and ZrO_2,_^[^ ^77^ ^]^ which significantly accelerated the research and development of CNTs. CNTs have many exciting properties and features, such as chirality dependent metallic/semiconducting nature, excellent chemical/mechanical stability, and higher thermal/electrical conductivity, enabling them for applications like transistors, sensors, optical devices, energy storage devices, bio‐applications, and so on.^[^ ^78^, ^79^, ^80^, ^81^, ^82^ ^]^

The research of carbon‐based materials has been booming after the discovery of 2D graphene exfoliated using Scottish tape by Andre Geim and Konstantin Novoselov in 2004,^[^ ^83^ ^]^ both of whom were awarded the Nobel Prize in 2010.^[^ ^66^, ^84^ ^]^ Such discovery also starts the research of various 2D materials.^[^ ^85^, ^86^, ^87^, ^88^ ^]^ Graphene is thermodynamically stable and exhibits extraordinary mechanical, thermal, electrical, and electronic properties. Under atmospheric conditions, graphene shows electron mobility in the order of 10^4^ cm^2^ V^−1^ s^−1^ with multiple potential applications in energy storage and conversion, sensors, and water‐splitting, but the zero band‐gap nature limits its application for electronics.^[^ ^89^, ^90^ ^]^ In order to introduce bandgap, graphene sheets have been doped with hetero‐atoms like nitrogen, boron, sulfur, and phosphorous to utilize them in field‐effect transistors and energy storage devices.

**Figure 2:**
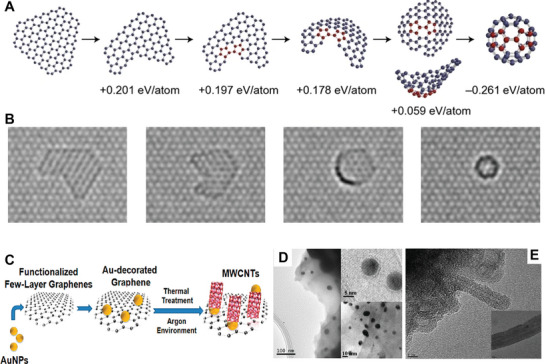
A) Quantum chemical modeling of the fullerene formation from a graphene flake. The corresponding TEM images of each structure are shown in (B). Reproduced with permission.^[^ ^92^ ^]^ Copyright 2010, Springer Nature. C) The schematic illustration of the conversion from graphene into CNT, where AuNPs as catalytic elements promote the reaction at low temperatures without additional carbon sources. TEM images of the AuNPs on graphene sheets D) before the reaction and E) after a radio frequency CVD process. Reproduced with permission.^[^ ^91^ ^]^ Copyright 2012, American Chemical Society.

In short, all the above‐discussed carbon materials exhibited great potential with unique physiochemical properties, and their underlying relationships have drawn profuse attention recently. Interestingly, these carbon allotropes can be converted into other forms under a special synthetic set of thermodynamic conditions. For example, a single graphene sheet can be twisted or rolled into fullerene balls (**Figure** **2**A,B) or nanotubes (Figure 2C–E).^[^ ^91^, ^92^ ^]^ Contrary to this, CNTs and fullerenes can be unzipped to graphene nanoribbons and graphene dots.^[^ ^93^ ^]^ Understanding these zipping/unzipping processes can help in future material design. For example, partially unzipped CNTs at the tips of a CNT forest can be realized. Even on‐the‐spot doping at opening (unzipped locations) can be implemented and employed in energy devices.

## 3 Carbon‐Based Electrodes for Supercapacitors

### 3.1 Introduction to Supercapacitors

**Figure 3:**
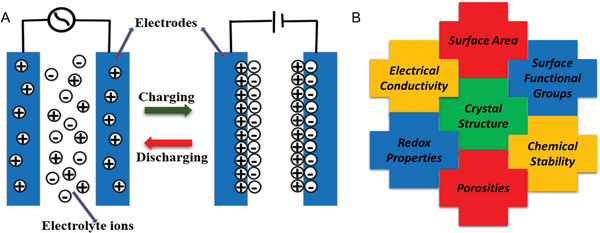
A) Working principle of an electrochemical double‐layer capacitor. B) Factors influencing the performance of a supercapacitor.

Supercapacitors store electrochemical charge by electrostatic force when a potential is applied between two parallel electrodes. It can store more energy compared to ordinary capacitors but less than batteries. Notably, supercapacitors have much higher charge/discharge stability than batteries.^[^ ^94^ ^]^ Electrochemical supercapacitors consist of two parallel electrodes separated by a dielectric membrane, allowing electrolyte ions to transport between the electrodes, as shown in **Figure** **3**A. Supercapacitive behavior can be divided into electrochemical double‐layer capacitance (EDLC) and pseudo‐capacitance. EDLC follows the principle of Helmholtz double layer, where the electrode forms an interface with the electrolyte with a few nanometer separation distances (non‐Faradaic), while pseudo‐capacitors work on charge transfer between the electrode and the electrolyte (Faradaic). Supercapacitors charge much faster than batteries and exhibit excellent lifespan as they do not suffer from volume expansion like secondary batteries even after multiple charge/discharge cycles. Owing to the better ion transportation and intercalation within the electrode materials in supercapacitors, they offer much higher power density and give a power output of more than 10 times compared to that of batteries. However, the most critical concern for supercapacitors is the relatively lower energy density compared to batteries, which solely depends on the type of electrodes and electrolytes.

For supercapacitors, electrodes are critical for achieving good performance. Though many kinds of electrodes have been investigated, carbon‐based nanomaterials are one of the best categories for supercapacitors.^[^ ^95^, ^96^, ^97^, ^98^, ^99^, ^100^ ^]^ In particular, carbon‐based materials have variable structures and excellent electrical conductivity with unique high surface‐to‐volume ratios, which are beneficial to achieving high energy density supercapacitors. Increasing high surface area is one of the most straightforward strategies for obtaining high‐performance supercapacitors. In this regard, carbon‐based materials have been activated by different activation agents like ZnCl_2_, and efforts have also been made to achieve high surface area carbon materials from biomass. However, most of the activated carbons reported in the previous literature have hidden pores with the broad pore size distribution and exhibit relatively low electrical conductivity. On the one hand, nonuniform pore diameter lowers effective surface area/volume and limits the accessibility of active sites, thus reducing the overall capacitive performance. On the other hand, lowered electrical conductivity is undesirable as it gives rise to the curtailed current collection due to enhanced electronic scattering, which could hinder the motion of electrolyte ions.

The carbon allotropes like fullerenes, CNTs, and graphene exhibit excellent EDLC behavior owing to intrinsic features such as good conductivity and relatively high surface area. In order to achieve pseudocapacitive behavior in addition to EDLC behavior for enhancing supercapacitor performance, these materials have been functionalized with oxygen functionalities or doped with hetero atoms, leading to an increase in energy density. Other techniques, such as forming composites with transition metal oxides, have also been employed to enhance pseudo‐capacitance, resulting in an increase in overall capacitance. Figure 3B lists the typical factors influencing a supercapacitor's performance.

These carbon nanomaterials are highly stable under high‐acidic high‐voltage conditions, which is impossible for those made from conventional transition metal oxides. A comparison between various carbon‐based supercapacitors and their performance is summarized in **Table** **1**.

**Table 1:** The supercapacitor behavior of different materials based on graphene, CNTs, and fullerenes, as well as their binary and ternary composites (Notes: SA—surface area, FCL700: N and Fe co doped porous carbon derived from ferrocenylpyrrolidine C60, HTFT_2000: Mesoporous graphitic carbon microtubes derived from fullerene C_70_, G/CNTs‐200: CNT@CZIF‐2: composite of nitrogen doped porous carbon derived from zeolitic imidazole framework and CNT, RGO/UCNT/PANI; Ternary composite of reduced graphene oxide, unzipped CNTs and polyaniline, Fe_2_O_3_NDs@NG—Fe_2_O_3_ nanodots supported on nitrogen doped graphene sheets, MCFC‐900: Amorphous mesoporous carbon cubes derived from mesoporous crystalline fullerene C_70_, VN/NG—A composite of vanadium nitride and nitrogen doped graphene, GO/Fc—graphene oxide and ferrocene composite produced using vapor deposition, MoS_2_/G: composite of molybdenum sulfide and graphene, 3DG/CNT—Composite of 3D graphene and CNTs, P‐MWNT‐PANI—polyaniline nanowire arrays grown on dendrimer functionalized multiwalled CNTs, CNTs@Gr‐CNF‐5—ternary composite made up of CNTs, graphene and carbon nanofibers, G/PANI/CNTs—composite of graphene, polyaniline and CNTs, mCF@CC—mesoporous C60 fullerene micro‐particles supported on carbon cloth, G—graphene prepared from graphene oxide aqueous solution using excimer laser irradiation reduction technique, PANI‐PORGO—polyaniline functionalized reduced graphene oxide, NG—nitrogen doped graphene, and OMC/G—composite of ordered mesoporous carbon and graphene)

Material	SA [m^2^ g^−1^]	Capacitance [F g^−1^]	Current density [A g^−1^]	Electrolyte	Electrode configuration	Refs.
FCL700	49.4	505.4	0.1	6 m KOH	3 electrodes	[43]
HTFT_2000	334	184.6	0.5	1 m H_2_SO_4_	3 electrodes	[101]
G/CNTs‐200	196	202	0.5	6 m KOH	3 electrodes	[102]
CNT@CZIF‐2	287	324	0.5	6 m KOH	3 electrodes	[103]
RGO/UCNTs/PANI	83.58	359.3	1.0	1 m H_2_SO_4_	3 electrodes	[104]
Fe_2_O_3_NDs@NG	–	274	1.0	2 m KOH	3 electrodes	[105]
MCFC‐900	642.6	205	1.0	1 m H_2_SO_4_	3 electrodes	[106]
Fe‐MFC_60_‐150	598	112.4	0.1	2 m KOH	3 electrodes	[107]
VN/NG	809	445	1.0	6 m KOH	3 electrodes	[108]
GO/Fc	500	178	1.0	1 m Na_2_SO_4_	3 electrodes	[109]
MoS_2_/G	–	227	0.1	1 m Na_2_SO_4_	3 electrodes	[110]
3DG/CNT	–	197.2	–	1 m Na_2_SO_4_	3 electrodes	[111]
P‐MWNT‐PANI	15.53	568	0.5	1 m H_2_SO_4_	3 electrodes	[112]
CNTs@Gr‐CNF‐5	720.8	218	1.0	6 m KOH	3 electrodes	[113]
G/PANI/CNTs	230	638	0.5	1 m H_2_SO_4_	3 electrodes	[114]
mCF@CC	–	440	2.0	6 m LiCl	3 electrodes	[115]
G	58.9	141	1.04	0.5 m Na_2_SO_4_	2 electrodes	[116]
PANI‐PORGO	–	369	1.0	1 m H_2_SO_4_	3 electrodes	[117]
NG	–	225.2	1.0	6 m KOH	3 electrodes	[118]
OMC/G	2109.2	329.5	0.5	6 m KOH	3 electrodes	[119]
3DG/N doped	702	405	1.0	6 m KOH	2 electrodes	[120]

### 3.2 Graphene‐Based Supercapacitors

Graphene, a 2D layered material, has been highly explored as the supercapacitor electrode due to its high surface area, chemical stability, and electrical conductivity.^[^ ^83^ ^]^ It exhibited an ambipolar electric field, which laid the foundation for developing graphene for supercapacitor applications.^[^ ^121^ ^]^ The specific capacitance of graphene was theoretically predicted to be 540 F g^−1^, much higher than that of other carbon materials such as activated carbon, carbon fibers, CNTs, and fullerenes. It is imperative to emphasize that the effective surface area of graphene is an important parameter to obtain a high specific capacitance based on theoretical calculations (up to 2630 m^2^ g^−1^).^[^ ^122^ ^]^ However, most graphene cannot exhibit high surface area due to the stacked layers. Therefore, delicately designing graphene‐based composites or doping with heteroatoms may enhance supercapacitor performance. In addition, obtaining porous graphene is another strategy for achieving high‐performance supercapacitors since the pores can facilitate ion diffusion. In addition, the treatment of graphene can further enhance its performance for supercapacitors, such as reducing internal resistance between the graphene sheets by laser treatment^[^ ^116^ ^]^ or enhancing the wettability by introducing functional groups.

Rao et al. demonstrated supercapacitors devices based on a few layers of graphene synthesized from graphitic oxide exfoliation and the conversion of nanodiamonds.^[^ ^123^ ^]^ In an aqueous electrolyte, the specific capacitance reached up to 117 F g^−1^, whereas the capacitance is around 75 F g^−1^ in an ionic liquid electrolyte with an energy density of 31.9 Wh kg^−1^. The specific capacitance obtained is higher than that of CNTs and highly depends on the number of layers, surface morphology, and surface area. Similar results were obtained in the case of chemically modified graphene synthesized by Ruoff et al.^[^ ^123^ ^]^ The highest capacitance achieved is around 135 F g^−1^ in aqueous and 99 F g^−1^ in organic electrolytes with an internal cell resistance as low as 0.15 ohm. Chemical activation of graphene with potassium hydroxide generated pores and increased the surface area up to 3100 m^2^ g^−1^, which showed a specific capacitance of 166 F g^−1^ with an energy density of 70 Wh kg^−1^ in an ionic liquid,^[^ ^123^ ^]^ indicating that activation‐induced pores could enhance the specific capacitance of graphene.

**Figure 4:**
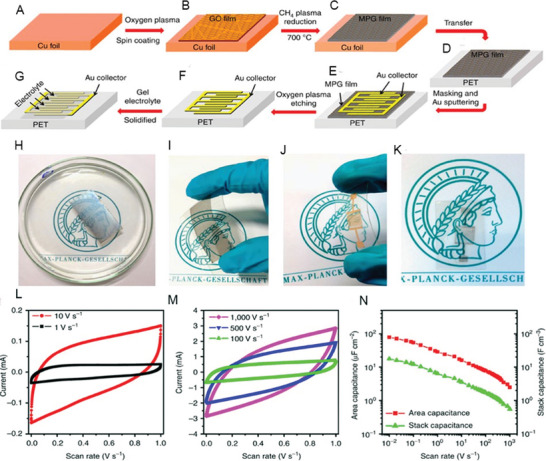
A–G) Schematic illustration of the fabrication of flexible MPG‐MSCs‐PET. The fabrication process includes a sequence of A) spin‐coating of GO solution on Cu foil, B) CH_4_ plasma reduction, C) transfer of MPG film from the Cu foil to PET substrate, D) masking pattern and deposition of gold current collector, E) oxidative etching, F) drop‐casting of H_2_SO4/PVA gel electrolyte and G) solidification of gel electrolyte. H–K) Optical images of H) a 15 nm thick MPG film (2 × 3 cm) on a polymethyl methacrylate (PMMA) support floated on the water surface after etching Cu foil by aqueous Fe(NO_3_)_3_ solution, I) the MPG film transferred onto the PET substrate, J,K) the resulting MPG‐MSCs‐PET J) with and K) without Au collectors, showing the flexible and transparent characteristics of the fabricated microdevices. L,M) CV curves of the MPG‐MSCs‐PET obtained at different scan rates from L) 1, 10 V and M) 100, 500, and 1,000 V s^−1^ with a typical electric double‐layer capacitive behavior, even at ultrahigh scan rates, demonstrating its ultrahigh power ability. N) Area capacitance and stack capacitance of the MPG‐MSCs‐PET. Reproduced with permission.^[^ ^126^ ^]^ Copyright 2013, Springer Nature.

Supercapacitors made with 1–2 layers of graphene as the electrode showed an areal capacitance up to 80 µF cm^−2^ whereas for a few‐layer graphene, 394 µF cm^−2^ (corresponding to 247.3 F g^−1^) was achieved in PVA/acid gel electrolyte, which is due to that more layers of graphene can store more charges in case of no agglomeration.^[^ ^124^ ^]^ Owing to the flexibility of graphene, a flexible 3D‐graphene hydrogel‐based device has also been demonstrated. The fabricated microsupercapacitor showed an areal capacitance of 372 mF cm^−2^ with low leakage resistance, high mechanical and cyclic stability.^[^ ^125^ ^]^ A descriptive scheme for making the microsupercapacitor device is shown in **Figure** **4**A–G.^[^ ^126^ ^]^ Figure 4H–K shows the flexibility and the transparent characteristics of the fabricated microsupercapacitors. Figure 4L,M shows the CV curves measured at different scan rates. The microdevice showed an areal capacitance of 78.9 µF cm^−2^ at 10 mV s^−1^ with an energy and power density of 2.5 mWh cm^−3^ and 495 W cm^−3^, respectively (Figure 4N), demonstrating the promising applications of graphene.

Graphene sheets tend to agglomerate owing to van der Waals forces interaction, hence, the electrolyte ions do not have enough space to move around between the electrodes. In order to decrease the agglomeration in graphene sheets, Wang et al.^[^ ^127^ ^]^ synthesized graphene by reducing graphene oxide using hydrazine hydrate to alleviate the agglomeration effect, which showed a specific capacitance of 205 F g^−1^ in an aqueous electrolyte with an energy and power density of 28.5 Wh kg^−1^ and 10 kW kg^−1^, respectively, higher than that of CNT‐based counterparts.^[^ ^128^ ^]^ The possible reason for the CNTs exhibiting a lower capacitance could attribute to the relatively high internal resistance arising from poor contact between the current collector and the working electrode. Graphene sheets with ultrahigh energy density were obtained by Jang et al.^[^ ^129^ ^]^ using the same reducing graphene oxide method. The synthesized graphene as electrode showed a specific capacitance of 250 F g^−1^ with an energy density as high as 85.6 Wh kg^−1^, which is very close to that of lead acid batteries and Ni hydride batteries. Although the specific capacitance of graphene is higher than those of CNTs or fullerenes, the observed capacitance is still far away from the theoretical capacity. The intrinsic capacitance of graphene can be enhanced by making composites with pseudocapacitive materials like conducting polymers or metal oxides. Macroporous frameworks with 3D architecture are proposed as an effective solution to the agglomeration of graphene by Kaner and co‐workers in 2022.^[^ ^130^ ^]^ In this work, a cationic surfactant was utilized to improve the adsorption of GO on electrodes. Through this route, the direct deposition of individual functionalized graphene nanosheets into 3D architecture was succeeded with large electrochemically active surface areas. The resultant supercapacitor has a high specific capacitance of 320 F g^−1^, a low internal resistance of 1 Ω cm^−2^, and good retention stability without performance degradation after 10 000 cycles. Very recently, Qu's group designed a novel spatial‐interleaving supercapacitor (SI‐SC) constructed with graphene microelectrodes that are stacked layer by layer in a 3D space.^[^ ^131^ ^]^ It is designed to have high mechanical flexibility and power density, and overcome the limitations of current supercapacitor designs. The special interleaving design and narrow interspaces between the microelectrodes ensure efficient ion transport and result in a high specific areal capacitance of 36.46 mF cm^–2^ and energy density of 5.34 µWh cm^–2^. The SI‐SC shows decent mechanical stability, with 98.7% performance retention even after 10000 bending tests, demonstrating high potential in wearable electronics. In addition, graphene aerogels can be made to address the surface area concerns, which are presently considered one of the lightest materials in the world.^[^ ^132^, ^133^, ^134^, ^135^ ^]^ Especially, graphene aerogels have been widely employed in supercapacitor applications.^[^ ^136^, ^137^, ^138^, ^139^ ^]^ Thus, high surface area mesoporous structures with enhanced electrical conductivity in general are needed for superior supercapacitive performance. Sufficient electrochemically active sites in these structures are suitable for fast cycling of reversible Faradaic redox.

### 3.3 Graphene/Conducting Polymer Composites‐Based Supercapacitors

Flexible electronic devices have attracted extensive attention due to their wearable and portable features. Consequently, flexible energy devices are urgently required. Chemically reduced graphene oxide (GO) composites with polyaniline exhibited excellent supercapacitor properties and good mechanical stability and flexibility.^[^ ^140^ ^]^ The specific capacitance achieved is about 210 F g^−1^ at 0.3 A g^−1^ with excellent electrical conductivity (5.5 × 10^2^ S m^−1^), which is almost 10 times higher than that of polyaniline fibers. Similarly, graphene layers functionalized by in situ anodic polymerization of aniline showed a mechanical strength of 12.6 MPa and volumetric capacitance of 233 F g^−1^.^[^ ^141^ ^]^ These characteristic performances are much better than that of other carbon‐based polyaniline composites. The surface‐modified graphene with polyaniline (PANI) could exhibit fast redox capabilities.^[^ ^142^ ^]^ Likely, Lai et al. synthesized reduced GO doped with nitrogen first, and subsequently reduced the doped GO into graphene, finalized by the functionalization with the amine groups.^[^ ^143^ ^]^ It was found that the amine‐containing reduced GO, loaded with PANI, exhibited a higher capacitance compared to the doped graphene. The role of amine functionality can also be understood through the work reported by Song et al.^[^ ^144^ ^]^ PANI nanofibers synthesized by Zhang et al.^[^ ^145^ ^]^ and Mao et al.^[^ ^146^ ^]^ revealed that PANI helped in stabilizing GO even in acidic conditions, and the fabricated supercapacitor showed a specific capacitance value of 480 F g^−1^ at 0.1 A g^−1^, whereas sulfonated PANI displayed a specific capacitance around 157 F g^−1^ at 1.5 A g^−1^.^[^ ^147^ ^]^ PANI in combination with reduced GO without the involvement of any further functional groups also resulted in better capacitance comparatively.^[^ ^148^, ^149^, ^150^, ^151^, ^152^, ^153^, ^154^ ^]^ Other than PANI, various other conducting polymers^[^ ^155^ ^]^ like polypyrole^[^ ^156^, ^157^ ^]^ based materials have shown fascinating properties as flexible electrodes that is paving a path toward commercial ultracapacitors.^[^ ^158^, ^159^, ^160^ ^]^ The role of available functionalities in conducting polymers in providing conducting channels is significant in determining the super‐capacitive performance. Nevertheless, functionalized and doped graphene materials are continuously gaining popularity due to their fast electron transfer with faradaic reactions, which still motivates the scientific community to develop new composite materials.^[^ ^161^, ^162^, ^163^ ^]^ Surface area maximization by introducing mesoporosity with uniform pore diameter can further enhance performance.

### 3.4 Doped Graphene‐Based Supercapacitors

Doping graphene with heteroatoms, especially non‐metal elements such as N, B, S, and P, has shown interesting physical and electrochemical properties. These heteroatoms were doped differently in the graphene lattice depending on the nature of the dopants. The electronic properties of the graphene can be tuned by the dopant concentrations. Among these heteroatoms, nitrogen and boron have been extensively studied because of their similar atomic sizes to carbon, and their doping has also shown interesting electrochemical and electrical properties. Doped graphene has been synthesized in various approaches ranging from CVD, plasma and microwave irradiation, wet chemical methods, solvothermal and hydrothermal methods. These synthesis procedures yield doping concentrations at different levels.

#### 3.4.1 N‐Doped Graphene

Nitrogen is a common dopant for graphene, which can be doped into graphene lattice at different configurations. The probable nitrogen configurations can be pyridinic, pyrrolic, or amine. In one representative work, Qiu et al.^[^ ^164^ ^]^ treated reduced GO in the presence of NH_3_ at 700 °C to synthesize nitrogen‐doped graphene (2 at% of N) with a high electrical conductivity and thermal stability. The produced N‐doped graphene showed a specific capacitance of 145 F g^−1^ at 1 A g^−1^ with an operational potential window of 0–4 V. In another example, a high concentration of nitrogen (10 at%) containing graphene sheets prepared using one‐pot synthesis by hydrothermal treatment of graphene oxide and urea showed a specific capacitance of 289 F g^−1^ at 0.2 A g^−1^.^[^ ^165^ ^]^ It was observed that the high amount of quaternary nitrogens presented in the graphene increased the electrical conductivity. The pyrrolic and pyridinic nitrogens generated pseudo‐capacitance and increased the overall specific capacitance of the material. Similarly, when NH_3_ was used as the nitrogen source under hydrothermal conditions, N‐doped graphene exhibited a specific capacitance of 144 F g^−1^.^[^ ^166^ ^]^ The interfacial capacitance of nitrogen‐doped graphene increased from 6 to 22 µF cm^−2^ when the nitrogen content increased from 0 to 2.3 at%.^[^ ^167^ ^]^ The increase in capacitance is due to the change in the electronic properties of graphene after nitrogen doping.

When hexamethylenetetramine was used as the nitrogen source, it simultaneously reduced GO and doped nitrogen up to 8.6 at%.^[^ ^168^ ^]^ The specific capacitance achieved is about 161 F g^−1^ at 0.5 A g^−1^, and the supercapacitor also exhibited excellent cyclic stability. The performance of nitrogen‐doped graphene can be further enhanced by delicate control of morphology and surface area. Elessawy et al. prepared 3D sponge N doped graphene using waste polyethylene‐terephthalate (PET) bottles mixed with urea at different temperatures, achieving a specific capacitance of 405 F g^−1^ at 1A g^−1^ with high power (558.5 kg^−1^) and energy density (68.1 Wh Kg^−1^).^[^ ^120^ ^]^ Likewise, Wen et al. synthesized crumbled nitrogen‐doped graphene by treating cyanamide and GO at 900 °C, obtaining a nitrogen doping concentration of 8.2 wt% and a very large pore volume of 3.42 cm^3^ g^−1^. The specific capacitance of this material is about 248 F g^−1^ which is about four times higher than that of undoped graphene.^[^ ^169^ ^]^

Activation is another well‐established strategy to increase the surface area. To this end, Peng et al. synthesized nitrogen‐doped graphene using ammonia gas for simultaneous reduction and doping of GO, followed by CaCl_2_ activation.^[^ ^170^ ^]^ The obtained crumpled nitrogen‐doped graphene possessed a surface area of 1169 m^2^ g^−1^ and exhibited a specific capacitance of 294 F g^−1^ at 0.5 A g^−1^. The enhancement in the capacitance comes from the expansion of graphene layers during ammonia gasification, in which the chemical activation increases the pore volume and the surface area. The unique structure of these graphene sheets with large pore volumes facilitates the mobility of the electrolyte ions and also improves electrical conductivity to achieve high specific capacitance. Hassan et al. synthesized doped graphene with a large amount of pyrrolic nitrogen by a hydrothermal method using hydrazine at different temperatures.^[^ ^171^ ^]^ Undoped graphene was synthesized using NaBH_4_ for comparison. The specific capacitance of doped graphene is around 194 F g^−1^, much higher than undoped graphene.

**Figure 5:**
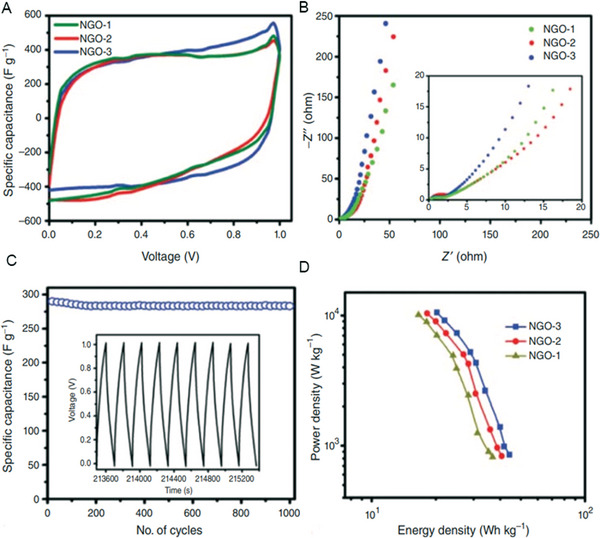
A) Cyclic voltammograms of NGOs at a scan rate of 20 mV s^−1^. B) Nyquist curves for NGO electrodes. C) Specific capacitance versus the cycle number of NGO measured at a current density of 0.5 A g^−1^ within an operational window of 0.0–1 V (the inset shows the charge‐discharge curves of the last few cycles for NGO). D) Ragone plots of NGO‐based supercapacitors. Reproduced with permission.^[^ ^174^ ^]^ Copyright 2013, Royal Society of Chemistry.

Nitrogen‐doped GO also demonstrated promising properties for supercapacitors.^[^ ^172^ ^]^ For example, Xu et al. proposed a freezing casting method to fabricate nitrogen‐doped graphene oxide film with high porosity.^[^ ^173^ ^]^ The material as electrode material for supercapacitors showed a high specific capacity of 528 F g^−1^ at 1A g^−1^ with excellent cycling stability. Rao et al. synthesized heavily nitrogen‐doped GO by a microwave method using urea as a nitrogen source.^[^ ^174^ ^]^ In this reaction, the reduction of graphene oxide and nitrogen doping occurred simultaneously to yield up to 18 wt% of nitrogen content with a high specific capacitance of 461 F g^−1^ in an aqueous electrolyte. **Figure** **5**A shows the CV curves of the nitrogen‐doped GO samples, which show the characteristic features of an ideal capacitor. The Nyquist plots in Figure 5B reveal that the conductivity of the samples improves with the increase in the nitrogen content, and Figure 5C shows the cyclic stability measurements. These nitrogen‐doped graphene oxide (NGO) electrodes showed distinct cyclic stability and also exhibited high energy density and power density (Figure 5D). The authors claimed that the high specific capacitance is mainly due to the pseudo‐capacitance arising from the pyrrolic and pyridinic nitrogens presented in the material.

Nitrogen‐doped graphene can also be made into hydrogels. For example, hydrogels of nitrogen‐doped graphene synthesized using various organic amines showed a typical specific capacitance of 190.1 F g^−1^ and an energy density of 245.0 kW kg^−1^ at 10 A g^−1^.^[^ ^175^ ^]^ These hydrogel‐based supercapacitors exhibited extraordinary capacitance retention even at high current densities. At 100 and 200 A g^−1^, the fabricated devices presented a specific capacitance of 69.1 and 28.8 F g^−1^, respectively. Further studies disclosed that the organic amines reduced the carboxyl functional groups and formed a stable nitrogen‐doped graphene hydrogel because of the *π*–*π* interactions. Notably, these functionalized amine groups not only control the agglomeration of the graphene sheets but also allow the electrolyte ions to move freely between the layers and thus increase the overall capacitance. Similarly, chemical functionalization of graphene with ethylenediamine containing 9.8 at.% nitrogen content showed an enhanced pseudocapacitive behavior with a specific capacitance of 364.6 F g^−1^ at 10 mV s^−1^, which is three times higher than that of pure graphene.^[^ ^176^ ^]^ Even higher nitrogen‐doped graphene (10.8 at%) hydrogels were synthesized, which exhibited a specific capacitance of 194 F g^−1^ and a high energy density of 94.5 Wh kg^−1^.^[^ ^177^ ^]^

Supercritical fluid^[^ ^178^ ^]^ assisted synthesis of nitrogen‐doped graphene showed a specific capacitance up to 286 F g^−1^ at 0.5 A g^−1^ in aqueous electrolyte, whereas when synthesized on Ni foam, N‐doped graphene exhibited 184 F g^−1^ at 35.7 A g^−1^ and the capacitance increases to 204 F g^−1^ after cycling for 2000 cycles.^[^ ^179^ ^]^ Hydrothermally synthesized hydrogels containing 7.7 wt% nitrogen content showed a specific capacitance of 387.2 F g^−1^ at a current density of 1 A g^−1^ in an aqueous electrolyte.^[^ ^180^ ^]^ Capacitance retention of 90.5% was obtained after charging and discharging at 5 A g^−1^ for 5000 cycles. The high content of pyrrolic and pyridinic nitrogens is attributed to the increase in capacitance and stability of the material. Zhao et al. fabricated binder‐free electrodes based on N‐doped graphene hydrogels synthesized by treating GO with ammonia, which achieved a high N‐doping concentration of 7.1 at%.^[^ ^181^ ^]^ The doped graphene consisted of large cross‐linked pores that facilitated the electrolyte ion motion between the electrodes and showed a specific capacitance of 334 F g^−1^ at 0.5 A g^−1^. The high performance of hydrogels for supercapacitors can be further improved by functionalization. Zou et al. reported a modified hydrothermal method to synthesize nitrogen‐doped graphene hydrogel using amines, which led to a nitrogen doping concentration ranging from 7 to 11 at%.^[^ ^182^ ^]^ This amine‐based reduction of GO yielded a high content of —NH_2_ functionality, which increased the interaction between the anode materials and the electrolyte ions as well as the wettability of the electrode. With a wide range of nitrogen doping concentrations, these electrodes showed extremely high specific capacitance ranging from 467 to 645 F g^−1^ at 1 A g^−1^ and capacitance retention up to 83.1% after 1000 cycles. A similar report on nitrogen‐doped graphene hydrogels by another group showed a specific capacitance of 335 F g^−1^ at 1 A g^−1^ with an energy density of 58.1 Wh kg^−1^.^[^ ^183^ ^]^ Apart from hydrogel, highly nitrogen‐doped graphene aerogel with a nitrogen content of 15.8 at% was fabricated, which exhibited a surface area of 583 m^2^ g^−1^ and specific capacitance up to 380 F g^−1^ in multiple electrolytes.^[^ ^184^ ^]^ When measured in a two‐electrode configuration, the nitrogen‐doped graphene showed a specific capacitance of 297 F g^−1^ in an alkaline electrolyte solution. By carbonizing and activating shrimp shells, Tian et al. produced high energy density supercapacitor electrodes.^[^ ^185^ ^]^ These electrodes possessed high surface areas, a high nitrogen content (8.75 at%) and excellent electrical conductance, which led to a high supercapacitor performance with a specific capacitance of 322 F g^−1^ and excellent cyclic stability with an energy density of 30 Wh kg^−​1^. This method produced an even higher specific capacitance supercapacitor by simultaneously carbonizing and activating an ion‐exchange resin with melamine, nickel acetate, and KOH. The synthesized nitrogen‐doped graphene has a surface area of 1815 m^−2^ g^−1^ with a nitrogen doping concentration of 3.62 at%.^[^ ^186^ ^]^ An aqueous electrolyte solution showed a specific capacitance of 383 F g^‐1^ with 98% initial capacitance retention.

Treating graphene with amine has advantages for nitrogen doping and the formation of a 3D structure, as a result, hindering the agglomeration of graphene flakes.^[^ ^187^ ^]^ These N‐doped graphene sheets showed a high specific capacitance of 408 F g^−1^. When treated graphene oxide with amitrole, it yielded high pyridinic nitrogen along with slightly lower pyrrolic and quaternary nitrogen configuration.^[^ ^188^ ^]^ This synthesis method yielded around 13.4 at% of nitrogen doping and a specific capacitance of 244 F g^−1^. As discussed previously, the high performance of supercapacitors is associated with pyridinic nitrogen, which provides the active sites for energy storage. The precursors may provide both nitrogen sources and active sites by heating to a certain temperature. For example, bubble‐like structured nitrogen‐doped graphene was obtained by heating the mixture of GO and melamine, which acted as the nitrogen precursor.^[^ ^189^ ^]^ Heat treatment generated both nitrogen doping and lots of active sites for the electrolyte ions to flow freely between the graphene sheets. When tested as a supercapacitor electrode, it showed a specific capacitance of 481 F g^−1^ at 1 A g^−1^, which is much higher than that of undoped graphene. In addition, it also showed high energy density and cyclic stability in acid electrolytes.

Furthermore, nitrogen‐doped graphene has been synthesized with a few other methodologies. For instance, nitrogen‐doped graphene was derived from zinc‐based MOFs such as ZIF‐8.^[^ ^190^ ^]^ ZIF‐8 was thermally treated to obtain N‐doped graphene decorated with carbon nanoparticles with a size range of 30–50 nm and a surface area of 816.4 m^2^ g^−1^. These electrodes showed a typical specific capacitance of 225 F g^−1^ at 0.5 A g^−1^ with an energy and power density of 12.7 Wh kg^−1^ and 447 W kg^−1^, respectively. Sathish et al. used a supercritical process to synthesize N‐doped graphene with ammonium oxalate and graphene oxide. A specific capacitance of 160 F g^‐1^ at 1 A g^‐1^ was achieved in 20% KOH solution.^[^ ^191^ ^]^

#### 3.4.2 B‐Doped Graphene

Like nitrogen‐doped graphene, boron‐doped graphene has also shown interesting electrochemical properties.^[^ ^192^ ^]^ Boron has a similar atomic size (85 pm) to that of carbon (70 pm). When boron is doped in graphene, an ambipolar behavior can be expected. Niu et al. prepared boron‐doped graphene by mixing graphene oxide and boric acid together with annealing at 900 °C for 3 h.^[^ ^193^ ^]^ The doping concentration is around 4.7 at%, and the doped graphene as the electrode achieved a specific capacitance of 175 F g^−1^ with 80% enhancement of capacitance as compared to the undoped graphene. The annealing temperature strongly influences the doping concentration and supercapacitor performance. When the mixture of boric acid and GO was treated at 500 °C, the doping concentration was approximately 2.56 at% with a specific capacitance of 113 F g^−1^ at 1 A g^−1^.^[^ ^194^ ^]^ In addition, when graphene oxide mixed with boron oxide was heated at 1000 °C, the doping concentration could reach 6 at%. A much higher specific capacitance of 448 F g^−1^ was achieved,^[^ ^195^ ^]^ while the undoped graphene only possessed a specific capacitance of 135 F g^−1^, confirming the temperature treatment effect on both the doping concentration and capacitance. The enhanced capacitance could be attributed to the reduced charge transfer resistance by boron doping.

Besides doping concentration, the porous structure can further enhance capacitance. Porous boron‐doped graphene was synthesized by Zuo et al. using a Fried‐Ice method, which showed a doping concentration of 3 at% and a specific capacitance of 281 F g^−1^,^[^ ^196^ ^]^ higher than those without porosity but with similar doping concentration. Furthermore, doping by the chemical solution method has also attracted extensive attention since the process can reduce costs and easily obtain a large surface area. Han et al. reduced GO using borane (BH_3_)‐tetrahydrofuran (THF) as the dopant precursors in the solution.^[^ ^197^ ^]^ The B‐doped graphene nanoplatelets possessed a surface area of about 466 m^2^ g^−1^ and exhibited a specific capacitance of 200 F g^−1^ at 50 mV s^−1^. The good electrochemical performance of the doped graphene is due to the improved electric conductivity and high surface area, which enhances the electrolyte ion adsorption and facilitates the redox reactions over the B‐doped graphene nanoplatelets.

**Figure 6:**
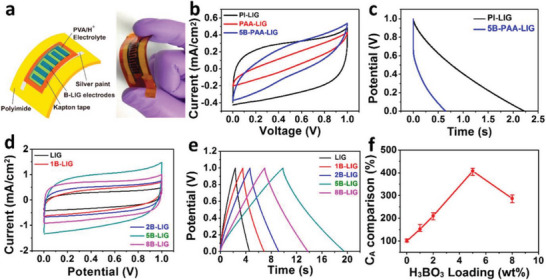
Electrochemical performance comparison of laser induced graphene‐mcirosucpercapacitors (LIG‐MSCs) with different H_3_BO_3_ loadings. A) Schematic of a B‐LIG‐MSC device and the digital photograph of a fully fabricated device under bending. B) CV curves of MSCs from polyimide (PI)‐derived LIG, PAA‐derived LIG, and PAA/H_3_BO_3_‐derived LIG at a scan rate of 0.1 V s^‐1^. C) CC curves of MSCs from PI‐derived LIG and PAA/H_3_BO_3_‐derived LIG at a current density of 1.0 mA cm^‐2^. (D) CV curves of LIG‐MSC and B‐LIG‐MSC with different H_3_BO_3_ loadings. The scan rate is set at 0.1 V s^‐1^. E) Galvanostatic CC curves of LIG‐MSC and B‐LIG‐MSC with different H_3_BO_3_ loadings. The current density is set at 1 mA cm^‐2^. F) Comparison of calculated *C*_A_ from LIG‐MSC and B‐LIG‐MSC with different H_3_BO_3_ loadings. Current density is 1 mA cm^‐2^. At least three devices were tested for each *x*B‐LIG sample. Reproduced with permission.^[^ ^198^ ^]^ Copyright 2015, American Chemical Society.

Plasma‐assisted doping is another interesting and effective method for introducing functional moieties inside the carbon framework of graphene nanostructures. Li et al. used dielectric barrier discharge (DBD) plasma to treat the mixture of reduced GO and boric acid for three minutes and achieved a boron doping concentration of 1.4 at%.^[^ ^181^ ^]^ The obtained boron‐doped graphene showed excellent supercapacitor properties with a specific capacitance of 446.24 F g^−1^ at 0.5 A g^−1^, much higher than pristine graphene and most of the doped graphene synthesized by other methods. Another approach to induce doping by laser induction has been used for the fabrication of flexible supercapacitors. The flexibility of graphene has attracted extensive interest in flexible electronics. As the electrode, the doped graphene synthesized using this method could achieve an areal capacitance of 6.5 mF cm^−2^ at the optimal condition, which is three times higher than that of undoped graphene, as shown in **Figure** **6**.^[^ ^198^ ^]^ Figure 6A shows the structure of an interdigital microsupercapacitor device. CV curves and the charge–discharge curves are displayed in Figure 6B–E, and the effect of boron concentration is shown in Figure 6F.

Besides the fabrication approaches, the electrolyte also plays an important role in capacitor performance. In a typical example, Sathish et al. measured the performance of boron‐doped graphene synthesized using a supercritical fluid process in both aqueous and ionic liquid electrolytes.^[^ ^199^ ^]^ In 20% KOH electrolyte, the fabricated electrode showed a specific capacitance of 270 F g^−1^ at 1 A g^−1^, but the energy density obtained in an ionic liquid electrolyte (39.3 Wh kg^−1^) is much higher than that in 20% KOH (5.1 Wh kg^−1^). As discussed previously, the reduced charge transfer resistance, high surface area, and improved conductivity are attributed to the enhanced capacitance after boron doping. However, studies revealed that the boron configurations (BCO_2_ and BC_2_O) and the content of oxygen functionalities in the graphene lattice strongly influenced the supercapacitor performance.^[^ ^200^ ^]^ Higher concentrations of dopant and oxygen functionalities in the graphene nanostructure can offer higher specific capacitance (336 F g^−1^) compared to the lower concentrations of those in graphene (169 F g^−1^ at 0.1 A g^−1^).

B and N codoped graphene for the applications of supercapacitors can be traced back to 2012. Mullen et al.^[^ ^201^ ^]^ made N, B‐doped graphene‐based solid‐state supercapacitor devices without adding any additional conductive carbon and binders. These flexible thin devices exhibited very high supercapacitor performance with a specific capacitance of 62 F g^−1^, high energy density of 8.65 Wh kg^−1^, as well as excellent stability. The high performance of the supercapacitor could be ascribed to the presence of hetero atoms in the graphene lattice, which improves the electrical conductivity between the adjacent carbon atoms and thus increases the specific capacitance. When reducing graphene oxide using ammonia borane, B, N‐doped GO showed a specific capacitance of 130 F g^−1^ at 1 A g^−1^ in organic electrolytes.^[^ ^202^ ^]^ This electrode showed low ohmic and charge transfer resistance. At a high current density of 8 A g^−1^, this electrode exhibited a 13.8% capacitance loss (112 F g^−1^) compared to that work at 1 A g^−1^ and excellent cyclic stability was evidenced by retaining 95.4% capacitance even after 1000 cycles. It was also found that ammonia borane is a better reducing agent than hydrazine hydrate and other organic reducing agents.

Chen et al. synthesized B, N‐doped graphene in gram scale thorough a two‐step process. Firstly, graphite was ball‐milled under a nitrogen atmosphere to synthesize N‐doped graphene. Then, the milled graphene was calcined at a variety of temperatures with boric acid to fabricate B,N‐doped graphene with a boron doping concentration of 2.19 at%. The obtained product exhibited a high surface area of 802 m^2^ g^−1^ and a specific capacitance of 254 F g^−1^ at 0.25 A g^−1^ and excellent stability with 98.2% capacitance retention at a current density of 100 A g^−1^. A high surface area was also obtained in B, N‐doped graphene by the formation of aerogels synthesized using ammonia borane. The co‐doped graphene showed excellent electrochemical performance.^[^ ^203^ ^]^ Under a three‐electrode configuration, the doped graphene as electrodes showed a specific capacitance as high as 456 F g^−1^ at 1 A g^−1^. Moreover, solid‐state flexible supercapacitor devices based on these electrodes showed an areal capacitance of 345 mF cm^−2^ at 1 mA cm^−2^ with remarkable capacitance retention of 80% even under strained situations. The high capacitance may be attributed to the additional pseudo‐capacitance besides the double‐layer capacitance arising from the redox B, N functionalities. The high specific capacitance, stability, and ability to work under extreme conditions make B doping a promising strategy for fabricating high‐performance supercapacitors.

#### 3.4.3 S‐Doped Graphene

In addition to nitrogen and boron doping in graphene, sulfur doping has also significantly changed the graphene structure and electronic properties.^[^ ^204^, ^205^ ^]^ Not many works on the research of graphene by sulfur doping were reported a decade ago, but researchers have recently found that sulfur doping can create pseudocapacitive behavior in graphene in addition to the EDLC,^[^ ^205^ ^]^ which can be attributed to the oxidized sulfur functionalities created during the doping process. In addition, the polarity of carbon in the graphene lattice becomes slightly more positive because of the sulfur atoms doping. Thus, sulfur doping in graphene can increase the overall specific capacitance of the material.

With all these observations, there are several reports based on sulfur doping and its applications in the field of supercapacitors. For example, Bandosz et al.^[^ ^206^ ^]^ carbonized the polymers of sulfonates and graphite oxide to form a composite of porous carbon and sulfur‐doped graphene for supercapacitor applications, yielding a specific capacitance of 157 F g^−1^ at 50 mA g^−1^. The good supercapacitor performance can be attributed to redox reactions arising from the sulfur oxide functionalities and the pores generated during the carbonization process. Chen et al.^[^ ^207^ ^]^ doped sulfur into porous reduced GO using dibenzyl disulfide to obtain a specific capacitance of 343 F g^−1^. The resultant electrode showed very good chemical and cyclic stability in alkaline electrolyte solutions. In another report, electrochemical doping of sulfur and the simultaneous exfoliation of graphite were performed using sodium thiosulfate as the sulfur precursor, producing highly dispersed solutions of sulfur‐doped graphene nanosheets with a sulfur doping concentration of 3.47 at%.^[^ ^208^ ^]^ This electrode showed a specific capacitor of 320 F g^−1^ at 3 A g^−1^ with an excellent energy density and power density of 160 Wh kg^−1^ and 5161 W kg^−1^, respectively, in alkaline electrolyte solutions.

Electron donating molecule like tetrathiafulvalene (TTF) has also been used as a sulfur source as well as a reducing agent for doping sulfur into graphene.^[^ ^209^ ^]^ In this reaction, TTF was reacted with GO under refluxing conditions for 6 h to produce a 3D structure of S‐doped graphene with a sulfur content of 26 at%, which is almost the highest doping concentration reported. The electrode fabricated from this material exhibited a specific capacitance of 212 F g^−1^ at 0.3 A g^−1^ in aqueous electrolytes. Though the capacitance is not very high, at higher current densities, this electrode performed reasonably well by retaining 69% of the capacitance at 20 A g^−1^ and 98% of the capacitance after cycling for 4000 cycles at 1 A g^−1^. From this example, it is known that the doping amount does not definitely determine the value of capacitance. The preparation process is also of importance. In a single‐step process, GO was simultaneously reduced and doped with sulfur using sodium sulfide with a doping concentration of 0.37 at%. The doping concentration is very low, whereas, from the constant current charge–discharge measurements, the specific capacitance obtained is around 392 F g^−1^ at 0.05 mA cm^−2^ with an energy density of 44.1 Wh kg^−1^, higher than most of the reported specific capacitance by sulfur doping. Besides sulfur doping‐induced fast kinetics, the functional groups may also contribute significantly to the capacitance. Zhang et al.^[^ ^210^ ^]^ studied the electrochemical property of the surface oxidized sulfur species and studied the cyclic stability of the sulfur‐doped graphene nanosheets by cycling the electrodes for more than 10 000 cycles at 50 mV s^−1^. They post‐treated the graphene nanosheets with sulfuric acid in order to create oxidized sulfur species in addition to the doped sulfur atoms. These oxidized sulfur functional groups added excess pseudo‐capacitance to the sulfur‐doped graphene electrodes to show a specific capacitance of 270 F g^−1^ at 5 mV s^−1^.

Besides sulfur doping alone, co‐doping nitrogen and sulfur into graphene is another effective strategy to increase the overall supercapacitor performance.^[^ ^211^ ^]^ Xing et al. used thiocarbohydrazide as both the nitrogen and sulfur source as well as a reducing agent to produce N, S co‐doped graphene.^[^ ^212^ ^]^ The structure after doping is 3D with high porosity and a doping concentration of 6.6 at% nitrogen and 6 at% sulfur, respectively. When tested as a supercapacitor electrode, the supercapacitor showed a specific capacitance of 141 F g^−1^ at 0.3 A g^−1^ in alkaline electrolyte with good stability at high current densities even after cycling for 4000 cycles. In another case, Chen et al.^[^ ^213^ ^]^ fabricated supercapacitor electrodes based on N, S‐ co‐doped graphene synthesized using a single precursor (1‐amino‐2‐thiourea). The interlayers of synthesized co‐doped graphene were loosely bound with a feather‐like structure, allowing faster mobilization of electrolyte ions and thus increasing the supercapacitor performance with a specific capacitance of 302 F g^−1^ at 5 mV s^−1^. Compared with single S doping, it seems that the cycling stability is improved. Several methods are employed for codoping nitrogen and sulfur, and these methods and the optimized parameters strongly influence the supercapacitor performance.

CVD and solvothermal/hydrothermal methods are common approaches for synthesizing doped graphene due to their relative ease of doping. GO itself contains a lot of oxygen functional groups; hence, replacing a few oxygen groups with nitrogen is not difficult using a CVD or solvothermal/hydrothermal method. However, co‐doping both nitrogen and sulfur is complicated, making doping control difficult. Wang et al.^[^ ^214^ ^]^ investigated the synthesis of N, S‐co‐doped graphene by treating graphene oxide with a combination of amino acid and L‐cysteine using a hydrothermal method, which introduced 1.3 at% nitrogen and 3.2 at% sulfur doping. These electrodes showed a specific capacitance of 385 F g^−1^ at 0.5 A g^−1^ using a three‐electrode configuration and an energy density of 193 F g^−1^ at 100 mV s^−1^ calculated based on a two‐electrode configuration. The capacitor still exhibited 93% retention of the initial capacitance with an energy and power density of 29.4 Wh kg^−1^ and 10 kW kg^−1^, respectively, even after cycling for more than 2000 cycles. Similar to other carbon‐based materials, the amount of dopant concentration, surface area, and porosity all play important roles in the supercapacitor performance. Tran et al.^[^ ^215^ ^]^ studied the structural change during the doping process and how the doping concentration of nitrogen and sulfur affected the electrochemical performance. Lots of hole defects were observed during the doping process. The hole defects could be created by treating GO with hydrogen peroxide and then treated with vitamin C solution in an autoclave of 12 h at 180 °C, yielding N, S‐co‐doped graphene containing 2.49 at% sulfur and 1.12 at% nitrogen. The electrochemical specific capacitance obtained is around 536 F g^−1^ at 5 mV s^−1^ with an energy density of 14.8 Wh kg^−1^ based on solid‐state supercapacitor devices. This extremely high specific capacitance may attribute to the reduced charge‐transfer resistance and high surface area, facilitating the mobilization of electrolyte ions, leading to the enhancement of pseudocapacitive behavior. CVD is one of the most reliable methods for growing graphene. Hence, it is widely used for the synthesis of doped graphene. A typical example is the N,S doped graphene fibers synthesized by a CVD method, which showed a specific capacitance of 311 F g^−1^ and energy density of 37.7 Wh Kg^−1^.^[^ ^216^ ^]^

In another report, graphene oxide was treated with different proportions of aminothiourea or aminourea to produce N, S‐doped graphene with different nitrogen and sulfur doping concentrations.^[^ ^217^ ^]^ These electrodes performed extremely well with a gravimetric capacitance of 345 F g^−1^ at 200 mA g^−1^ in aqueous electrolytes. Whereas porous N,S‐co‐doped graphene aerogel showed a very high energy density of 100.7 Wh kg^−1^ in an ionic liquid with a gravimetric capacitance of 203 F g^−1^.^[^ ^218^ ^]^ Furthermore, these electrodes showed impressive stability even after cycling for more than 3000 cycles. The increase in the performance can be ascribed to the porous nature of the electrode, which facilitates the fast mobilization of the electrolyte ions. Even higher energy density was obtained from bio‐mass derived N, S‐graphene‐like nanosheets. These graphene‐like nanosheets derived from carbonizing coir pith exhibited a specific capacity of 247 F g^−1^ at 200 mA g^−1^. In addition, the supercapacitor exhibited an energy density of 33.6 Wh kg^−1^ at low current densities and a power density of 4220 Wh kg^−1^ at high current densities.^[^ ^219^ ^]^ Electron‐beam‐assisted synthesis of N, S‐doped graphene is an interesting method for doping, which yielded about ≈10 at% and ≈4 at% of nitrogen and sulfur, respectively. The high energy electron beam simultaneously reduced the graphene oxide and also doped the nitrogen and sulfur using L‐cysteine as the doping source.^[^ ^220^ ^]^ The doping concentrations of nitrogen and sulfur were controlled by varying the dose of the electron beam. GO irradiated with 210 kGy showed the highest electrochemical performance with a special capacity of 228 F g^−1^ at 10 mVs and possessed almost 83% retention of the initial capacitance after a long time of cycling. Other methods, such as inductive couple plasma, were also utilized to synthesize N, S‐co‐doped graphene.^[^ ^221^ ^]^ Moreover, plasma was used for removing the unwanted functional groups for the nitrogen and sulfur‐codoped graphene by a hydrothermal method. The doped graphene after plasma treatment exhibited an excellent electrochemical supercapacitor performance with a specific capacitance of 307 F g^−1^ and an energy density of 9.3 Wh kg^−1^, much higher than those without treatment. Besides the various synthesis approaches, various precursors like mercaptobenzimidazole^[^ ^222^ ^]^ and a combination of ammonia/thiourea^[^ ^223^ ^]^ were also used for the synthesis of N, S‐co‐doped graphene for supercapacitor applications, which evidently influences the performance of the supercapacitors.

#### 3.4.4 P‐Doped Graphene

Similar to sulfur and nitrogen, phosphorus has been employed to be doped into graphene lattice in order to increase the supercapacitor performance.^[^ ^224^, ^225^, ^226^ ^]^ N and P are the same column in the periodic table with the same number of valence electrons. Doping phosphorous can change the electrochemical properties of graphene and electronic properties. The P‐doped graphene was reported to be an excellent catalyst for oxygen reduction reactions or as an anode for secondary batteries.^[^ ^227^ ^]^ For supercapacitors, Wen et al. synthesized P‐doped graphene using phosphoric acid annealed at a relatively high temperature to achieve a doping concentration of 1.3 at%.^[^ ^228^ ^]^ After the P‐doing, the doped graphene showed excellent performance with a specific capacitance of 115 F g^−1^ at 50 mA g^−1^ in an aqueous 1 m sulfuric acid electrolyte. The electrode made from P‐doped graphene was able to be operated at a slightly high voltage of 1.7 V, where the cyclic voltammogram curves exhibited quasi‐rectangular and the charge/discharge curves showed the ideal triangle shape. Similarly, Karthika et al.^[^ ^229^ ^]^ used phosphoric acid as an activating and doping agent for the synthesis of phosphorus‐doped graphene. This activated P‐doped graphene electrode, however, exhibited a gravimetric capacitance of 367 F g^−1^, showing much better performance as compared to that of the undoped graphene. Theoretical calculations predicted that phosphorous could be either adsorbed on the graphene sheets or could be substitutionally doped in the graphene lattice.^[^ ^230^ ^]^ From the results, it was found there could be two plausible mechanisms to explain the enhancement of the overall electrochemical performance. First, the inter‐layer distance increases due to phosphorus doping so that the electrolyte ions can freely move between the layers. Second, doping‐induced P‐O functional groups may provide additional pseudo‐capacitance, enhancing performance. It was also observed that the P atoms preferred adsorption rather than substitution in the graphene layers. Several phosphorous precursors such as phosphate salts, phytic and phosphoric acids have been used to dope graphene for supercapacitor applications^[^ ^231^ ^]^ and all the doped graphene exhibited enhanced capacitance.

As discussed in the earlier section, co‐doping can tune the electronic properties of graphene and enhance electrochemical performance by providing more active catalytic sites. Therefore, the electrochemical properties of graphene have also been investigated by codoping with nitrogen and phosphorus. In a hydrothermal‐based synthesis, GO was treated with melamine phosphate followed by freeze‐drying and heat treatment to generate N, P‐co‐doped graphene monoliths containing 6.48 at% of N and 3.0 at% of P with a surface area of 280 m^2^ g^−1^.^[^ ^232^ ^]^ These monoliths showed a specific capacitance of 213 F g^−1^ at 50 mA g^−1^, whereas the powder form showed a specific capacitance of 183 F g^−1^ at the same density. Notably, these electrodes are highly stable even at high operating potentials and after 10 000 cycles. Even higher specific capacitance was achieved in N, P‐co‐doped mesoporous graphene synthesized using a soft‐templated method by Zhang et al. The supercapacitor made by this electrode exhibited a specific capacitance of 245 F g^−1^ at 0.5 Ag^−1^ and only 5% loss after 3000 cycles at 5 Ag^−1^.^[^ ^233^ ^]^ The high specific capacitance is mainly attributed to N and P doping‐induced more active sites and enhanced conductivity. In addition, the mesoporous structure also provides more space for energy storage. Likewise, Zhao and colleagues adopted a hydrothermal method to synthesize N,P‐codoped graphene, which presented a high specific capacitance of 306 F g^−1^ at 0.3 A g^−1^ in ZnSO_4_ solution and was used as the cathode material for the zinc‐ion hybrid supercapacitor.^[^ ^234^ ^]^ Such a high capacitance was ascribed to the porous structure, large specific surface area, as well as N/P ratio. As a result, the as‐obtained optimized zinc‐ion hybrid supercapacitors delivered a large specific capacitance of 210.2 F g^−1^, a high energy density of 94.6 Wh kg^−1^, as well as good stability with 82% of initial capacitance after 15 000 cycles.

In another report, GO was first treated with ammonia solution and then dispersed in a polymerized phosphorus‐containing gel, followed by annealing at a high temperature. The co‐doped graphene showed a good gravimetric capacitance of 192 F g^−1^ compared to 112 F g^−1^ for the single‐element N‐doped graphene. In the same year, Nazarian‐Samani et al. reported the supercapacitor application of the N, P codoped graphene synthesized by thermally treating phosphoric acid and a nitrogen‐containing source with glucose.^[^ ^235^ ^]^ It was observed that sp^2^ bonds converted to sp^3^ bonds at high temperatures (i.e., 1000 °C), whereas there was no change at 400 °C. Therefore, the codoped graphene exhibited better performance with a specific capacitance of 235.5 F g^−1^ at 0.5 A g^−1^ at a relatively low temperature. Wu et al. analyzed the effect of surface functional groups on N, P‐co‐doped graphene and its electrochemical performance.^[^ ^236^ ^]^ The investigation indicated that graphene oxides tended to have hydroxyl and carboxyl functional groups. These functional groups strongly influenced the doping in the graphene lattice as well as the textural properties. GO possessed high content of the oxygen functional groups, which led to a low active surface area. Therefore, reducing the GO with (NH4)_3_PO_4_ and doping N and P simultaneously enhanced its electrochemical properties with an enhanced specific capacitance of 219 F g^−1^ and energy density of 8.2 Wh kg^−1^. Following their earlier work, Wu et al. prepared nanohybrids of carbon‐coated graphene doped with nitrogen and phosphorus using phytic acid and amine for supercapacitor applications.^[^ ^237^ ^]^ The prepared microstructure possessed micro/mesoporous structure, high surface area, and N and P codoping with a doping concentration of 3.6 and 0.3 at% respectively. The specific capacitance obtained is about 201 F g^−1^ with an energy density of 9.1 Wh Kg^−1^. Recently, a supramolecular approach was used to dope graphene using phytic acid and melamine as the precursors. The mesoporous graphene doped with N and P yielded a high gravimetric capacitance as high as 416 F g^−1^.^[^ ^238^ ^]^ A commercial‐level mass production of ternary atom (B, N, P) doped graphene was achieved by Zhang et al. using a hydrothermal method.^[^ ^239^ ^]^ The flexible supercapacitor devices fabricated based on these electrodes showed a specific capacitance of 350 F g^−1^ with an operating voltage of 0.3 V.

From the above discussion, it is clear that pyridinic heteroatom (B, N, P, S) doping creates electro‐chemically active sites and induces pseudo‐capacitance behavior. Better electron injection helps, so N/S‐doped graphene could perform better than B‐doped graphene systems. In addition, co‐doping of heteroatoms, e.g., S‐N or P‐S, or the incorporation of specialized polymers with electron‐donating properties leads to enhanced electrochemical performance. Other strategies, like the activation/functionalization of doped graphene systems, will also provide electrochemically active sites. Most importantly, inducing well‐organized porosity in carbon‐based electrode materials can further enhance their supercapacitive performance due to enhanced access to electrochemical sites.

### 3.5 Fullerenes‐Based Supercapacitors

**Figure 7:**
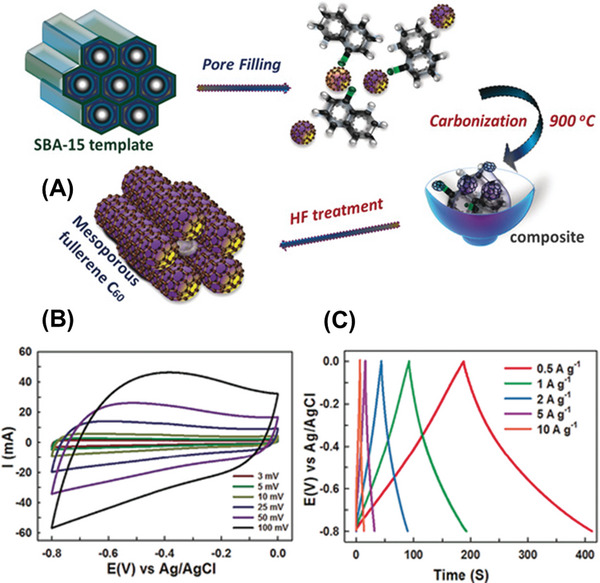
A) Schematic representation of the synthesis of mesoporous fullerene, C_60._ B) Cyclic voltammograms of supercapacitor using mesoporous C_60_ as electrodes measured at different scan rates. C) Galvanostatic charge‐discharge measurements of the supercapacitor measured at different current densities. Reproduced with permission.^[^ ^244^ ^]^ Copyright 2017, Wiley‐VCH.

Fullerenes were first discovered by Smalley, Kroto, and Kurl in 1985.^[^ ^67^ ^]^ Fullerenes possess cage‐like structural morphology with 60 or 70 carbon atoms in the case of C_60_ and C_70,_ respectively. Since their discovery, fullerenes have been extensively used in the field of solar cells and biology as electron transport materials, antimicrobial and antiviral agents due to their excellent photo‐physical properties, unique structure, and *π*‐conjugation.^[^ ^240^ ^]^ Fullerenes have been used as electrodes for supercapacitors for a long time, whereas the electrical conductivity and surface area of fullerene are relatively low, resulting in low capacitance.^[^ ^241^ ^]^ The low capacitance comes from the formation of EDL with the electrolyte. For example, the capacitance achieved from the electrode using rod‐shaped fullerenes derived from the crystallization of m‐xylene is around 6 F g^−1^.^[^ ^242^ ^]^ To achieve high‐performance supercapacitors, Sun et al. synthesized microporous conjugated C_60_ films exhibiting a specific capacitance of 117 F cm^−3^, confirming the importance of the high surface area for supercapacitors.^[^ ^243^ ^]^ Vinu et al. reported mesoporous C_60_ synthesized by hard template method using SBA‐15 as the template.^[^ ^244^ ^]^ A schematic representation of the synthesis methodology is depicted in **Figure** **7**A. Figure 7B shows the cyclic voltammograms measured at different scan rates. The CV curves showed a quasi‐rectangular feature resembling that of an ideal capacitor. The resulting mesoporous C_60_ had a rod‐shaped morphology with a high surface area (680 m^2^ g^−1^) and ordered porosity yielding a specific capacitance of 141 F g^−1^ at 0.5 A g^‐1^ (Figure 7C). While, mesoporous C_70_ synthesized by a similar method exhibited a lower surface area of 454 m^2^ g^−1^, but exhibited a higher specific capacitance compared to that of the mesoporous C_60_ prepared under similar conditions.^[^ ^245^ ^]^ It exhibited a specific capacitance of 172 F g^−1^ at 0.5 A g^−1^ and good stability at higher current densities. The increase in capacitance is due to the better crystallinity of mesoporous C_70_ compared to that of mesoporous C_60_. In both materials, the enhancement of the specific capacitance arises from the mesoporous nature of the fullerene, which allows the electrolyte ions to move freely between the electrodes. Activation is another approach to introduce micro or mesopores in fullerenes, which leads to capacitance enhancement. Zheng et al. used KOH to activate C_70_ microtubes to obtain a specific capacitance of about 322 F g^−1^ at 0.1 A g^−1^ with excellent stability.^[^ ^246^ ^]^ The activation introduced macro and micropores, high density of surface oxygen functional groups, and graphitic carbon, which all contribute to the high performance of the supercapacitors. In 2021, Qiu's group first adopted covalent organic frameworks (COFs) as templates for synthesizing ordered nanoporous C60, demonstrating an energy density of 21.4 Wh kg^‐1^ at a power density of 900 W kg^‐1^ thanks to the improved electrochemical accessibility.^[^ ^247^ ^]^

In order to improve the capacitive behavior of fullerene, composites of fullerene formed with conducting polymers and metal oxides have been reported.^[^ ^248^, ^249^, ^250^ ^]^ For example, the composite formed by polyaniline and C_60_ whiskers showed a capacitance of 813 F g^−1^ at 1 A g^−1^ with high stability, retaining 85% even after 1500 cycles.^[^ ^251^ ^]^ Polyaniline has a theoretical specific capacitance of 2000 F g^−1^ because of its several redox states, but it is not stable at high current densities. Hence, carbon structures integrated with polyaniline to form a composite can utilize the high capacitance from polyaniline to enhance supercapacitance. In addition, the overall stability is strongly improved with the optimal composition. Very recently, Mohanty and co‐workers prepared an activated fullerene/zinc cobaltite (A‐C60‐ZCO) composite for supercapacitor applications.^[^ ^252^ ^]^ In this system, A‐C60 enhances stability and conductivity, while ZCO helps with charge storage, ensuring good specific capacitance of 593.2 F g^‐1^. Consequently, the derived flexible supercapacitor illustrated an energy density of 36.43 Wh kg^‐1^, a power density of 1681.47 W kg^‐1^, and 91.06% capacitance retention after 5000 cycles. Recently, Riaz et al. adopted a solvothermal method to synthesize potassium cadmium chloride (KCdCl_3_) based halide perovskite nanocomposites with C_60_.^[^ ^253^ ^]^ The prepared KCdCl_3_/C60‐based electrodes showed high specific capacitance of 1135 F g^‐1^ at 5 mV s^‐1^ and cyclic stability of 97.6% retention over 3000 cycles because the KCdCl_3_/C60‐based electrode provided more active sites for electrochemical response and facilitated the charge/ions movement pathway, making them suitable for use as next‐generation supercapacitor electrode materials. In 2023, Iqbal and co‐workers fabricated dual‐functional La_2_O_3_–C_60_ nanocomposites for photocatalysts and electrode material for supercapacitors.^[^ ^254^ ^]^ Thanks to the lower energy band gap, presence of deep‐level emissions, and lower recombination rate of photogenerated charge carriers compared to bare La_2_O_3_, the composites exhibited enhanced photocatalytic activity, as well as improved specific capacitance, outstanding energy density, and excellent stability for up to 5000 cycles in a 1 m KOH electrolyte.

Doping in fullerene can also enhance capacitance. For example, Winkler et al. synthesized the fullerene composite with C_60_ and palladium to achieve a specific capacitance of 300 F g^−1^.^[^ ^255^ ^]^ Co‐doping of nitrogen and Fe in fullerene could achieve a specific capacitance as large as 505 F g^−1^ at 0.1 A g^−1^ with good cyclic stability.^[^ ^43^ ^]^ In another work, Jiang and co‐workers manipulated fullerene self‐assembly with cobalt and nitrogen doping to prepare mesoporous carbon composites.^[^ ^256^ ^]^ Thanks to the confined state of the cobalt during the calcination, a highly homogeneous distribution of Co,N‐doping was obtained within the fullerene‐based carbon composites, leading to a significantly higher specific capacitance of 416.31 F g^−1^ at 1 A g^−1^ and excellent cyclic stability without activity loss after 5000 cycles.

Fullerene has lower electrical conductivity compared to the other two counterparts, which hampers its electronic current collection efficiency. In this regard, hybridization of fullerenes with graphene or CNTs becomes the most straightforward approach to enhance its performance, in which graphene/CNTs being good electronic conductors provide not only the efficient current collection but also extra electrochemically active sites. Similar to graphene, doping/co‐doping, activation, and porosity are other common strategies to enhance supercapacitive performances.

### 3.6 CNTs‐Based Supercapacitors

**Figure 8:**
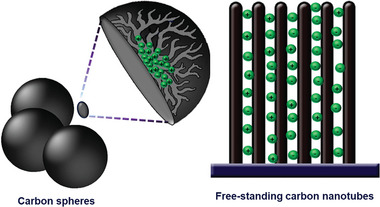
Schematic model comparing the ion diffusion for carbon spheres (left) with SWCNTs (right).

In 1991, Ijima discovered CNTs, another allotrope of carbon using an arc‐discharge method.^[^ ^75^ ^]^ After this discovery, several CNTs synthesis methods have been reported. CNTs have demonstrated incredible mechanical, thermal, and chemical stability, electrical conductivity, high surface area, large porosities, and optical properties. Their applications in the field of transistors are noteworthy, and they have also been highly used as field‐emitting diodes. The high surface area, unique porosities, inertness toward chemicals, and good electrical conductivity of CNTs make them suitable electrode materials for supercapacitors.^[^ ^257^, ^258^, ^259^, ^260^ ^]^ Notably, CNTs show better catalytic activity compared to fullerenes and activated carbons which are known to have relatively high surface area. The high catalytic activity of CNTs is generally assigned to large accessible spaces for the electrolyte ions to move freely between the electrodes, leading to increased capacitance. In contrast, activated carbon usually possesses non‐uniform pores, which may produce resistance to the free motion of electrolyte ions in‐between the electrodes, resulting in relatively lower capacitance (**Figure** **8**).

The capacitive nature of the CNTs can be increased beyond the EDL through inducing pseudocapacitive behavior by adding functional groups or from the residual catalyst in fabricating CNTs. For example, CNTs prepared by the CVD method showed a specific capacitance of 102 F g^−1^ in acidic media with a power density of 8000 W Kg^−1^.^[^ ^128^ ^]^ Flexible CNTs film developed on polydimethylsiloxane (PDMS) showed a specific capacitance of 54 F g^−1^ in an organic electrolyte.^[^ ^160^ ^]^ PDMS substrate creates a wave geometry of the CNT films and also induces hydrophilic groups when exposed to ultra‐violet light that increases the wettability and the overall specific capacitance. Compared to other carbon electrodes, CNTs showed an areal capacitance from 0.8 to 280 mF cm^−2^, which is far superior to those of the activated carbons (0.4 to 3.1 mF cm^−2^).^[^ ^261^ ^]^ Free‐standing supercapacitor electrodes made from CNTs showed a very high energy density (1.5 mWh cm^−3^) and power density (4.2 W cm^−3^) with a volumetric capacitance of 3.0 F cm^−3^.^[^ ^262^ ^]^ While a similar report showed that an areal capacitance of 601 µF cm^−2^ with excellent stability even at a high current density of 6400 A g^−1^ was observed for a supercapacitor made from CNTs and it retained 98% of the capacitance after one million cycles.^[^ ^263^ ^]^ The increase in capacitance and excellent ac‐line filtering ability is because of the low series resistance in the CNT films. Densely packed SWCNTs (density = 0.03 g cm^−3^) showed a specific capacitance of 80 F g^−1^. The capacitance of the SWCNTs is estimated to be 20 F g^−1^ from the discharge curves of the cells charged at 2.5 V in a two‐electrode cell, corresponding to 80 F g^−1^ for a three‐electrode cell (tetraethylammonium tetrafluoroborate (Et_4_NBF_4_)/PC electrolyte). The rate performance of SWNT solid and SWNT forest cells are better when compared to the activated graphene. The increase in specific capacitance is due to porosity, which facilitates electrolyte ion diffusion.

Functionalization is one efficient strategy to enhance the supercapacitor performance of CNT since it can enhance pseudo‐capacitance. CNTs functionalized with different amounts of oxygen functional groups showed a specific capacitance ranging from 24 to 58 F g^−1^ in organic electrolytes.^[^ ^264^ ^]^ Even though there was no significant change in the surface area due to functionalization, the electrolyte ions interacted differently with the electrodes with different levels of oxygen functionalities. The high content of the oxygen functional groups may increase the pseudocapacitive behavior and reduce the overall capacitance. Therefore, having the right oxygen content will avoid the excess charge transfer between the electrode and electrolyte, enhancing the double‐layer capacitance. Kim et al.^[^ ^265^ ^]^ demonstrated an increase in the capacitance of CNTs (8.3 F g^−1^ to 61.3 F g^−1^) by adding a redox molecule (decamethylferrocene) to an organic electrolyte. It was observed that after the addition of decamethylferrocene, the capacitance increased 27 times with an enhanced energy density of 36.8 Wh kg^−1^ and a high operation voltage of 2.1 V.

Another strategy to improve supercapacitor performance is to form composites with other materials. The unique mix of pseudocapacitive materials, including transition metal oxides, metal sulfides, metaloxy hydroxides, carbon nitrides, metal nitrides and conducting polymers, and carbon nanomaterials in one composite could considerably improve the electrochemical performance by combining the positive synergism among each component including continuous and fast reversible redox reactions at the surface of pseudocapacitive materials, and charging‐discharging at the high surface area electrode and electrolyte interface while overcoming the drawbacks of each material. In this term, Zhao et al. reported that CNTs composites formed with two different conducting polymers (polypyrrole and poly(3‐methylthiophene)) achieved a specific capacitance of 72 and 87 F g^−1^, respectively.^[^ ^266^ ^]^ Moreover, the composite film based on SWCNTs and polyaniline prepared by electrodeposition showed a specific capacitance as large as 380 F g^−1^ and an areal capacitance of 1147.12 mF cm^−2^ as well as a high energy density of 50.98 µWh cm^−2^.^[^ ^267^ ^]^ The enhancement of capacitance could also be achieved using electrospinning to fabricate polyaniline/CNT fibers, which showed a high specific capacitance of 386 F g^−1^ compared to that of the normal CNT/polyaniline composite (308 F g^−1^).^[^ ^268^ ^]^ The interconnection of nanofibers with each other benefits the electrical conductivity and the generation of porosities during the spinning of nanofibers facilitates the free flow of the electrolyte ions. Besides the high capacitance, the supercapacitor was able to withstand 200% of omnidirectional strain without any capacitance loss.

The composite formed by CNT and polymers exhibited excellent stability. For instance, the film formed by the electrodeposition of polypyrrole and CNTs showed high stability in a harsh environment, possessed a capacitance of 4.9 F cm^−3^ and retained 95% of its initial capacitance even after 10 000 cycles.^[^ ^269^ ^]^ Beyond that, conducting polymer/CNT‐based asymmetric supercapacitor possessed a very high operational voltage of 4 V in organic electrolyte with high energy and power densities.^[^ ^270^ ^]^ The areal capacitance is in the range of 0.19–0.70 F cm^−2^, and the high capacitance originates from the pseudocapacitive behavior of the poly(3,4‐ethylenedioxythiophene) (PEDOT). In addition, MWCNTs treated with 25 wt% polyaniline showed a specific capacitance as high as 497 F g^−1^.^[^ ^271^ ^]^ From the above discussion, it was clear that the composite showed better cyclic stability and capacitance retention when compared to pure polyaniline or pure CNTs. Therefore, forming a composite with CNTs and polymers has effectively achieved high specific capacitance, areal density, excellent stability, and capacitance retention. Moreover, the polymer can provide gel properties to fabricate flexible supercapacitors. For example, a symmetric flexible supercapacitor made from the hydrogel of CNTs/polyaniline as the electrodes showed a specific capacitance of 315 F g^−1^.^[^ ^272^ ^]^ These flexible electrodes showed very low capacitance loss during 150 bending cycles at an angle of 180^o^. The charge storage could be visibly observed in these electrodes by means of reversible chromatic shifts when a potential is applied.

Besides polymers, transition metal oxides from zinc, nickel, manganese, and ruthenium have also been used to fabricate CNTs‐based composites. For instance, Chandra's group designed efficient asymmetric supercapacitors for aqueous systems using MnO_2_ and MoS_2_ thin film heterostructures on free‐standing CNTs, which shows high areal capacitance of 0.6 and 0.41 F cm^−2^ for MnO_2_/CNT and MoS_2_/CNT, respectively.^[^ ^273^ ^]^ Further studies revealed that the combination of chemical and physical deposition techniques created a hierarchical core‐shell heterostructure, offering high supercapacitor performance thanks to their hydrophilic electrodes and minimal resistive losses. In another example, a stratified flower‐like composite of NiO/MnO_2_/CNTs was synthesized as supercapacitor electrodes, which exhibited a high specific capacitance of 1320 F g^‐1^ at 1 A g^−1^, along with maintaining more than 90% of its initial capacitance after 3000 cycles.^[^ ^274^ ^]^ The composite electrode combines the advantages of pseudocapacitors and EDLCs while addressing the drawbacks of each component. The synergistic interactions of MnO_2_, NiO, and CNT materials with an optimal mass ratio result in excellent specific capacitance, hinting that binary metal oxides with composites of pseudo‐material and carbon material show promising potential for supercapacitor applications.

Very recently, Pan and co‐workers fabricated a flexible quasi‐solid‐state asymmetric supercapacitor composed of a self‐assembled MXene/MoO_3_ (negative electrode with high capacity and cycle stability) and interpenetrating A‐CNTs/K*_x_* MnO_2_ (positive electrode with improved rate capability and capacitance).^[^ ^275^ ^]^ The as‐prepared supercapacitor showed a high capacitance of 65.5 F g^−1^, energy density of 36.5 Wh kg^−1^, and cycle stability with a retention of 91.7% after 6000 cycles. To enhance the area and provide access to electrochemically active sites, CNTs can be cast into aerogels. CNTs aerogels‐based thermo‐electrochemical cells for converting low‐grade waste heat into electricity have been realized.^[^ ^276^ ^]^ When cast into aerogels, hybrids of MWCNT and Ruthenium hydroxide exhibited very high specific capacitance of ≈423 F g^‐1^ @ 5 mV s^‐1^.^[^ ^277^ ^]^ N/S co‐doped carbon nanotube aerogels exhibited high specific capacitance ≈328 F g^‐1^ (@1 A g^‐1^), rate capability ≈66.5% (1–10 A g^‐1^), energy density ≈45.6 Wh kg^−1^ (@ 0.5 kW kg^−1^) and importantly the capacity retention as high as ≈97.4% (even after 10k cycles).^[^ ^278^ ^]^ From these examples, the fabricated asymmetric supercapacitors generally exhibited high capacitance, good mechanical stability, and high operational voltage, providing a straightforward strategy for developing next‐generation supercapacitors with high energy and power densities.^[^ ^279^, ^280^, ^281^, ^282^ ^]^

It is well acknowledged that carbon nanomaterials, including graphene, CNTs, and fullerene, have demonstrated initial but promising results for energy storage applications thanks to their excellent electronic conductivity with high charge transport mobilities. The outstanding specific surface areas of these unique materials empower them for double‐layer ion adsorption/desorption. Reliable supercapacitor electrodes have been fabricated based on these materials, and different modification techniques, including doping, thickness *π*–*π* stacking interactions, and edge functionalities, have been employed to optimize the performance. Their excellent thermal conductivities render them fireproof materials when integrated into supercapacitor devices and under high load/rate functionalities. Additionally, combining with conductive polymers or metal oxide to introduce pseudo‐capacitance has been attempted extensively. Despite the great potential, the research of these materials is just in its nascent stage, and many fundamental questions still need to be addressed before their commercialization for practical supercapacitors. For instance, these carbon nanostructures tend to aggregate, which will severally decrease their specific surface areas and performance. Also, compared with mesoporous carbon and activated carbon, large‐scale synthesis of these nanomaterials requires high‐cost and is time‐consuming. Therefore, more practical synthetic approaches must be developed for future practical applications.

## 4 Lithium‐Ion Battery

### 4.1 Introduction to Lithium‐Ion Battery

Lithium‐ion battery (LIBs) is one of the most successful technologies among commercialized energy storage devices due to their excellent volumetric and gravimetric energy densities, low self‐discharging characteristics, high stability, and long‐term durability.^[^ ^283^ ^]^ The superiority of LIBs for energy storage can be gauged by their uses in a wide range of portable electronic gadgets.^[^ ^284^ ^]^ However, the practical energy storage capacity of conventional LIBs is still far behind the current demands for medium/large electric vehicles due to their insufficient energy density to be comparable to fuel combustion. Therefore, it is of paramount importance to design new electrode materials for LIBs with precisely‐engineered physicochemical characteristics that would help to realize their full potential in real‐time energy storage applications and meet higher energy density demands.^[^ ^24^, ^285^ ^]^

**Figure 9:**
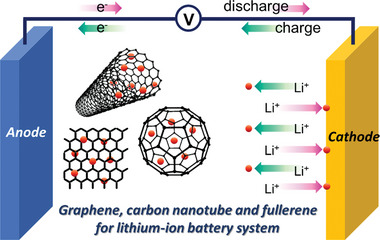
Schematic illustration of LIB system using graphene, CNT, and fullerene as an anode electrode.

Over the past decades, massive attempts have been made to investigate carbonaceous electrode materials for LIBs which allowed for the considerable exploration of diverse carbon allotropes, including graphene, CNTs, and fullerene, for their energy storage behaviors in LIB systems (**Figure** **9**).^[^ ^286^, ^287^ ^]^ However, inherent limitations of these carbonaceous materials must be addressed urgently, such as insufficient binding sites for Li^+^ ion adsorption, uncontrollable solid‐electrolyte interphase (SEI) layer and volume variation depending on lithiation/delithiation process. In order to overcome these problems and markedly enhance the electrochemical functionalities toward Li^+^ ion storage, plenty of strategies for manipulating the physicochemical properties of fullerene, CNTs, and graphene have been explored. In this section, we overview not only the physicochemical properties of graphene, CNT, and fullerene, which primarily determine the electrochemical functionalities but also the strategic ways for manipulating these physicochemical properties to elicit LIBs behaviors toward the positive direction.

### 4.2 Graphene for Li‐Ion Storage

It has been reported that graphite, a conventional anode material for commercialized LIBs systems, forms LiC_6_ configuration by intercalating the Li^+^ ions, yielding a theoretical capacity of 372 mAh g^−1^. This value, however, is still far below the energy density required for medium/large electric vehicles and large‐scale renewable energy storage. In this context, as single layer form of graphite, graphene has been receiving tremendous attention owing to its high intrinsic carrier mobility (350,000 cm^2^ V^−1^ s^−1^), excellent thermal conductivity (≈3000 W m^−1^ K^−1^), high theoretical specific surface area (2,630 m^2^ g^−1^), superior mechanical strength, high flexibility, and high Li^+^ ion diffusivity (10^−7^−10^−6^ cm^2^ s^−1^).^[^ ^288^, ^289^ ^]^ According to a number of computational simulations, it has been predicted that graphene could possess a wide range of active spaces for Li^+^ ion accommodation, which are available at the edges, grain boundaries, defects, as well as both sides of graphene sheets.^[^ ^290^ ^]^

#### 4.2.1 Structural Modification

##### Size

It is well acknowledged that small particle‐sized materials generally deliver better specific capacity and cyclability than their bulk counterparts because of largely suppressed particle cracking and shortened Li^+^ ion diffusion path during electrochemical cycling.^[^ ^291^ ^]^ Gerouki et al. reported the density of states (DOS) calculations for single‐layer graphene nanosheet (SLG NS) with small diameters, which indicate the lithium storage capability would be facilitated by decreasing the diameter of single graphene NSs.^[^ ^292^ ^]^ The specific capacity of SLG NS with 0.7 nm diameter was estimated to be 1,488 mAh g^−1^ under the expectation of Li_4_C_6_ formation. The physicochemical approaches have been employed to effectively control the size of graphene NSs, such as sonochemistry, nanocutting, and controlled etching.^[^ ^293^, ^294^, ^295^ ^]^ For example, Guo et al. synthesized crumpled‐paper‐shaped graphene NSs with a small crystallite size of 9.1 nm upon the ultrasonic process.^[^ ^296^ ^]^ The presented material delivered a specific capacity of 650 mAh g^−1^, which is higher than artificial graphite. In addition, Wang et al. prepared laterally confined graphene sheets with sizes of 100–200 nm by using the arc‐discharge evaporation method under a mixed NH_3_−He atmosphere.^[^ ^297^ ^]^ These laterally confined graphene sheets delivered better electrochemical properties than those of the graphene sheets with micro size, which could be attributed to the shortening of the transport path, large physical contact with electrolyte and exposure of many edges upon reducing the crystallite size.

##### Number of Layers

**Figure 10:**
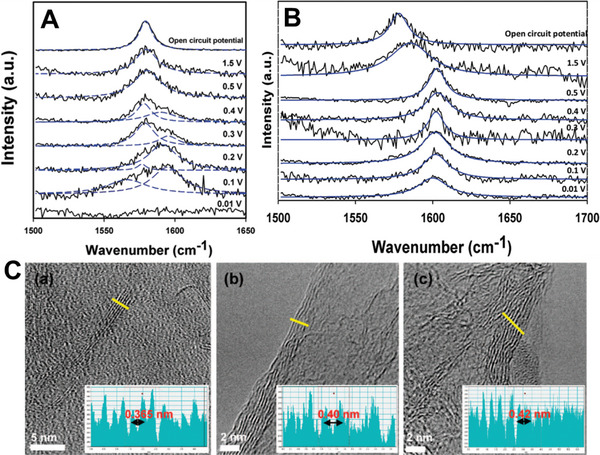
In situ Raman spectra of A) FLG and B) SLG at selected potentials during lithiation (dash line represents Lorentzian fit). Reproduced with permission.^[^ ^298^ ^]^ Copyright 2010, American Chemical Society. C) Cross‐sectional TEM images of GNS families with almost the same numbers (5−6) of graphene stacking layers for (a) GNS, (b) GNS+CNT, and (c) GNS+C_60_. Reproduced with permission.^[^ ^306^ ^]^ Copyright 2008, American Chemical Society.

In addition to the enormous scientific interest in SLG NS due to its ability to adsorb the Li^+^ ions on both sides, many scientists also have paid attention to the lithium storage mechanism of a few layers of graphene (FLG) NS owing to their superior performance toward Li^+^ ion intercalation to bulk graphite. Many theoretical studies have been conducted to figure out the fundamental differences in terms of Li^+^ ion storage mechanism on FLG NS as compared to SLG and bulk graphite.^[^ ^298^, ^299^, ^300^ ^]^ Pollak et al. revealed fundamentally similar C—Li interaction of FLG NS to bulk graphite while SLG behaves completely differently, as proved by cyclic voltammetry (CV) and in situ Raman spectroscopy.^[^ ^298^ ^]^ As shown in **Figure** **10**A, in addition to the G‐band peak of graphite at 1582 cm^−1^, the appearance of a new shoulder peak at 1592 cm^−1^ was observed at 0.4 V as for FLG, which could be indexed as Li^+^ ion‐intercalated into interior graphene layer. While further lithiation caused the increase of the interior peak, the exterior peak where Li^+^ ions were not inserted inside became depressed. In addition, at the final stage of lithiation at 0.01 V, both of the peaks totally disappeared due to the interference of the Raman‐active E_2g2_ mode by lithium intercalation. As quite different from FLG, shift of G‐band peak was observed only up to 0.5 V and further lithiation did not induce any change, indicating the maximum level of Li^+^ ion intercalation at 0.5 V (Figure 10B). The absorption of Li^+^ ions on both sides of SLG NS was significantly restricted by the lower binding energy of Li—C interaction and strong repulsion forces between Li^+^ ions.

Recently, Ji et al. clarified that there would be no difference in intercalation mechanism between bilayer or FLG NSs and bulk graphite and proposed a planar Li storage scheme with the four‐time fractal way of Li intercalation toward LiC_6_ formation.^[^ ^301^ ^]^ They also clarified the fact that the Li^+^ ions could be stored only by means of intercalation into graphene interlayers. These observations gradually triggered increased interest in FLG NSs for LIBs application. The number of graphene layers significantly affects the electronic band structures, so the scientific strategies to manipulate the number of graphene NSs have received prime attention.^[^ ^302^, ^303^, ^304^ ^]^ Tong et al. controlled the number of graphene layers by a series of steps, including oxidation of graphite, expansion of graphite oxide, and subsequent reduction.^[^ ^304^ ^]^ The authors obtained the graphene sheets with single, triple, and quintuplicate layers, exhibiting a specific capacity of 846, 730, and 628 mAh g^−1^ after 20 cycles, respectively, suggesting that the number of graphene layers could have an influence on the electrochemical capability for Li^+^ ion accommodation. Furthermore, Lian et al. synthesized four layers‐stacked graphene NSs with a thickness of 2.1 nm via the thermal exfoliation method. The present graphene NSs maintained the specific capacity of 848 mAh g^−1^ at a current density of 0.1 A g^−1^ after 40 cycles.^[^ ^305^ ^]^

##### Interlayer Distance

The adoption of graphene sheets can increase the theoretical specific capacity of LIBs to 744 mAh g^−1^ as lithium ions can be captured on both sides of the graphene sheet through the formation of LiC_3_.^[^ ^290^ ^]^ However, narrow interlayer distance in multi‐layered graphene restricts the mobility of Li^+^ ions on the active sites of anode material, which may result in poor specific capacity for graphene‐based electrode materials. A plausible solution to overcome this problem would be coupling graphene with another carbon‐rich material, which may increase the interlayer separation distance. For example, graphene NSs (GNSs) with an enlarged interlayer distance were prepared by incorporating CNT and C_60_, which were named as GNS+CNT and GNS+C_60_, respectively. As shown in Figure 10C, the high‐resolution transmission electron microscopy (HRTEM) analysis revealed that the interlayer distances of the GNS (0.365 nm) were enlarged to 0.40 nm and 0.42 nm upon the incorporation of CNTs and C_60_, respectively. When applied to the LIBs system, both interlayer‐enlarged GNSs delivered 730 and 784 mAh g^−1^ for GNS+CNT and GNS+C_60_ at a current density of 0.05 A g^−1^ while GNS itself showed a reversible capacity of 540 mAh g^−1^.^[^ ^306^ ^]^ The observed improvement in LIBs functionality of GNS could be ascribed not only to the improvement of electrical conductivity but also to the enlarged interlayer spacing, which could provide additional sites for Li^+^ ion storage. Also, Cai et al. prepared GNSs with an increased interlayer distance of 0.385 nm through the combination of the freeze‐drying process and thermal reduction.^[^ ^307^ ^]^ The present material exhibited quite promising battery functionality of the specific capacity of ≈600 mAh g^−1^ at a current density of 1 A g^−1^, which could be attributed to the large accommodation of Li^+^ ions in the basal plane of graphene NSs caused by expanded interlayer distance as well as high surface area.

##### Porous Morphology

**Figure 11:**
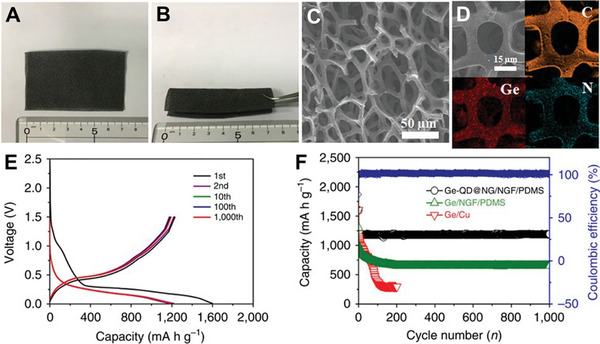
A,B) Images of flexible Ge‐QD@NG/NGF electrode with 7 × 4 cm. C) SEM and D) EDS‐mappings of Ge‐QD@NG/NGF structure. E) Voltage profiles at various cycling stages and F) cycling behavior at 1C of Ge‐QD@NG/NGF structure. Reproduced with permission.^[^ ^311^ ^]^ Copyright 2017, Springer Nature.

Graphene NSs have been regarded as an outstanding carbon framework with high specific surface area, mechanical robustness, and chemical/thermal stabilities. However, their severe self‐stacking tendency by strong *π*–*π* interaction leads to a depressed surface area and concomitantly poor electrode–electrolyte contacts and high interfacial resistance, resulting in significant suppression of electrochemical functionality. Therefore, constructing the graphene architecture with high porosity allows not only the large penetration of the electrolyte with active material but also the provision of abundant active sites for Li^+^ ion storage.^[^ ^308^, ^309^ ^]^ Especially, porous graphene structures with 3D interconnected networks, such as graphene hydrogels, graphene aerogels, or graphene foams, possess large specific surface areas, multidimensional continuous electron‐transport pathways, and rapid ion‐diffusion characteristics as well as excellent mechanical strength.^[^ ^33^, ^310^, ^311^ ^]^ Mo et al. decorated Ge quantum dot/nitrogen‐doped graphene yolk shell on 3D interconnected porous nitrogen‐doped graphene foam (Ge‐QD@NG/NGF).^[^ ^311^ ^]^ As shown in **Figure** **11**A–D, the high flexibility and bending with clear 3D interconnected porous morphology could be observed even after the incorporation of active material on the surface of graphene foam. Utilization of the graphene foam as a matrix could significantly enhance battery performance compared to that without graphene foam (Figure 11E,F). The enhancement can be attributed to the provision of more space to accommodate charges and load electrode materials as well as provide a conductive pathway.

Porous graphene structure could be achieved by means of treatments with acid/oxidizer/base, hard templating, deposition, hydrothermal, and self‐assembly methods.^[^ ^312^, ^313^, ^314^ ^]^ The graphene NSs‐based 3D porous architectures were synthesized by Gong et al., which could be used as an efficient framework for metal compound additives.^[^ ^315^ ^]^ Benefiting from their large surface area and highly porous structure, the present 3D porous architecture could possess resistance to volume expansion, facile contact with electrolytes, and fast electron diffusivity. Ren et al. synthesized 3D hierarchical porous graphene aerogel with fine‐controlling of mesoporosity via hydrothermal self‐assembly and following in situ carbonization processes.^[^ ^316^ ^]^ The surface area of 383.7 m^2^ g^−1^ was observed for the 3D hierarchical graphene aerogel with a pore size distribution of 2–3 nm. According to the electrochemical test for Li^+^ ion storage, the specific capacity of ≈1100 mAh g^−1^ was maintained after 100 cycles at a current density of 0.1 A g^−1^, which could be due to the porous network providing the numerous Li^+^ ion adsorption sites and facilitating the Li^+^ ion diffusion kinetics. In another case, Wang et al. fabricated porous fiber‐shaped materials with a diameter of 1–1.5 µm and a length of 8–15 µm, which possessed a surface area of 821.9 m^2^ g^−1^.^[^ ^317^ ^]^ The presented materials maintained a reversible capacity of 837 mAh g^−1^ at a current density of 0.25 A g^−1^ after 150 cycles, which can be ascribed to the large surface area to accommodate plenty of Li^+^ ions, facile contact with electrolyte thanks to their morphological benefit. Furthermore, Sui et al. developed N‐doped graphene NSs with hierarchical porosity through freeze‐drying and subsequent calcination procedures, possessing a surface area of 1170 m^2^ g^−1^.^[^ ^318^ ^]^ The presented material achieved a specific capacity of 496 mAh g^−1^ at a current density of 0.4 A g^−1^, which benefited from highly enhanced physical contact with electrolyte.

#### 4.2.2 Creating Defect Sites

While the electrochemical performance of graphene NSs appeared to reach ≈460 mAh g^−1^ for Li^+^ ion accommodation,^[^ ^319^ ^]^ introducing defects on the graphene NSs gave further rise to the significant enhancement in LIBs delivering the 2−3 times larger specific capacity.^[^ ^320^ ^]^ For instance, Pan et al. synthesized highly‐disordered graphene NSs, which exhibited an impressive specific capacity of ≈800 mAh g^−1^ at a current density of 0.05 A g^−1^ after 15 cycles.^[^ ^320^ ^]^ The observed high capacity could be attributed to the provision of additional active sites for Li^+^ ion storage by edges and defects. As theoretical evidence supporting these enhancements of LIB functionality for defective graphene NSs, Lee and Persson conducted the DFT calculations indicating that the Li^+^ ions could not be intercalated into the defect‐free graphene, which would exhibit lower theoretical capacity than that of the bulk graphite.^[^ ^321^ ^]^ Also, Okamoto et al. reported the influence of carbon vacancy of graphene NSs on the adsorption energy of Li^+^ ions and they revealed that Li^+^ ions could be firmly trapped on the surface of graphene NSs with carbon vacancies, which can be ascribed to the presence of dangling bonds.^[^ ^322^ ^]^ The active defects, including vacancies, edges, and the following disorder degree, could offer plenty of active sites for Li^+^ ion storage, leading to the remarkable enhancement of specific capacity. In order to introduce defects on the surface of graphene NSs, many attempts have been made via doping heteroatoms, oxidation/reduction process, edge functionalization, and grain boundary, which give rise to defect‐enhanced Li adsorption and concomitantly improving the Li^+^ ion storage.

##### Heteroatom Substitution

**Figure 12:**
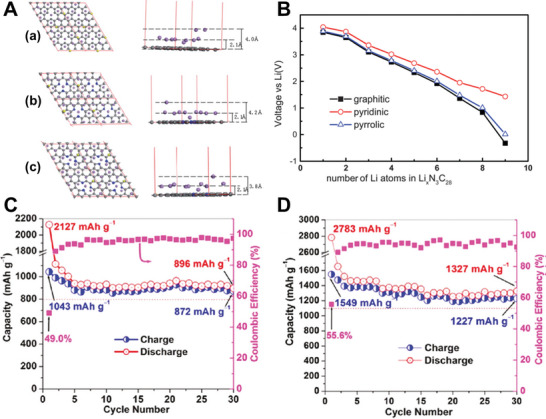
A) The stabilized locations of 10 Li atoms adsorbed on the surface of the three types of defective graphene NSs which is (a) graphene without N‐doping, (b) graphene with pyridinic‐N doping and (c) graphene with pyrrolic‐N doping; Left panel: normal direction view; Right panel: side view, where blue, gray and pink indicate N, C, and Li atoms, respectively. B) potential as a function of the number of intercalated Li atoms on the graphitic, pyridinic and pyrrolic defect structures of graphene. Reproduced with permission.^[^ ^335^ ^]^ Copyright 2012, Royal Society of Chemistry. Cyclability and QE of the C) NG and D) BG at current density of 0.05 A g^−1^. Reproduced with permission.^[^ ^341^ ^]^ Copyright 2011, American Chemical Society.

One of the effective approaches for introducing defects on the surface of graphene NS is the substitution strategy with heteroatoms which can improve the electrochemical, optical, textural, and micro/mesostructural properties and at the same time, introduce impurities into the carbon frameworks through surface/substitutional doping.^[^ ^323^, ^324^, ^325^ ^]^ Heteroatoms such as nitrogen, boron, sulfur, and phosphorous, fluorine (F), etc., have been widely explored in energy storage technologies, including supercapacitors and batteries for achieving improved electrode performances.^[^ ^326^, ^327^, ^328^ ^]^ It has been proved through both theoretical predictions and experimental results that the incorporation of heteroatom dopants in the graphene sheets can balance the lithium uptake and its diffusion leading to better electrical conductivity, mechanical robustness, and improved electrochemical performance.^[^ ^329^, ^330^, ^331^ ^]^ N is a ubiquitous choice for this purpose considering that its electronegativity (i.e., 3.5) is quite large compared to carbon (2.55), suggesting that N‐doped graphene (NG) can facilitate a higher uptake of Li^+^ ions.^[^ ^332^, ^333^ ^]^ Furthermore, the reversible discharge capacity of NG is almost 2 times higher than that of the pristine graphene, which is attributed to the presence of a higher number of defects on the active surface doped with nitrogen.^[^ ^334^ ^]^ Ma et al. performed theoretical calculations on the increase in reversible capacity using NG NSs.^[^ ^335^ ^]^ While the graphitic structure without N dopants possessed 8 Li atoms in the first layer, 9 Li atoms were stabilized on the inside of the carbon hexagon and defect sites of NGs with pyridinic and pyrrolic structures (**Figure** **12**A). According to the present result, the theoretical reversible capacity for these graphitic, pyridinic, and pyrrolic structures could be calculated as 1087, 1262, and 1198 mAh g^−1^, respectively, as shown in Figure 12B. Slightly higher values for pyridinic and pyrrolic structures than that of graphitic suggested that the presence of N‐containing moieties may help enhance the Li^+^ ion adsorption by acting as active binding sites. Ajayan et al. highlighted the importance of N‐doping for improving the performance of the LIBs by synthesizing NG materials grown on the copper current collector substrates using CVD.^[^ ^336^ ^]^ N‐doping increased the reversible discharge capacity of graphene from 0.03 to 0.05 mAh cm^−2^ when measured at a similar current density of 5 µA cm^−2^.

It is also well documented in the literature that the topological surface defects in the N‐doped graphene lead to the formation of disordered carbon structures resulting in the enhancement of Li intercalation. For example, NG was synthesized through a thermal reduction method using ammonium hydroxide to incorporate N into the graphene NSs.^[^ ^337^ ^]^ When used as an anode material in LIBs, the present NG showed excellent rate capability at a high current density of 10 A g^−1^ with long cyclic stability. Similarly, 3D NG synthesized via post‐thermal annealing in ammonia also exhibited a high specific capacity of 1094 mAh g^−1^ in the 100^th^ cycle at a current density of 0.2 A g^−1^.^[^ ^338^ ^]^ Thermal annealing with ammonia tends to form pyridinic N and pyrrolic N in the 3D graphene template, offering high electronic conductivity and structural stability for Li storage.

Apart from N atom, B is another interesting element that can be doped into the carbon framework to generate defect sites, imparting structural integrity with improved electrochemical performance.^[^ ^339^ ^]^ The electrochemical performance of B‐doped graphene (BG)‐based anodic electrode in LIBs can even surpass its nitrogen counterpart as demonstrated by Cheng et al.^[^ ^340^, ^341^ ^]^ The authors synthesized BG and NG by a simple heat treatment of pristine graphene with NH_3_ and BCl_3_ gas, respectively. As shown in Figure 12C,D, BG delivered a higher discharge capacity of 1327 mAh g^−1^ than NG (896 mAh g^−1^) at a current density of 0.05 A g^−1^ after 30 cycles. More interestingly, the materials retained their capacities at 199 mAh g^−1^ for NG and 235 mAh g^−1^ for BG even at a very high current density of 2.5 A g^−1^. This result strongly suggests that a proper choice of the heteroatom for graphene modification is crucial for determining the Li^+^ ion storage capacity of anodic electrodes in LIBs.

A dual heteroatom co‐doping approach with graphene has also been attempted to enhance the Li^+^ ion intercalation to improve its electrochemical performance.^[^ ^342^ ^]^ For instance, N and S co‐doped graphene exhibited a high initial discharge capacity of 1636 mAh g^−1^ with a Coulombic efficiency (QE) of 44.7%. The discharge capacity is two times higher than that of reduced graphene oxides (rGOs, 817 mAh g^−1^) and this material also showed excellent rate capabilities at a high current density of 5 A g^−1^ after 1500 cycles.^[^ ^343^ ^]^ P and N co‐doped porous graphene synthesized by the CVD method has also proved to be an efficient material for enhancing the electrochemical performance of anode. For example, P and N co‐doped anode material delivered a high reversible capacity of 2250 mAh g^−1^ at a current density of 0.05 A g^−1^ and rate capability of 750 mAh g^−1^ at 1 A g^−1^ without any capacity decay up to 1500 cycles.^[^ ^344^ ^]^ Similarly, N and F co‐doped graphene synthesized by hydrothermal treatment has also been explored as an anodic electrode material for LIBs.^[^ ^345^ ^]^ These materials possess unique structural properties, including a number of defect sites, enlarged interlayer space, and wrinkled open edge sites, which help achieve a superior reversible capacity of 1075 mAh g^−1^ at a current density of 0.1 A g^−1^ with good cyclic stability for up to 2000 cycles. The above‐mentioned examples illustrate that the incorporation of single or dual heteroatoms in graphene can be efficiently utilized to explore high‐performance anode electrodes for LIBs with good stability and without any appreciable loss in capacity.

##### Oxidation/Reduction Process

The processing conditions involved in the reduction of graphene oxide sheets play a crucial role in generating different types of defects. The most commonly used methods for reducing graphene include hydrazine, electron beam irradiation, and low‐temperature pyrolysis. To disclose the underlying mechanism of defect formation, Jiao et al. compared the specific capacity of rGOs prepared by using different synthetic methods.^[^ ^320^ ^]^ rGO sheets prepared through pyrolysis at a relatively low temperature of 300 °C and electron beam irradiation exhibited high reversible capacities of 1013 and 1054 mAh g^−1^, respectively, which were much higher than the pristine graphene (540 mAh g^−1^). A higher *I*_D_/*I*_G_ ratio in the Raman spectra of rGOs compared to the pristine graphene confirmed the presence of a larger number of defects in the rGOs, resulting in much higher lithium storage capacities. Shan et al. prepared surface defective graphene aerogel via the hydrothermal reaction of graphene oxide, maintaining a high reversible capacity of ≈800 mAh g^−1^ at a current density of 0.1 A g^−1^ after 100 cycles.^[^ ^346^ ^]^ The present battery performance could be attributed to the plenty of defects and vacancies together with the residual functional groups, facilitating the Li^+^ ion transfer property. However, as the introduction of many defects would offset the electrical conductivity, the optimization in the trade‐off between them should be addressed. Liu et al. reported rGO sheets with optimized defects and electrical conductivity by employing the microwave method, which displayed a high reversible capacity of ≈2000 and ≈900 mAh g^−1^ at a current density of 0.1 and 5 A g^−1^, respectively.^[^ ^347^ ^]^ The observed high battery performance could be ascribed not only to numerous active sites for Li^+^ ion accommodation but also to facilitating charge transfer kinetics by controlling the degree of surface defects. Mukherjee et al. prepared free‐standing graphene paper by means of the photothermal reduction of graphene oxide paper, obtaining a specific capacity of ≈370 mAh g^−1^ at a current density of 1.86 A g^−1^.^[^ ^348^ ^]^

##### Grain Boundary

**Figure 13:**
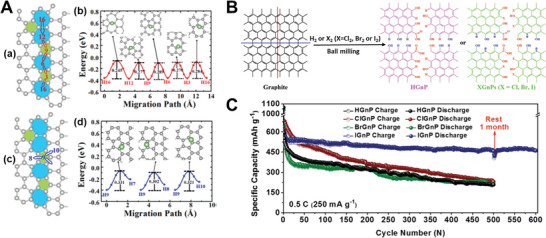
A) The schematic illustrations of diffusion path (a, c) and the energy evolutions (b, d) for the diffusion of a Li atom along the GB (a, b) and perpendicular to the GB (c, d). The insets indicate the local atomic structures of transition states. The values of energy barrier for the diffusion of a Li adatom are indicated. Reproduced with permission.^[^ ^354^ ^]^ Copyright 2014, American Chemical Society. B) A schematic illustration for edge‐selective functionalization of GnPs under H_2_, Cl_2_, Br_2_ or I_2_ to produce HGnP, ClGnP, BrGnP or IGnP. C) Cycling behavior at 0.5 C of HGnP, ClGnP, BrGnP, and IGnP in the voltage range of 0.02–3.0 V; the sequent cycling performance of the IGnP cell was obtained after 500 cycles and suspension for one month at ambient condition (25 °C). Reproduced with permission.^[^ ^363^ ^]^ Copyright 2014, Wiley‐VCH.

The introduction of grain boundary (GB) by fabricating the polycrystalline graphene structures could cause the defect sites close to the Fermi level.^[^ ^349^, ^350^ ^]^ The physicochemical properties, such as electronic, thermal, and mechanical properties of graphene NSs, could be finely modulated by GB, which affects the Li adsorption energy to form Li—C chemical bond.^[^ ^351^, ^352^, ^353^ ^]^ Some research groups proved that the GB markedly attracted the Li^+^ ions with favorable absorption energy for Li^+^ ions.^[^ ^354^, ^355^ ^]^ Furthermore, the diffusivity of Li^+^ ion through the GB of graphene NSs possessed a smaller energy barrier than that perpendicular to the boundary, indicating that the GB could play a pivotal role in opening the Li^+^ ion transport channel of graphene NSs during electrochemical cycling. For example, it was predicted that the Li^+^ ions could migrate along the GB with the energy barriers from 0.245 to 0.289 eV, which is much lower than the Li^+^ ions diffusion perpendicular to the GB ranging from 0.302 to 0.331 eV (**Figure** **13**A).^[^ ^354^ ^]^ This result clearly indicated that the Li^+^ ions would prefer to migrate along the GB rather than perpendicular to the GB and highlighted the significant role of GB as a Li^+^ ion migration channel.

#### 4.2.3 Edge Functionalization

Although Li^+^ ion diffusivity through the basal plane of graphene NSs is several orders of magnitude faster than that through the edge plane,^[^ ^356^, ^357^ ^]^ lithium diffusion at the edge plane of graphene NSs may still be facilitated via edge functionalization, which introduces diverse functional groups including hydroxyl and carboxyl groups.^[^ ^356^, ^357^, ^358^, ^359^ ^]^ Uthaisar *et al.* reported the graphene edge effect on the Li^+^ ion diffusivity according to DFT calculations, suggesting the fast discharge property for narrower graphene nanoribbons.^[^ ^360^ ^]^ In order to facilitate the Li^+^ ion transport along the edge plane, edge‐selectively functionalized graphene NSs have been prepared through various synthetic ways.^[^ ^361^, ^362^, ^363^ ^]^ For instance, Xu et al. synthesized edge‐selectively halogenated graphene nanoplatelet (GnP) by a simple ball‐milling procedure with hydrogen (H_2_), chlorine (Cl_2_), bromine (Br_2_) or iodine (I_2_), which denoted as HGnP, ClGnP, BrGnP or IGnP, respectively (Figure 13B).^[^ ^363^ ^]^ Among the GnPs with selective‐edge halogenation, IGnP material delivered the best reversible capacity as well as cyclability showing a capacity of 464.1 mAh g^−1^ at a current density of 0.25 A g^−1^ over 600 cycles, as depicted in Figure 13C. The observed high capacity and cyclability for IGnP could be attributed to the expanded surface area by the large atomic size, providing many spaces for Li^+^ ion accommodation. In addition, high electronegativity and low electrostatic repulsion of iodine atoms facilitated Li^+^ ion intercalation/deintercalation behaviors along the edge side.

Even though graphene is highly crystalline with excellent electronic character, it does not offer superior performance for Li^+^ storage in its pure form. Prevailing approaches, including intercalation by electrochemically active compounds, edge functionalities, heteroatom substitutional doping, grain boundaries, defect creation, and porous morphologies, significantly contribute to the performances of graphene‐based materials in Li^+^ storage. Materials optimization by controlling synthesis parameters can also result in superior Li^+^ storage.

### 4.3 CNTs for Li‐Ion Storage

CNTs are exciting materials that could offer enhanced specific capacities in LIBs without being susceptible to pulverization.^[^ ^364^, ^365^ ^]^ The tunable morphology of CNTs with desirable properties, including high tensile strength (up to 60 GPa), excellent electrical conductivity (10^6^−10^5^ S m^−1^), and relative inertness, make them a good candidate as anodic materials in LIBs.^[^ ^366^, ^367^ ^]^ According to the theoretical calculations, Li^+^ ions could be adsorbed on the interior as well as the exterior of nanotubes. Especially, intercalation of Li^+^ ion could be markedly facilitated on CNTs with topological defects such as nine‐membered rings and open‐ended nanotube,^[^ ^368^ ^]^ estimating the intercalation density up to LiC_2_ (>1116 mAh g^−1^) which surpasses the limit of conventional graphite.^[^ ^369^, ^370^ ^]^

#### 4.3.1 Defects of CNT

**Figure 14:**
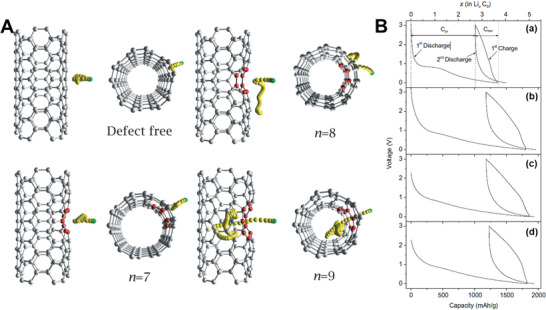
A) Snapshots of the lithium diffusion to the defect‐free hexagonal ring, *n* = 7, *n* = 8, and *n* = 9 defective rings. The initial structure of the SWNT (gray balls and the framework) is superimposed for the eye's guide. The atoms making up the defective ring are indicated by the red balls. The total number of MD time steps is 125 (250 fs) and only the lithium trajectory for every five MD steps are shown (yellow balls). The initial position of the lithium is indicated by the green balls. Reproduced with permission.^[^ ^371^ ^]^ Copyright 2005, American Physical Society. B) Voltage profiles of Li^+^ ion behavior during charging/discharging process with (a) the unetched MWCNTs and the etched MWCNTs for (b) 5, (c) 10, and (d) 20 h. Reproduced with permission.^[^ ^372^ ^]^ Copyright 2004, Elsevier.

Creating defects on the surface of CNTs could significantly enhance the adsorption and diffusion properties of Li^+^ ions. Nishidate and Hasegawa simulated Li^+^ ion adsorption and diffusion behaviors of SWCNT with defective rings on the sidewall, observing the lower energy barrier as the defective ring becomes larger.^[^ ^371^ ^]^ As illustrated in **Figure** **14**A, the SWCNT with enneagon defects only allowed the Li^+^ ion diffusion into the interior, while the Li^+^ ion could not diffuse into the SWCNTs with defect‐free hexagon, heptagon defects, and octagon defects. On the basis of these theoretical calculations, Eom et al. proved the beneficial influence of defects on the LIBs performance of CNTs.^[^ ^372^ ^]^ The authors introduced numerous defects on the surface of multi‐walled CNTs by a chemical etching method using mixed H_2_SO_4_ and HNO_3_ solution. Figure 14B depicts the evolution of electrochemical performance for Li^+^ ion storage in the alteration of etching time. Etched CNTs were more effective in reversible Li^+^ ion storage delivering a capacity of 681 mAh g^−1^ upon 10 h etching compared to pure CNTs (351 mAh g^−1^). The observed enhancement in reversible capacity could attribute to high surface area, many defect sites, and open ends, facilitating the Li^+^ intercalation into the interior of CNTs. However, higher irreversible capacities for etched CNTs were also observed and it might be due to the existence of surface functional groups during the chemical etching process, leading to the larger SEI formation by the reaction with electrolyte in the initial cycle.

Substitution of heteroatoms such as N, S, B, and P is also one of the promising approaches not only for creating the defects on CNTs to act as active sites for Li^+^ ion accommodation but also for providing charge carriers, which increases the electronic conductivity. For example, Wang et al. prepared the B‐substituted CNTs film by a modified floating catalyst CVD method.^[^ ^373^ ^]^ The present B‐substituted CNTs film delivered higher reversible capacity and rate capability than the pure CNTs film. In order to determine the feasibility of these films toward flexible LIBs, the authors also measured the tensile strength curves. The B‐substituted CNTs film exhibited superior toughness and stretchability up to 50% of strain without any fragmentation over pure CNTs one enduring only 15% strain, which might be attributed to the growth of inherent kinks caused by substituents leading to the improved tensile strength.^[^ ^374^ ^]^

#### 4.3.2 Structure Modification of CNTs

Two major factors of the CNTs structure could significantly influence the adsorption and diffusion characteristics of the Li^+^ ion storage. One is the diameter and the other is the length of CNTs. Several theoretical calculations explained that the tube diameter is the important factor that determines the chemical interaction of Li—C during lithiation.^[^ ^375^, ^376^, ^377^ ^]^ For instance, Liu et al. investigated the correlation between the CNTs diameter and Li^+^ ion adsorption energy.^[^ ^375^ ^]^ As increasing the CNTs diameter, the Li^+^ ion adsorption energy of the inside nanotube increased while that of the outside nanotube decreased. The point at which these two adsorption energies became similar was when the diameter was greater than 0.824 nm. In addition, the pore structure and surface area of CNTs could be altered by tuning the tube diameter, which is an important parameter to boost the Li^+^ ion storage capability. In order to experimentally prove the effect of diameter on LIB performance, Zhang et al. reported the LIB capability of commercial CNTs in the alteration of the tube diameter.^[^ ^378^ ^]^ Distinct discrepancy in electrochemical properties was observed depending on the diameter. When the sizes of CNTs are within 40−60 nm range, they delivered the highest reversible capacity of 187.4 mAh g^−1^ as well as the highest conductivity. The observed electrochemical results could be ascribed to the enhanced charge transfer during lithiation and delithiation processes caused by optimized pore structure and surface area. The length of CNTs, which is another critical factor influencing the LIB performance, significantly affects the Li^+^ ion diffusivity as the short CNTs can promote the Li^+^ ion intercalation into the inside of the tube through open ends. However, creating many lateral defects with functional groups is inevitable to synthesize the short CNTs, which may have an adverse effect on the electrochemical properties of CNTs, including electrical conductivity and lithiation/delithiation potentials.^[^ ^379^ ^]^

#### 4.3.3 Unzipping of CNT

**Figure 15:**
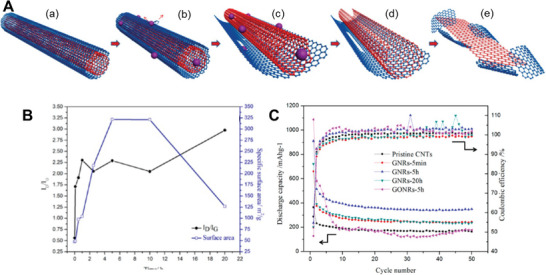
A) Schematic illustration of (a) pristine CNTs, (b) etched CNTs, (c) partially unzipped CNTs, (d) fully unzipped CNTs, and (e) stacked GNRs fragments. B) Plot of the *I*_D_/*I*_G_ ratio from Raman data and surface area. C) Discharge capacity with CE of pristine CNTs, GNRs, and GONRs at a current density of 0.1 A g^−1^. Reproduced with permission.^[^ ^385^ ^]^ Copyright 2014, American Chemical Society.

The longitudinal unzipping process of CNT could lead to the successful formation of zigzag graphene nanoribbons (GNRs), which not only form stronger interaction with Li^+^ ion but also possess a high lithium diffusion coefficient of two orders of magnitude compared to graphene NSs.^[^ ^360^, ^380^ ^]^ Initial unzipping processes of CNT began from a way of using oxidizing reagents to diverse synthetic strategies such as plasma etching, edge cutting by metal catalysts, insertion/exfoliation, and ultrasonic process.^[^ ^381^, ^382^, ^383^, ^384^ ^]^ By controlling the parameters of synthetic procedures, the unzipping level of GNRs and the surface area, defects, and functionalized degrees could be finely tuned and optimized as appropriate for the performance of LIBs. Xiao et al. investigated the correlation between the morphological evolution of CNTs during the unzipping process and LIB performance.^[^ ^385^ ^]^ Five minutes of etching led to some defects on the surface but still maintained the original tubular morphology of CNTs (**Figure** **15**A‐b). From these defects, it started to unzip the outer CNTs gradually (Figure 15A‐c), and CNTs were eventually fully unzipped longitudinally (Figure 15A‐d). Further etching, however, caused severe CNTs fragmentation partially with flat and small particulate morphology (Figure 15A‐e). *I*_D_/*I*_G_ ratios, which indicate the degree of defects formed on CNTs (Figure 15B), showed a remarkable increase from 0.05 to 2.25 within 1 h, inferring that most of the defects during the unzipping process occurred at the initial state. Significant enhancement in terms of surface area was observed from 47.3 to 321 m^2^ g^−1^ as increasing the etching time up to 5 h due to the gradual opening of tubular structures. However, the extra etching process to 20 h resulted in dramatic depression in surface area to 126.6 m^2^ g^−1^, which could be ascribed to the fragmentation and concomitant aggregation of CNT particles. According to the evolution of the textural properties of GNRs during the unzipping process, it was revealed that the unzipping‐controlled GNR with 5 h etching process generated the highest specific capacity thanks to the optimized condition of the highest surface area and a large number of defects (Figure 15C).

From this subsection, it is well acknowledged that defects, doping, and functionalities help improve electrochemical sites. Porosity helps in enhancing access to those sites, thereby giving rise to enhanced Li^+^ storage. CNT unzipping in general creates edges, which can conveniently be edge‐doped or edge‐functionalized. This opens up a huge opportunity for physicochemical material modification in CNTs, therefore, controlled unzipping and subsequent doping can lead to superior Li^+^ ion storage.

### 4.4 Fullerene for Li‐Ion Storage

Fullerene (C_60_ and C_70_) is an interesting form of hollow spherical carbon and the previous studies have confirmed that C_60_ can theoretically accommodate 1 Li^+^ ion for every 5 carbon atoms and demonstrate a higher Li^+^ ion storage theoretical capacity of 446 mAh g^−1^ as compared to graphite.^[^ ^386^ ^]^ However, fullerene‐based carbon nanostructures suffer from a low reversible capacity which hampers their potential applications in batteries addressing the high energy demand. The research in the fullerenes has stressed on the synthesis of hybrid systems of fullerenes with other carbon sources,^[^ ^387^ ^]^ metal oxides/alloys, and other suitable alliances such as semiconductors for energy storage in LIBs.^[^ ^16^, ^17^ ^]^ Si has been regarded as one of the most efficient components with fullerene in a hybrid structure. The hybridization with fullerene can be expected to mitigate the main vulnerability of Si material, which is related to the extremely large volume expansion and resulting in severe irreversible capacity.^[^ ^388^ ^]^ Additionally, it was found that fullerene could help to enhance structural stability. In one typical work, Park and co‐workers developed novel malonic‐acid‐functionalized fullerene, which served as a superoxide dismutase mimetic electrolyte additive to deactivate reactive radical species and to scavenge trace water to avoid undesirable hydrolysis.^[^ ^389^ ^]^ Moreover, the authors found that such fullerene could improve the structural stability of cathodes and decrease the parasitic reaction of residual lithium compounds with LiPF_6_ by forming a cathode‐electrolyte interface. The research in the field of fullerene‐based electrodes in LIBs has gained momentum in the recent past, and it will continue to reveal the improved benefits of these materials for LIBs.

In short, carbon nanomaterials have been intensively exploited as anode materials thanks to their high capacity and rate capability; they have also widely been employed as conductive additives with conventional electrode materials due to their excellent electron mobilities. Similar to supercapacitors, these nanostructured carbon materials for LIB also suffer from aggregation and synthesis issues. Therefore, strategies like assembling with other 3D‐ordered materials and nano‐architectures, including aligned arrays, uniform foams, and pillared hybrids, can be attempted, which could further enhance the rate capability and cyclability of LIBs. Furthermore, the surface functional groups of these nanostructured carbon electrodes are extremely important to redox processes, thus deciding the final device performance. To this end, the combination of controlled extent, nature of doping, and adequate surface/edge functionalization should be systematically investigated.

## 5 Sodium‐Ion Battery

### 5.1 Introduction to Sodium‐Ion Battery

Sodium is the fifth most abundant element in the earth's crust and the second lightest alkali element in the periodic table as the charge carrier.^[^ ^390^, ^391^ ^]^ Even though the research on sodium‐ion batteries (SIBs) began in the 1970s alongside LIBs, they were sidelined due to their inferior electrochemical properties compared to their lithium counterparts.^[^ ^392^ ^]^ Recently, concerns about the scarcity of lithium sources and increasing costs have led to a renewed interest in SIBs, and a tremendous amount of research has been devoted to the discovery of new materials as anodic electrode materials. However, the issues, including sluggish kinetics, low operating voltage limits, low energy/power density, and poor longevity, still need more research in this field. The development of novel electrode materials with optimized electrolyte compositions can help address these issues and advance this technology to meet the benchmark requirements of its lithium counterpart.^[^ ^393^ ^]^

Another drawback of SIBs is that the higher atomic weight of sodium results in lower volumetric or gravimetric capacities than LIBs. Therefore, a suitable electrode configuration with optimized material systems is required to compensate for the capacity loss. In the past decades, various materials have been studied as electrode materials for SIBs, such as layered oxides,^[^ ^394^, ^395^ ^]^ fluorides,^[^ ^396^ ^]^ polyanionic compounds,^[^ ^397^, ^398^ ^]^ sulfates,^[^ ^399^, ^400^, ^401^ ^]^ phosphates, pyrophosphates,^[^ ^402^, ^403^ ^]^ Prussian blue analogs,^[^ ^404^ ^]^ and organic polymers.^[^ ^405^ ^]^ The lack of an anodic electrode system that can accommodate sodium‐ion with a larger ionic radius (1.06 Å) compared to lithium‐ion (0.76 Å) has been the main reason hindering the practical applications of SIBs. Graphite, the commercialized anode material for LIB industries, was found to be unsuitable for SIB systems. While the lithium‐inserted graphite could form the LiC_6_ via the stage‐I intercalation process, the intercalation of Na^+^ ion into the graphite caused the NaC_64_, which could be anticipated to possess a very limited theoretical capacity of 30 mAh g^−1^.^[^ ^406^ ^]^ This estimated low theoretical capacity is responsible for the too narrow interlayer distance of 0.34 nm to be intercalated and chemically unstable formation of Na—C bonds.^[^ ^407^, ^408^ ^]^ Nevertheless, a number of researchers have attempted to employ carbonaceous materials into the SIBs applications with the similar strategies to LIBs under the reminiscence of the tremendous success of LIBs systems, which will be discussed in this section.

### 5.2 Graphene for Sodium‐Ion Storage

#### 5.2.1 Pure Graphene/rGO for Sodium‐Ion Storage

**Figure 16:**
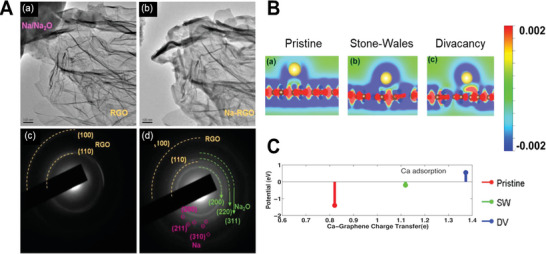
A) In situ TEM images of the initial sodiation behavior of rGO which is before sodiation (a) and after sodiation (b) and its diffraction patterns for (c) before sodiation and (d) after sodiation. B) Bonding charge density of Na for pristine (a), Stone−Wales (b, SW), and divacancy (c, DV) systems obtained as the charge‐density difference between the valence charge density before and after the bonding. Red and blue colors represent electron accumulation and depletion, respectively. C) the graph of potential vs charge transfer for Na adsorption. Reproduced with permission.^[^ ^412^ ^]^ Copyright 2014, American Chemical Society.

It is quite straightforward to predict that the graphene sheets would also be utilized as an anode electrode for SIB systems. In 2013, Wang et al. for the first time demonstrated that rGO fabricated by the modified Hummer's method could deliver promising capability for Na^+^ ion storage with stable cyclability over 1000 cycles, a reversible capacity of 141 mAh g^−1^ at a current density of 40 mA g^−1^, and good rate capability.^[^ ^409^ ^]^ In addition, Chung et al.^[^ ^410^ ^]^ synthesized rGO via microwave irradiation which showed a stable discharge capacity of 240 mAh g^−1^ for up to 1000 cycles, demonstrating the utility of rGO NSs for operating the SIB system. Furthermore, in order to investigate the electrochemical behavior of rGO sheets during sodiation/desodiation process, Wan et al. carried out in situ TEM analysis.^[^ ^411^ ^]^ As shown in **Figure** **16**A, flexible sheet morphology with the diffraction patterns of (110) and (100) planes was observed for rGO NSs before initial sodiation. After the initial sodiation behavior, thin rGO sheets became slightly coiled up, together with the appearance of Na_2_O and Na phases according to the diffraction patterns.

Similar to LIBs, defective graphene has also received prime attention based on theoretical calculations. Shenoy et al.^[^ ^412^ ^]^ did first‐principles calculations and investigated the Na adsorption property on the defective sites of graphene, including divacancy (DV) and Stone‐Wales (SW) defects, and provided guidelines to fabricate high‐performing anode materials for SIBs. As shown in Figure 16B, the distribution of bonding charge density for Na atoms was changed depending on the defect types of graphene NS, facilitating the charge transfer from Na atoms and graphene NSs and concomitantly the adsorption of Na atoms. According to the quantified charge transfer simulation, pristine, SW‐ and DV‐defective graphene appeared to be 0.6617*e*, 0.8073*e*, and 0.8848*e*, respectively, suggesting that the notably enhanced charge transfer upon the introduction of defects on the surface of graphene NS. In addition, as can be seen in Figure 16C, the potential of SW‐ and DV‐defective graphene could be reached for the favorable stage of Na atom adsorption. The experimental results confirmed that the enhancement of Na adsorption was observed with the surface of defective graphene, leading to facilitated charge transfer kinetics. Interestingly, the obtained maximum specific capacities were far higher than that of defect‐free graphite and these could be significantly increased up to 2,000 mAh g^−1^ and 2,142 mAh g^−1^ for divacancy defects and Stone‐Wales defects, respectively. Structural modification of graphene sheets can also help in improving the electrochemical capacity of SIBs. Textural parameters such as well‐defined porosity, ordered pores, and a large specific surface area could assist in accommodating the diffusion of the large‐sized Na^+^ ions. Previous literature has confirmed that enlarged lattice spacing between graphene layers could help in minimizing the volume expansion and hence an improved sodium ion storage can be achieved.^[^ ^413^, ^414^ ^]^

#### 5.2.2 Heteroatom Doped Graphene

**Figure 17:**
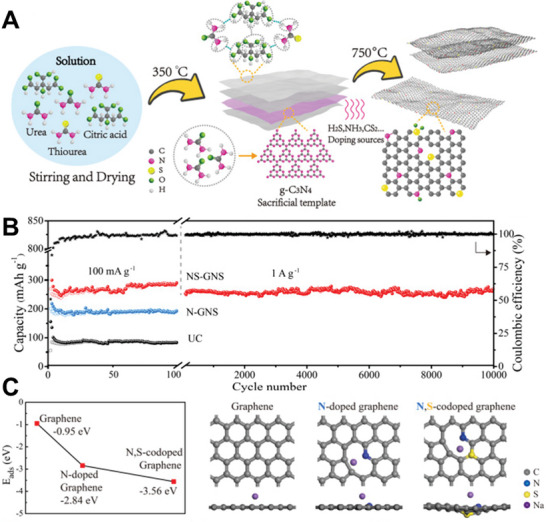
A) Schematic illustration of N, S‐doped graphene NSs synthesis. B) Long‐term cyclability of N, S‐doped graphene NSs at a current density of 1 A g^−1^ together with Coulombic efficiency. C) Comparison in adsorption energies of pristine, N‐doped and N, S‐doped graphene toward Na^+^ ions with corresponding geometry configurations. Reproduced with permission.^[^ ^421^ ^]^ Copyright 2018, Elsevier.

As mentioned in the earlier part of LIBs, the introduction of foreign atoms in the lattice of graphene structure has attracted great attention owing to its fascinating electrochemical properties when applied as the anode electrode for rechargeable batteries.^[^ ^415^ ^]^ Especially, taking into account the fact that the larger size of Na^+^ ion than Li^+^ ion adversely affects the electrochemical kinetics and SIB functionalities, the introduction of heteroatoms in graphene lattice could expand the interlayer distance, facilitating the reversible intercalation/deintercalation reactions of Na^+^ ions into graphene.^[^ ^416^ ^]^ Therefore, it has been regarded as a powerful strategy to secure the high‐performing SIB system. Numerous attempts were also made to dope other types of heteroatoms in pristine graphene in a search for fabricating high‐performance SIBs. Many reports have demonstrated the effective role of various heteroatoms in the electrochemical performance of graphene or rGO as the anode material for SIBs system.^[^ ^336^, ^417^, ^418^, ^419^ ^]^ For example, Xu et al. prepared the N‐doped graphene foam (N‐GF) by annealing a freeze‐dried graphene oxide in ammonia which delivered 2 times higher specific capacity (605.6 mAh g^−1^) than undoped GF (329.6 mAh g^−1^).^[^ ^413^ ^]^ In a similar manner, C atoms of graphene lattice have also been replaced by S atoms possessing higher electronegativity characteristics.^[^ ^420^ ^]^ Ma et al. synthesized N, S co‐doped graphene NSs from a couple of precursors containing N, S, and C elements, as depicted in **Figure** **17**A.^[^ ^421^ ^]^ Co‐doped graphene NSs appeared to deliver a larger specific capacity than single N‐doped one which could be ascribed to the lowest adsorption energy of N, S co‐doping, triggering favorable behavior for the redox reaction in the vicinity of dopants (Figure 17B,C). In addition to S‐doping, Ling et al. demonstrated the feasibility of B‐doped graphene sheets simulating the first principles calculations of an achievable reversible capacity of 762 mAh g^−1^.^[^ ^422^ ^]^ Upon the B‐doping, electron clouds in the B—C bonds become more localized to C atoms as the B atom acts as a P‐type dopant, leading to the stabilization of Na^+^ ion adsorption on the electron poor sites by receiving the electrons from Na. Despite these positive results from simulation, very limited experimental studies have been conducted on it due to the synthetic difficulties in the preparation of B‐doped graphene without additional formation of boron carbide.^[^ ^342^ ^]^ Iodine and sulfur heteroatoms co‐doped graphene also exhibited increased specific capacity. The enhanced performances were attributed to the defect evolution and enlarged interlayer distance between graphene layers due to the presence of large sized heteroatoms.^[^ ^423^, ^424^ ^]^

Thus, graphene in its pure form and heteroatom‐doped graphene have been attempted for their application in Na‐ion batteries. Interplanar spacing and redox‐inertness of pure graphene are crucial issues, which have been partially addressed by hetero‐atom doping. Intercalation by CNTs and fullerenes can further help enhance performance as they will provide channels for swift ion transport. Moreover, the design of porous architectures will help further in attaining superior electrochemical performance.

### 5.3 CNT for Sodium‐Ion Storage

Owing to the excellent mechanical and electrochemical properties, CNTs have been regarded as promising materials for SIBs.^[^ ^425^ ^]^ However, due to its narrow interlayer spacing of 0.34 nm, the electrochemical accommodation of larger Na^+^ ion could be significantly limited, showing only a specific capacity of 100 mAh g^−1^ at a current density of 0.03 A g^−1^.^[^ ^426^ ^]^ In order to address this issue related to the narrow interlayer spacing of CNT, Sundara et al. prepared partially unzipped MWCNTs by using Hummer's method, which are composed of inner shell of MWCNTs and outer shell of graphene nanoribbon, possessing the expanded interlayer spacing of 0.8 nm.^[^ ^427^ ^]^ As compared to the bare MWCNTs in terms of electrochemical functionality toward Na^+^ ion accommodation, partially unzipped MWCNTS delivered ≈200 mAh g^−1^ at a current density of 0.05 A g^−1^, which is two times higher value than the bare MWCNTs (≈100 mAh g^−1^). In the charge–discharge profile of these materials, the bare and partially unzipped MWCNTs exhibited the specific capacities of 50 and 150 mAh g^−1^, respectively, at a current density of 0.02 A g^−1^ in the potential region higher than 0.7 V which is directly indicative of the Na^+^ ion intercalation, clearly suggesting that the enlarged interlayer spacing of partially unzipped MWCNTs facilitated the Na^+^ ion insertion.

Notably, there is a distinct limitation in the SIB performance that could only be achieved by using the CNTs. Therefore, like any other carbonaceous materials, CNTs are mainly used as a conductive matrix with lower weight loading. CNTs have the capability of being assembled as free‐standing electrodes (in the absence of any binder or current collector) in the pristine form or as physical support to realize ultra‐high capacity electrode materials like silicon, germanium, metal oxides etc.^[^ ^428^ ^]^ Zhou et al. synthesized a composite with uniform antimony (Sb) NPs and MWCNTs by wet milling.^[^ ^429^ ^]^ High electronic transportation and a stable SEI were achieved by optimizing the ratio of Sb and MWCNT. When tested as an anode in SIBs, the as‐prepared Sn/CNT composite delivered a high capacity of 502 mAh g^−1^ at a current density of 0.1 A g^−1^ and 76.1% retention rate over 120 cycles with excellent rate capability. Similarly, Guo and co‐workers developed a binder‐free anode using a binary self‐assembly composite of Bi_4_Se_3_/Bi_2_O_2_Se embedded in CNTs and graphene.^[^ ^430^ ^]^ The as‐obtained Bi_2_O_2_Se/Bi_4_Se_3_@CNTs@rGO exhibited excellent sodium‐ion storage with a reversible capacity of 346 mAh g^−1^ and a stable performance of more than 50 cycles. Notably, the unique “one‐changes‐into‐two” phenomenon, where the layered Bi_4_Se_3_ transforms into a layered Bi_4_Se_3_/Bi_2_O_2_Se heterojunction structure, enhances the material's electrochemical channels and stability. Graphene‐CNT aerogels have been employed in Na‐ion batteries. In a typical work, Sun et al. developed a new method for producing a red phosphorus@nitrogen doped graphene/CNT aerogel (P@NGCA) for use as a free‐standing anode in sodium‐ion batteries.^[^ ^431^ ^]^ The P@NGCA anode is created by uniformly distributing red phosphorus into a substrate made of NGCA using self‐assembly, freeze‐drying, and vaporization‐condensation methods. The 3D “reinforced concrete” structure of the P@NGCA anode helps to alleviate volume expansion and increase conductivity, enabling high areal capacity of 3.3 mAh cm^−2^, superior rate capability, and a high initial Coulombic efficiency of 80%.

However, a low retention rate is always a hurdle to be utilized in practical SIBs. Conductive coating with CNTs has been regarded as one of the ideal approaches to address this issue.^[^ ^432^ ^]^ Li et al. reported that the commercially available micro‐sized red phosphorous and CNT (P/CNT) composite synthesized by simple hand grinding showed an exceptional reversible sodium ion storage capacity of 1675 mAh g^−1^.^[^ ^433^ ^]^ This enhanced capacity is a result of the wrapping of phosphorous particles by CNT, which helped in improving the electronic conductivity, buffering the volume expansion and reducing the particle pulverization. Another issue of CNT‐based SIBs lies in their relatively short lifetime and low stability, so an in‐depth understanding of the degradation mechanism is urgently required. To this end, Zheng and co‐workers recently first realized the in‐situ observation of the sodiation/desodiation processes of MWCNTs by TEM.^[^ ^434^ ^]^ Solid electrolyte interface layers formed on the surface of sodiated MWCNTs, and fish scale‐like sodium dendrites appeared with further sodiation. The sodiation process caused a decrease in the width and increased the brittleness of MWCNTs, potentially due to the intercalation of Na‐ions and loss of carbon from Joule heating, resulting in easily broken after the electrochemical process.

Although CNT in its pure form may not be a great candidate for sodium‐ion batteries, its hetero atom doping and functionalization can enhance electrochemically active sites needed for redox reactions or intercalation. Moreover, its hybridization with 2D/3D materials can render it apt for swift sodium ion transport. While functionalization/doping is carried out, an adequate electrical character including high conductivity should be preserved for the current collection.

### 5.4 Fullerene for Sodium‐Ion Storage

Quite recently, fullerenes have been explored for their role in SIB performance.^[^ ^435^ ^]^ Their high electron affinity implies that they can potentially serve as a cathode material of reasonably high redox potential in batteries.^[^ ^436^, ^437^ ^]^ There are only a few reports on the use of fullerenes in LIBs,^[^ ^438^, ^439^ ^]^ and hence it is expected that these would be the least explored carbon nanomaterials for SIBs. In 1994, Lemont et al. studied the electrochemical intercalation of sodium ions into C_60_ and C_60_/C_70_ mixtures in ionic conducting polymer electrolyte‐based solid state cells.^[^ ^440^ ^]^ In 2018, Wang and co‐workers developed Fe‐based quantum dots (FBQDs) that were tethered onto a hybrid system comprising of Na‐doped C_70_ and N‐doped graphene mixtures to form Na‐doped C_70_/N‐doped graphene/FBQDs nanocomposite with varied morphologies towards the fabrication of SIBs with high QE through the facile in situ approach.^[^ ^441^ ^]^ The as‐developed nanocomposites displayed a unique hybrid carbon structure that offers adequate metallicity, ample bonding sites for Na ions, and a rapid mass transfer of Na‐ions and electrons during long cycling. When tested as an anode material for SIBs, this nanocomposite achieved a high discharge capacity of 1898 mAh g^−1^ at a current density of 1 A g^−1^ and retained a reversible capacity of 238 mAh g^−1^ after 100 cycles. In another case, Scaravonati et al. showed the ability of C_60_ and hydrogenated C_60_ to act as cathode material in SIBs.^[^ ^442^ ^]^ They developed an optimized solid‐state Na‐(polyethylene oxide) electrolyte to address the very well‐known issue of the solubility of C_60_ in common organic electrolytes. The obtained specific capacities for first discharge cycles were 250 mAh g^−1^ and 230 mAh g^−1^ for C_60_ and hydrogenated C_60_, respectively. Due to the formation of a stable Na*_x_* C_60_ phase, where the diffusion of Na^+^ ions is hindered, the C_60_ electrode showed strong irreversible capability after the first discharge cycle. On the other hand, upon intercalation, hydrogenated C_60_ showed better reversibility, suggesting that the hydrogenation of C_60_ could offer significant pathways for sodium ion diffusion. In a recent case, Lu's group intercalated fullerene into g‐CN, in which C_60_ molecules increased the conductivity and prevented 2D g‐CN nanosheets from restacking.^[^ ^443^ ^]^ The increased interlayer spacing of g‐CN and exposed N defect edge were beneficial for Na‐ion storage, leading to a high‐reversible capacity, excellent rate capability, and long cycle life compared to pristine g‐CN. Consequently, a Na‐ion full cell combining the C60@CN anode and an NVPF@rGO cathode exhibited a high‐coulombic efficiency of over 96.5%, the high‐energy density of 359.8 Wh kg_anode_^−1^ (@ power density of 105.1 W kg_anode_^−1^), and excellent cycling stability of 89.2% capacity retention after 500 cycles, which demonstrated that C_60_ could be integrated with other functional materials as anodes for sodium‐ion batteries.

SIBs based on carbon nanomaterial anodes have demonstrated merits with high rate capability and long cycling. In addition, thanks to the short ionic/electronic transport distances as well as good stress tolerance, these materials have been utilized for buffering the volume change of anodes because of Na^+^ ion insertion/extraction. Nevertheless, the existing carbon nanomaterials‐based SIBs still suffer from the following issues: i) The ionic radius of Na^+^ ion is 55% larger than Li^+^, which greatly reduces ion transport at the interlayers of the electrodes. To address it, introducing intercalation agents, doping with large atoms, and multiple functionalization can help. In addition, introducing ordered meso/nano‐porosity in carbon nanomaterials enables large specific surface areas, effectively boosting the access to the electrochemically active sites apt for Na‐ion batteries. ii) Carbon nanomaterial family generally has high oxidation voltages, reversibly offsetting the advantage of larger capacities from carbon materials. iii) Nanostructured carbons present large initial irreversible capacities, possibly due to the electrolyte's decomposition and the formation of the SEI layer. iv) The underlying mechanism of SEI formation in sodium‐ion batteries composed of pure/doped/functionalized graphene/CNTs/fullerenes must be understood well to avoid/minimize them and improve battery performance. Full cell investigation for functionalized carbon nanomaterials‐based SIBs should be focused on next, for future practical applications.

## 6 Electrocatalytic Hydrogen Evolution Reaction

### 6.1 Introduction to Electrocatalytic HER

Extensive research is underway to find alternative energy sources to reduce our dependency on fossil‐based energy sources and decrease the environmental burden of their burning.^[^ ^15^, ^444^ ^]^ As a lucrative source of clean energy, hydrogen has been utilized in clean energy‐producing devices. With the highest gravimetric energy density, hydrogen also burns cleanly and is considered the most promising fuel with zero carbon emission. However, hydrogen production for mass storage and its further utilization is challenging. Hydrogen can be sourced directly from water splitting by electrolysis, photoelectrolysis, or solar thermochemical processes.^[^ ^444^, ^445^, ^446^, ^447^, ^448^, ^449^, ^450^, ^451^ ^]^ Electrolysis is commercially the most successful among these processes. The electrolysis of water proceeds with the hydrogen evolution at the cathode (HER) and oxygen evolution at the anode (OER) through a series of reactions occurring at each electrode.^[^ ^19^, ^452^ ^]^ Despite more than a century of research in water electrolysis, the efficiency of water splitting only ranges from 56 to 73% because of losses of overpotential, low turnover frequency, and so on.^[^ ^453^, ^454^ ^]^

One of the main factors determining the HER efficiencies is the pH of the medium. In an acidic medium, HER reaction is predicted to go through two mechanistic pathways a) Volmer‐Heyrovsky (Equations 1 and 2) or the Volmer‐Tafel pathway (Equations 1 and 3):^[^ ^455^ ^]^

(1) $$H^{+}+{\;e}^{-}\;\to {\;H}^{*}$$

(2) $$H^{*}+H^{+}+e^{-}\to H_{2}$$

(3) $$2H^{*}\to H_{2}$$

As evident from the reactions, the formation of nascent hydrogen and its reaction with another solvated hydrogen ion or nascent hydrogen is central to hydrogen evolution. HER is thus dependent heavily on the interaction between the nascent hydrogen and the catalyst surface. The efficiency of various catalysts to bind and release hydrogen was compared by Trasatti using a first of its kind volcano curve by comparing the HER rates with hydride formation energy.^[^ ^456^ ^]^ Since then, the volcano plots have been modified and supported by DFT calculations to correctly represent the hydrogen adsorption energy and the HER reaction rates.^[^ ^457^ ^]^ The superiority of platinum over all other catalysts was realized based on the volcano curve and the quest for new materials started by comparing their activities based on their placement on the volcano curve. However, the high costs of platinum (and other noble metal catalysts such as Rh and Ir) combined with moderate efficiencies results in high commercialization barrier and thus electrolysis currently contributes to only 4% of the overall commercial hydrogen generation.

In alkaline media, on the other hand, the hydrogen atoms are extracted from the water in the Volmer step (Equation 4) and the Heyrovsky step (Equation 5), while the Tafel (Equation 6) reaction proceeds with the direct reduction of hydrogen from water molecules.^[^ ^458^ ^]^ The requirement to generate protons in alkaline media is an energy‐intensive step and leads to sluggish HER kinetics. Thus, various transition metal‐based catalysts do not work well in alkaline media as they are inefficient in dissociating water molecules.

(4) $$H_{2}O+e^{-}\to H^{*}+{\mathrm{OH}}^{-}$$

(5) $$H^{*}+H_{2}O+e^{-}\to H_{2}+{\mathrm{OH}}^{-}$$

(6) $$2H_{2}O+e^{-}\to H_{2}+2{\mathrm{OH}}^{-}$$

**Figure 18:**
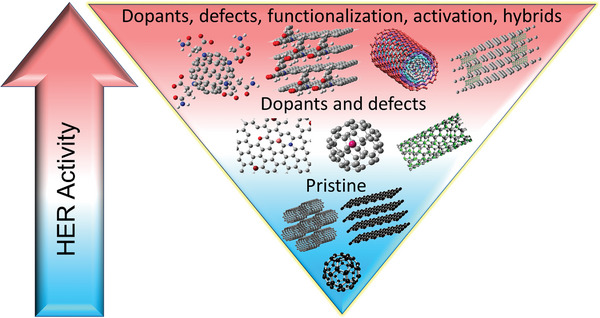
Schematic representation of various forms of carbon and their modifications to increase their HER activity.

Two major approaches that have been tried extensively to reduce the high‐cost barriers associated with noble metal‐based catalysts and improve the efficiency of water electrolysis are a) increasing the use of non‐noble metal based catalysis and b) use of metal‐free catalysts. Carbon‐based materials have always been the focus of HER research owing to their high surface area, fast charge transfer kinetics, high conductivity, and excellent chemical stability to facilitate HER processes, enabling them to be ideal materials for both acidic or alkaline electrolysis as support materials and/or active HER catalysts.^[^ ^455^, ^459^, ^460^, ^461^, ^462^, ^463^, ^464^, ^465^, ^466^, ^467^, ^468^, ^469^, ^470^, ^471^, ^472^, ^473^ ^]^ This section will focus exclusively on developing metal‐free carbon nanomaterials as HER electrocatalysts. The focus is on using carbon materials independently, and hybrids of different carbon materials were excluded except where necessary for giving credit to the first reported approach (**Figure** **18**). The most valuable parameters, including overpotential, onset potential, and Tafel slope, are the key parameters for comparing the HER activities across various carbon nanostructures.

### 6.2 Graphene‐Based Electrocatalytic HER

#### 6.2.1 Heteroatom Doped Graphene for Electrocatalytic HER

As highlighted throughout this review, graphene has emerged as an excellent material owing to its unique electronic properties emerging from its 2D planar geometry. However, pristine graphene shows low activity due to relative inertness towards HER, which arises from its conducting or zero bandgap nature, resulting in either weak or no catalytic activity. Its graphite‐like sheets with sp^2^ hybridized stable electronic structure are of very low energy and do not represent highly active sites for the adsorption of analytes.^[^ ^474^ ^]^ Consequently, doping graphene with heteroatoms such as B, N, O, S, and F has been extensively attempted to enhance its water‐splitting activity. In heteroatom doping, selected carbon atoms in the graphene ring structure are replaced by one or multiple heteroatoms. In general, heteroatom doping can induce an enhancement of HER catalytic activity.^[^ ^470^, ^475^ ^]^ Due to the difference in size and electronegativity of heteroatoms compared to carbon, dopants can effectively modulate the electron density and charge distribution in the ring structure, thus providing highly active sites for catalytic water‐splitting reactions along with high conductivity for fast electron transfer in their applications as electrode materials. In addition, both doping contents and dopant positions play decisive roles in imparting electrocatalytic properties to graphene.^[^ ^463^ ^]^

##### N‐Doped Graphene

The first report on using heteroatom‐doped graphene for electrocatalytic ORR appeared in 2010, where the N‐graphene was formed by passing ammonia vapors in a typical CVD process for the synthesis of graphene from methane.^[^ ^476^ ^]^ The highly improved catalytic activity of N‐graphene as an electrode material for ORR demonstrated three times higher steady‐state catalytic current over a large range of potential values as compared to the Pt/C. However, the first use of N‐doped graphene for HER was only achieved in 2014; until then, it was used mostly for ORR activity.^[^ ^477^ ^]^ A combination of carbon nitride (C_3_N_4_) and N‐graphene nanohybrid was created to combine the hydrogen adsorption properties of C_3_N_4_ with the electron transfer capability of N‐graphene. The strong coupling between the C_3_N_4_ and N‐graphene resulted in changing the nature of C_3_N_4_ from semi‐conducting to metallic due to the absence of a band gap. The free electron flow from N‐graphene to C_3_N_4_ resulted in a redistribution of charge density on C_3_N_4_ and the presence of a hole‐rich region in the N‐graphene. The HER parameters such as overpotential (≈240 mV), Tafel slope (‐0.51 mV dec^−1^) and exchange current density (3.5 × 10^−7^ A cm^−2^) showed superior HER activity of the nanohybrid that is comparable to MoS_2_, Cu, and Ni based catalysts (surface area normalized) and better than many other metal catalysts such as W, Co, Au (111) and Ag (111).^[^ ^477^ ^]^ The location of nitrogen atom in the doped structure of graphene is important and is related to the improved catalytic activity.

Nitrogen atoms can be present in the ring structure as pyrrolic, pyridinic, or graphitized nitrogen. Each of these configurations results in different types of doping effects, thereby affecting its catalytic properties and electronic conductivity.^[^ ^478^ ^]^ For example, the pyrrolic and pyridinic nitrogen can contribute two and one electrons to the *π* system of graphene. However, graphitic nitrogen in the right structure is considered as the most efficient in creating n‐type doping effect.^[^ ^479^ ^]^ These observations exclude the edge effects. N‐graphene has dominated the studies on their applications for HER as compared to any other dopant in the p block.^[^ ^480^ ^]^ Recently, selective N‐doped graphene (oxide) with as high as 51% pyridinic nitrogen content was synthesized using laser irradiation even though the overpotential remained much higher ≈590 mV as compared to what is generally achieved with dopants.^[^ ^481^ ^]^

**Figure 19:**
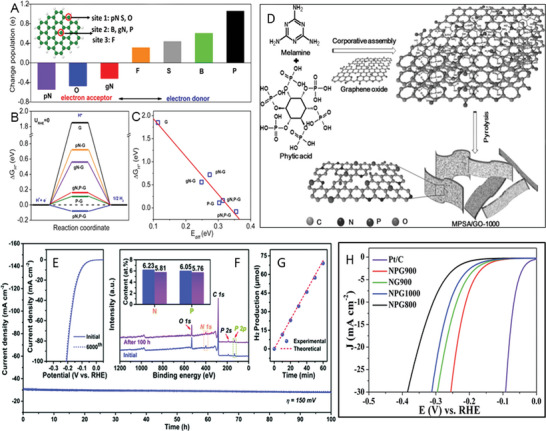
A) NBO population analysis of six different non‐metallic heteroatoms in graphene matrix. pN and gN represent pyridinic and graphitic type of N, respectively. Inset shows the proposed doping sites for different elements, sites 1 and 2 are the edge and center in‐plane sites, respectively, and site 3 is an out‐of‐plane center site in graphene. B) The calculated free energy (Δ*G*_H*_) diagram for HER at the equilibrium potential (*U*_RHE_ = 0 V) for N‐ and/or P‐doped graphene models. C) Relationship between Δ*G*_H*_ and *E*_diff_ for various models. Reproduced with permission.^[^ ^475^ ^]^ Copyright 2014, American Chemical Society. D) Schematic of the cooperative self‐assembly and pyrolysis process for the synthesis of N, P doped GO. Reproduced with permission.^[^ ^482^ ^]^ Copyright 2015, Wiley‐VCH. E) iR‐compensated time dependence of the current density for G‐12NP in 0.5 m H_2_SO_4_ for 100 h (*η* = 150 mV). The inset images are iR‐compensated HER polarization data for G‐12NP in 0.5 m H_2_SO_4_ initially and after 6000 CV sweeps between −0.1 and −0.6 V (vs RHE). F) Survey XPS spectra of G‐12NP initially and after 100 h durability tests and the corresponding atomic percentages of N and P. G) Faradaic efficiency of G‐12NP in 0.5 M H_2_SO_4_ (*η* = 150 mV). Reproduced with permission.^[^ ^483^ ^]^ Copyright 2019, Royal Society of Chemistry. H) HER polarization curves for various N, P doped graphene as compared to Pt/C shows that N, P doped graphene at 900 °C gives the lowest overpotential. Reproduced with permission.^[^ ^484^ ^]^ Copyright 2015, Royal Society of Chemistry.

Soon after, double dopants in graphene were also fabricated to improve the HER activity. Based on the initial results from theoretical calculations, two dopants with the opposite effect on graphene in terms of electron acceptor (i.e., nitrogen) and donor (i.e., phosphorus) properties were combined to create dual dopant graphene for HER activity. The free energy of hydrogen adsorption correlates with the HER activity and a combination of pyridinic N co‐doped with P showed the minimum energy for hydrogen adsorption, as compared to most of the dopants in one of the doped graphene models (**Figure** **19**A–C).^[^ ^475^ ^]^ The HER activity tested in both acidic (0.5 m H_2_SO_4_) and alkaline (0.1 m KOH) shows the overpotential values of ≈420 mV and ≈590 mV, respectively, which were comparable to some of the metals. Using a surface functionalization‐pyrolysis approach, 3D porous N and P co‐doped graphene were developed from a combination of melamine, phytic acid, and graphene oxide.^[^ ^482^ ^]^ Epoxy and hydroxyl groups on the surface of GO reacted with melamine and phytic acid in a cooperative self‐assembly process to create supermolecular aggregates, as shown in Figure 19D. The catalyst obtained following pyrolysis of this supermolecular structure demonstrated bifunctional ORR‐HER activity. Specifically, HER activity increased with an increase in pyrolysis temperature, and the catalyst synthesized at 1000°C demonstrated an overpotential of only 60 mV, a result comparable to some of the best 2D metal chalcogenide catalysts. Recently, another supramolecular assembly based approach was developed using melamine, phosphoric acid, and GO for the development of HER catalyst with high activity.^[^ ^483^ ^]^ This catalyst demonstrated exceptional stability for 6000 cycles (Figure 19E–G). The XPS data showed a very small change in the N and P content and the hydrogen evolution matched very well with the theoretical prediction. However, a completely bottom‐up approach to synthesize N, P co‐doped graphene was demonstrated by Jiang et al. using urea, glucose, and phosphorus as the source for N, C, and P, respectively.^[^ ^484^ ^]^ The N, P co‐doped graphene sheets demonstrated an overpotential of 213 mV and a Tafel slope between 80 and 100 mV per decade for different pyrolysis temperatures (800–1000 °C), as shown in Figure 19H.

The major highlight of using a completely metal‐free catalyst for HER reaction is the long‐term stability of the graphene electrocatalysts. The N, P doped graphene demonstrated high tolerance to both acidic and alkaline HER activity with negligible change in overpotential even after 1000 cycles of testing. Increasing the P dopant concentration in N‐doped graphene to ≈6% resulted in lowering the overpotential to 397 mV at 10 mA cm^‐2^ current density.^[^ ^485^ ^]^ The order of dopant seemed to influence the HER activity as P, N doped graphene demonstrated better HER activity than N, P doped graphene. The high activity of P, N doped graphene resulted from its high crystallinity as well as a high fraction of pyridinic nitrogen content (66%). Recently, Chen et al. introduced a controlled process for dopant allocation within the graphene structure by systematically doping N and P to achieve a minimum N/P ratio of 2.04 while optimizing the ratio of pyridinic nitrogen to graphitic nitrogen as well as maintaining a high PC/PO bond ratio.^[^ ^486^ ^]^ Based on the controlled variation in doping composition and configuration of pyridinic and graphitic nitrogen, a rule of thumb was derived wherein it was found that the variation of pyridinic N/graphitic N ratio controls the overpotential more coarsely as compared to N/P ratio that can only insert a fine control over the overpotential values. Using this information, this study reported an overpotential of 338 mV and a Tafel slope of about 88 mV per decade. Currently, new strategies for singly N doped^[^ ^487^ ^]^ or N, P co‐doped graphene are exploited with a focus on the development of bi or tri‐functional catalysts (ORR, OER, and HER).^[^ ^488^ ^]^

##### S‐Doped Graphene

S as a doping element has also been extensively used for doping graphene in HER applications, and S as a dopant is considered more promising than nitrogen in improving its conductivity and electrochemical activity. The high activity of S‐doped graphene ascribes to many features of C and S bonds. For example, the similar electronegativity of S and C results in the lack of polarization of the C—S bond. In addition, the C—S bond length is also greater than that of the C—C bond length. Furthermore, S atom is found to be above the plane of graphene, which disturbs the symmetry of graphene and affects its charge density. Moreover, S‐doped graphene has better electronic conductivity due to a high electron density and S‐doping as an electron donor.

In one of the first reports on the use of S‐graphene for HER activity, carbon black was used to modify its surface to avoid the stacking and agglomeration of graphene nanosheets.^[^ ^489^ ^]^ It was found that the modified graphene demonstrated higher HER activity. In addition to modification with carbon black, Ru nanoparticles were also employed to modify the surface of S‐graphene, exhibiting much better HER performance than S‐graphene without modification. Besides the surface functionalization by carbon black or Ru nanoparticles, the relatively low inherent catalytic activity of S‐graphene can be improved by creating topological defects. Argon plasma etching was used to create topological defects that synergistically coupled with the S‐doped sites on graphene.^[^ ^490^ ^]^ The plasma etching created nanopores like topological defects and increased the overall defect density, as depicted by an increase in the *I*_D_/*I*_G_ ratio in the Raman spectra. These defect sites can function as active sites for electrocatalytic HER, leading to a low overpotential of 178 mV at 10 mA cm^‐2^ current density that is still higher than the commercial Pt/C (20% Pt) catalyst. A comparison of higher sulfur doping in graphene without plasma‐induced defects showed that even though the HER activity increased with higher sulfur doping concentration, it was lower than that with both sulfur doping and plasma etching, suggesting that S‐doping and plasma‐induced defects act synergistically to create highly electroactive sites. The chemical nature of sulfur suggests that thiophene‐like sulfur sites are preferred over oxidized sulfur sites for higher HER activity.

Codoping with N has also been used as a strategy for enhancing HER performance of S‐doped graphene. A 3D nanoporous highly defective graphene produced by CVD over a sacrificial nanoporous nickel template was doped with both nitrogen and sulfur at different temperatures using pyridine and thiophene as dopants precursors, respectively.^[^ ^491^ ^]^ A bottom‐up strategy was devised for synthesizing high surface area N, S codoped graphene by utilizing diaminopyridine and ammonium persulfate as the N and S source, respectively. This simple strategy could be used to vary N/S ratios, and the best HER activity was achieved with the N/S ratio of 1/3.^[^ ^492^ ^]^ DFT calculations using only one dopant each was compared with double dopant along with defects to ascertain the mechanism of HER activity. The H* adsorption and desorption energy showed a clear picture of the high HER activity of the N, S codoped graphene. While pure graphene has a high H* adsorption energy, the N or S‐doped graphene show high desorption energy. In contrast, the free energy of adsorption of H* on N, S codoped structure shows a very low adsorption energy of 0.12 eV. The effect is enhanced for a carbon defect site located very close to S atoms in the lattice and within close proximity of graphitic N. Thus, the presence of both the codopants and geometric defects in graphene are essential for high HER activity of N, S–codoped graphene and was recently shown to promote HER in both acidic (overpotential ‐0.31 V in 0.5 m H_2_SO_4_) and alkaline (overpotential ‐0.29 in 1 m KOH) medium.^[^ ^493^ ^]^

##### B‐Doped Graphene

The evolution of hydrogen takes place at the cathode, and thus dopants that promote n‐type conductivity of graphene are preferred for HER electrocatalysis. However, p‐type doping has also been tried and reported to show improvement in HER activity. Due to its electronic configuration with one less valence electron than carbon, doping graphene with B results in converting the graphene to a p‐type semiconductor with holes as the major charge carriers.^[^ ^494^ ^]^ One of the first reports on the use of boron‐doped graphene (B‐graphene) for HER activity was published in 2014 using BH_3_‐THF as a borylating agent.^[^ ^495^ ^]^ In a wet chemical approach for the synthesis of B‐graphene, B was substituted in the carbon ring structure by refluxing defective graphene in the presence of borylating agent. Tafel slope obtained from the Tafel plots depicted values 99 mV dec^‐1^ and 130 mV dec^‐1^ for B‐doped graphene and defective graphene, respectively, suggesting a better HER activity of B doped graphene as compared to undoped and defective graphene. Substitution of B is suggested to increase the number of defects and XPS analysis suggested the presence of C‐B bond to ascertain that the HER activity can be ascribed to substitute boron in the ring. Theoretical calculations suggested that —BH_2_ groups on the surface of graphene can display the opposite character to substitutional doping, which confirms that the substitution of B plays an important role in the HER activity.^[^ ^496^ ^]^

Following the study of N‐graphene and g‐C_3_N_4_ hybrid for HER activity, a systematic theoretical study was conducted to evaluate the effect of different graphene dopants on the HER activity of C_3_N_4_‐doped‐graphene hybrid structures. It was found that despite a different type of doping effect exhibited by dopants (=B, N, O, F, P, and S), all the dopants exert an electron‐withdrawing effect on the g‐C_3_N_4_ and B due to its p‐type doping effect is one of the most promising candidates as a hybrid material. The theoretically predicted HER overpotential of 60 mV at full H* coverage suggested a lucrative potential for exploiting the benefit of this hybrid material.^[^ ^497^ ^]^ An experimental proof was reported by demonstrating HER activity from a hybrid B‐graphene‐C_3_N_4_ hybrid material in 0.5 m H_2_SO_4_.^[^ ^498^ ^]^ A low overpotential of 260 mV at 10 mA cm^‐2^ shows that the performance of B‐graphene‐C_3_N_4_ electrocatalyst is similar to the first reported HER activity of N‐graphene‐C_3_N_4_ catalyst (240 mV).^[^ ^477^ ^]^ The better performance of N‐graphene‐C_3_N_4_ composite could be because of the complete coverage of the H* since the adsorption energy of B based composite is 0.06 eV, which is much lower than that of N based composite of 0.18eV at full coverage. At partial coverage (0.67), B‐graphene exhibited higher negative adsorption energy (‐0.22 eV) as compared to N‐graphene (0.09 eV), suggesting that desorption is easier in nitrogen‐doped graphene based composite.

Given the advantages of co‐doping of both p‐ and n‐type dopants, BCN type structures were formed by B, N co‐doping in graphene.^[^ ^499^ ^]^ For the synthesis of B, N co‐doped graphene, polyethylene glycol (PEG) was used as a source of carbon in a catalyst‐free bottom up protocol by mixing PEG with urea (as a nitrogen source) and boric acid (as a boron source). PEG molecular weight controls the final morphology of the B, N co‐doped structure. Low molecular weight favors the curling up of sheets to form BCN tube‐like structures while high molecular weight PEG favors the formation of nanosheets. It was found that both the sheet and tube‐like structures demonstrated high catalytic activity towards HER and B, N co‐doped nanotubes showed much better performance (overpotential ‐216 mV; Tafel slope ‐92 mV per decade) due to a high specific surface area. The catalytic activity was ascribed to the presence of Stone‐Wales defect in the carbon framework. These defects can act as active sites for the adsorption of protons and keep them on the surface for a sufficiently long duration to enable effective electron transfer and reduction. Nitrogen doping provides sufficient electron density to stabilize and adsorb H^+^ ions, while B‐doped sites provide positive centers that can synergistically influence the electronic states and promote the overall HER activity.

In summary, n‐type doping is better than p‐type doping of graphene/CNTs for electrochemical HER performance. Among the heteroatoms, sulfur doping can inject more electrons into graphene/CNTs, so S‐doped systems perform better than N‐doped or B‐doped electrode systems. By integrating these heteroatoms into carbon materials, for example, S‐N co‐doped graphene/CNTs, can perform even better in electrochemical HER. Therefore, the judicious design of hybrids of doped graphene or CNTs with other 2D/1D/0D nanomaterials is highly critical for HER.

#### 6.2.2 DFT Calculations of Heteroatom‐Doped Graphene for HER

**Figure 20:**
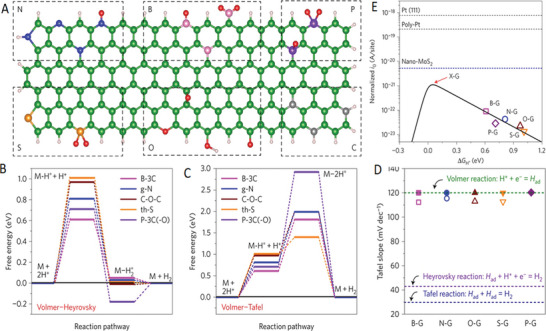
A) Schematic summary of the heteroatom doping configurations: (top row, from left to right) pr‐N, py‐N, g‐N, N—O, B‐2C‐O, B‐3C, B‐C‐2O, P‐3C(‐O) and P‐2C(‐2O); (bottom row, from left to right) th‐S, S‐2O, py‐O, C‐O‐C, C‐OH, C=O, g‐C, z‐C and a‐C. Green/grey, pink, blue, red, gold, purple and white represent C, B, N, O, S, P and H atoms, respectively. B) Free energy diagram for the HER following the Volmer‐Heyrovsky pathway and C) the Volmer‐Tafel pathway on various graphene models. D) Tafel slopes obtained from theoretical computation (filled symbols) and experimental measurement (open symbols) on various graphene models/samples. E) Volcano plot between *i*_0∕site_^theory^ and Δ*G*_H∗_ with charge‐transfer coefficient *α* = 0.125 (black solid line). The open symbols represent *i*_0∕site_^exp^ obtained from Tafel plots and DFT‐derived Δ*G*_H∗_ for each graphene sample/model. Reproduced with permission.^[^ ^500^ ^]^ Copyright 2016, Springer Nature.

Though a majority of studies have been reported on HER activity using doped graphene, a detailed understanding of the origin of such activity following hetero atom doping has puzzled scientists for a long time. Jiao and co‐workers reported a systematic comparison of the theoretical and experimental HER activity of B, N, O, S, and P‐doped graphene to elucidate the mechanism of activity of heteroatom‐doped graphene.^[^ ^500^ ^]^ The atoms strategically replaced carbon atoms at various locations over the graphene, such as the edge or the central carbon atoms, resulting in 15 different combinations of the doping sites (**Figure** **20**A). The results indicated that these doping sites resulted in the creation of 72 different catalytically active sites for HER activity that were compared on the basis of the adsorption energy of hydrogen at the active site. Out of these combinations, the graphitic carbon site in non‐doped graphene showed the lowest activity (highest free energy of adsorption), while the hetero‐atom doping in graphene exhibited lower adsorption energies in the order of B‐ graphene (0.61 eV) < P‐graphene (0.71 eV) < N‐graphene (0.81 eV) < O‐graphene (0.97 eV) < S‐graphene (1.01 eV) for the most optimized configuration of the heteroatom. The mechanism of HER activity was determined using the optimized configuration by comparing the free energy for the formation of intermediate and final products for both the Volmer‐Tafel and Volmer‐Heyrovsky reaction pathways. As observed from Figure 20B–D, the Volmer‐Heyrovsky is the preferred mode of reaction, and the Volmer step is the rate‐controlling step in this reaction. The same work also discussed the activity trend of various doped graphene based on the exchange current density and the resulting volcano plot, as depicted in Figure 20E. It is observed that even the most active single heteroatom doped material B‐graphene shows an exchange current density far below the metal‐based systems. In order to improve the exchange current densities and high overpotential of metal‐free systems, dual heteroatom doping was proposed. It was noted that doping of 10 at% S in N‐graphene with a surface area of 100 m^2^ g^‐1^ could provide comparable HER performance, in terms of exchange current density and the reduced overpotential, to that of MoS_2_.

Following this work, another study was conducted to create B, P, and S co‐doped in N‐graphene using a common doping strategy for effective comparison of the effect of dopant on the HER activity.^[^ ^501^ ^]^ The chemical coupling of polydopamine with B, P, and S precursors such as boric acid, phosphate and thiol functional groups can allow seamless doping of these elements within N‐graphene with a constant N doping concentration. The HER activities in both acidic and alkaline media demonstrated a low overpotential for N, S co‐doped graphene (290 mV—acidic and 380 mV—alkaline) followed by N, P co‐doped graphene (550 mV—acidic and 490 mV—alkaline), while the overpotential value was very high for N, B codoped graphene. This reaffirmed the theoretical predictions made for N, S co‐doped graphene as the most promising co‐doped graphene material for HER activity. As discussed in the S‐doping section, an earlier report on N and S co‐doping in nanoporous graphene showed similar high performance for HER catalysis.

#### 6.2.3 Structural Defects in Graphene

In addition to the doping related defects, structural defects in graphene have also been exploited as a strategy for improving its metal‐free electrocatalytic activity.^[^ ^462^, ^468^, ^502^, ^503^, ^504^ ^]^ The introduction of structural defects such as edges and topological defects (vacancies and deformations) in the graphene structure helps in increasing the catalytic activity by facilitating the anchoring of substances such as hydrogen and oxygen and by acting as a catalytically active site.^[^ ^505^ ^]^ Edge sites provide a large number of dangling bonds that act as high energy centers due to underpassivated coordination, serve as hot spots for adsorption and can exist as random or patterned defects.^[^ ^506^, ^507^, ^508^ ^]^ The high density of functional groups on the edge sites also improves their hydrophilicity, making them suitable for electrolytic water splitting. The activity of dopants is increased manifold on an edge site as compared to a lattice site.^[^ ^509^ ^]^ Specifically for HER, the edge sites have been shown to hugely improve the performance of graphene as compared to those without or with less number of such defects and achieve a low overpotential of 0.15 V at 10 mA cm^‐2^ current density.^[^ ^502^ ^]^ In addition, the defective graphene also shows equivalent performance in alkaline media (1 M KOH) as well in direct contrast to N‐doped graphene which shows sluggish kinetics in alkaline medium. The defects in this study were created by high temperature (1150 °C) treatment to remove the doped nitrogen from N‐doped graphene in a nitrogen environment resulting in the formation of a variety of defects (pentagons, hexagons, and octagons). DFT calculations suggested that the edge sites could not be catalytically active due to high energy of adsorption of hydrogen, while the conjunction carbon possessed lower binding energy and was active in the HER process.

It has been hotly debated whether the structural defects or the dopant‐based defects are better catalytic sites in graphene. Recently, it was demonstrated that indeed the structural defects are better catalytic sites in comparison to doped sites. The active site evaluation showed that the edge pentagon carbon‐based defective patterns exhibited superior catalytic activities to the pyridinic nitrogen sites in highly oriented pyrolytic graphite. (HOPG).^[^ ^510^ ^]^ Similarly, it was reported that increasing the number of edge defect sites using 3D graphene could drastically improve its HER performance.^[^ ^511^ ^]^ 3D graphene synthesized by CVD on a silica nanowire substrate showed 18 mV overpotential at 0.5 mA cm^‐2^. DFT calculations showed that the edge sites with high charge density are catalytically active sites and due to the 3D morphology, a large number of these sites are available for HER. Embedding defects in graphene using various strategies, including bottom up approaches, plasma etching, amidation of carbon nanofibers, integrating porosities through activation and creating 3D structures for improving the HER activity is a hot topic of research and new research is frequently forthcoming in this area.^[^ ^512^, ^513^, ^514^, ^515^, ^516^ ^]^

### 6.3 CNTs‐Based Electrocatalytic HER

Pristine and doped CNTs have been used as electrocatalysts in water splitting for over a decade. As previously mentioned, the first report on the electrocatalytic potential of CNTs was published in 2009, where doped CNTs were used as an electrode material for ORR application. In 2010, Dubey et al. reported the use of CNTs as an electrode material for water electrolysis in alkaline conditions.^[^ ^517^ ^]^ Pellets and a radially oriented configuration of CNTs were adopted for the fabrication of electrodes and tested using a three‐electrode configuration in 1.0 m NaOH. An extremely high rate of hydrogen evolution of 375 lh^−1^ m^−2^ at an anodic voltage of 1.0 V (against SCE) with high exchange current densities was demonstrated. It was suggested that the hydrogen evolution was negligible in acidic pH while the activity was very high in alkaline media due to the adsorption of OH^−^ ions on the defect sites, resulting in the reduction of the activation energy for the dissociation of OH^−^ to O_2_ and consequently lowering the overpotential for H_2_ evolution. Similarly, Wang et al. reported six times higher current density for hydrogen evolution from vertically aligned CNTs compared to the graphite electrodes in acidic electrolytes.^[^ ^518^ ^]^ It was also shown that the stability of the electrode could be improved by using cobalt as a catalyst. This subsection will discuss several common approaches to achieving high‐performance electrocatalytic HER based on CNTs.

#### 6.3.1 Activated CNTs for Electrocatalytic HER

Activation of CNTs to introduce oxygen functionalities can result in better performance in electrocatalysis. Oxygen functionalities can serve as catalytic sites for carrying out multiple electrocatalytic reactions. Cui and co‐workers demonstrated that an activated form of CNTs is a better metal‐free electrocatalyst for HER than non‐activated MWCNTs and glassy carbon electrode (GCE).^[^ ^519^ ^]^ The activation of MWCNTs was performed in nitric acid at 120 °C for different time‐period ranging from 0.5 to 8 h, which resulted in oxidation and insertion of terminal acidic groups. It was also found that the cathodic pre‐treatment at ‐2.0 V of the electrodes made from the activated CNTs improved the HER activity with a high exchange current density of 16 × 10^−3^ mA cm^−2^. The high Tafel slope of 70 mv dec^−1^ suggested that the HER followed a Volmer‐Heyrovsky mechanism and that the electrochemical desorption was the rate‐determining step. A higher number of acidic groups were formed on the MWCNT surface following the acidic oxidation that acted as proton relays, thereby assisting the catalytic process of hydrogen evolution.

At about the same time, another study reported the use of SWCNTs for both HER and hydrogen oxidation reaction (HOR) following an electrochemical activation process in acidic media.^[^ ^520^ ^]^ For activation, a 1.5 µm thick bundle of SWCNTs was soaked in 1 m H_2_SO_4_ solution, and a cyclic scan was run for five times between 0.2 and ‐0.7 V against NHE at a scan rate of 50 mV s^‐1^. The electrode was then aged in the acidic solution for a few hours before repeating the same procedure until saturation of the shift in current density and the onset potential of HER was obtained. Upon completion of the activation process for ≈120–144 h, the onset potential for HER was 0.0 V which corresponds to an incredible zero overpotential mimicking the performance of Pt/GDE (gas diffusion electrodes). The current density for HER, at ‐0.2 V, also showed a remarkably high value of 30 mA cm^‐2^ as compared to 86 mA cm^‐2^ for Pt/GDE that actually corresponded to a specific activity of 278 A g^‐1^ for SWNT as compared to 172 A g^‐1^ for the Pt/GDE. The HER activity was still very high even at neutral pH with an overpotential of only 70 mV after the activation process. It was suggested that the activation process led to the intercalation of the acid in the SWCNT and reduced the *I*_D_/*I*_G_ ratio in the Raman spectra, suggesting that the defects do not contribute to the high activity. The role of sulfuric acid was also ruled out by the demonstration of similar activation in nitric acid solutions. The authors also comprehensively eliminated any possibility of the contribution of Co and Ni in HER activity by thoroughly washing the samples and quantifying the presence of these metals.

Acid oxidation followed by a hydrothermal treatment was shown as another method of improving the HER activity of CNTs.^[^ ^521^ ^]^ This method seems to overcome the limitation of the high‐temperature annealing process to recover the defects induced by the oxidation process, which results in a drastic reduction in the number of surface oxygen functionalities. A mild hydrothermal treatment of MWCNTs followed by the activation in a piranha solution at 100, 140, and 180 °C resulted in retaining a high degree of oxygen functionalities (3.94% O from 4.94% after oxidation in Piranha). Amongst the different temperatures, the hydrothermal treatment at 180 °C showed a current density of 10 mA cm^‐2^ at a potential of ‐0.68 V versus the RHE and an HER onset potential of only 50 mV. As reported previously, the hydrothermal activation could be further improved by the electrochemical oxidation process.^[^ ^519^ ^]^ The cathodic pre‐treatment improves the —OH and C=O content of CNTs which further influences the HER activity.

#### 6.3.2 Functionalization of CNTs for Electrocatalytic HER

**Figure 21:**
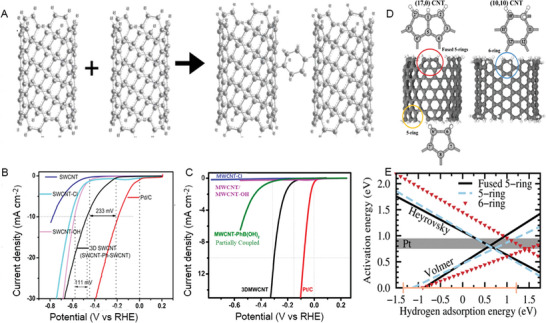
A) Schematic of the Suzuki coupling of CNTs. (B, C) Linear sweep voltamograms (LSVs, in three electrode system, Pt as counter electrode, and Ag/AgCl (sat. KCl) as reference electrode) of different SWCNTs and MWCNTs, respectively. LSVs are performed in 0.5 m H_2_SO_4_ with a scan rate of 10 mV s^‐1^. The onset and over potentials (@ current density 10 mA cm^–2^) are marked. The standard samples such as Pd/C and Pt/C are also compared in the panels. Reproduced with permission.^[^ ^522^ ^]^ Copyright 2017, American Chemical Society. D) Investigated model systems of open‐ended CNTs E) Effect of ring size on HER activation energies on corner sites 3, 7′, and 11′ in the studied open‐ended CNTs. Reproduced with permission.^[^ ^527^ ^]^ Copyright 2015, American Chemical Society.

Covalent functionalization of CNTs has recently experimented as an effective strategy to increase the HER activity of both SWCNTs and MWCNTs. Covalent functionalization can influence the band structure due to a stronger electronic overlap. Pal and co‐workers introduced the covalent functionalization of CNTs to achieve 3D sponge‐like structures of both SWCNTs and MWCNTs that demonstrated high HER activity.^[^ ^522^ ^]^ The covalent bonding was introduced by forming C—C bonds through Suzuki modification (**Figure** **21**A–C) after the removal of the metal impurities by HNO_3_ wash. HER performance of covalently linked SWCNTs is much higher than the functionalized SWCNTs. The HER onset potential and overpotential at 10 mA cm^‐2^ of covalently linked SWCNTs were 233 and 111 mV, respectively, less than that of functionalized SWCNTs. In comparison, the covalently linked 3D sponge of MWCNTs showed a HER onset potential of ‐0.15 V and overpotential of ‐0.3 V (at 10 mA cm^‐2^), while the MWCNTs were inactive towards HER even after acid wash. The high activity of the covalently linked SWCNTs and MWCNTs is ascribed to their band engineering due to the functionalization resulting in a transition from metal to insulator‐like behavior. However, in the presence of H^+^ ions, the band structure is further modified due to the greater proximity of the H^+^ ions after covalent functionalization and a reverse insulator to metal‐like transition is observed, resulting in a charge transfer from covalently linked MWCNTs to the H^+^ ions.

CNT‐graphene hybrid aerogels have been employed in electrochemical HER. Wei's group reported on the synthesis and performance of a novel 3D MoS_2_/Co_9_S_8_/rGO‐CNTs nanocomposite as a high active and stable electrocatalyst for HER.^[^ ^523^ ^]^ The 3D porous structure maximally exposes the active edges of both MoS_2_ and Co_9_S_8_, resulting in a low overpotential (*η*_10_) at about 90 mV (0.5 m H_2_SO_4_), 176 mV (1.0 m PBS), and 102 mV (1.0 m KOH), and high stability of more than 20 h or 1000 cycles under different conditions. The composite aerogels possess high conductivity and exhibit high performance, stability, and pH‐universality for the HER.

#### 6.3.3 Doped CNTs for Electrocatalytic HER

Similar to the use of doped graphene, doped CNTs have been used to improve the HER properties of otherwise inert CNTs.^[^ ^524^, ^525^ ^]^ In one representative work, N‐doped MWCNTs were prepared using an ex‐situ doping method by the adsorption of emeraldine salt over the CNTs with ultrasonic agitation for over 20 h.^[^ ^526^ ^]^ The mixture was then pyrolyzed at 800 °C in an inert Ar atmosphere to synthesize N‐doped MWCNTs. The N‐doped MWCNT was tested for both ORR and HER activity as a bifunctional metal‐free catalyst. The content of pyridinic nitrogen in the hybrid structure increased upon pyrolysis, resulting in a better HER activity shown by the doped CNTs. Another important characteristic is the chemical environment of the dopant within the lattice. It is usually known that the nitrogen atoms inserted within the lattice of carbon structures are strongly associated with high catalytic activity. However, in this study, it was shown that the nitrogen moieties were not present within the CNT lattice but rather as an additional pyrolyzed nitrogen‐containing external structure. Thus, the nitrogen in the outer structure interacts with the electronic states of the MWCNT acting as a catalyst site while the MWCNTs act as catalyst support.

Similarly, another study reported the higher catalytic activity of N‐doped CNTs compared to pristine or acid‐activated CNTs in 0.5 m H_2_SO_4_, supporting the mechanism discussed above,^[^ ^528^ ^]^ while the presence of an external N coped carbon structure over the SWCNTs was shown to improve the HER activity in another study.^[^ ^529^ ^]^ In addition, it was found that combining N‐doped graphene with N‐doped CNTs merged the advantages of the sheet‐like geometry of 2D graphene as well as the curved geometry of 1D CNTs with nitrogen doping.^[^ ^530^ ^]^ This unique structure achieved an onset potential of ‐16 mV with an overpotential of ‐62 mV (at 10 mA cm^‐2^) in acidic solutions. The hybrid structure also demonstrated better properties than the independent N‐doped truncated CNTs and N‐doped graphene. Similar results were shown in alkaline media for a graphene–CNT composite.^[^ ^531^ ^]^ Therefore, it is expected that hybrid and composite materials will be the focus of the research in the next few years to achieve high‐performance metal‐free carbon nanostructures for HER.

#### 6.3.4 Theoretical Calculations of Doped and Defected CNTs for HER

DFT calculations showed that CNTs are not as inert to HER as previously thought.^[^ ^527^ ^]^ Using open ended (17,0) and (10,10) CNTs, it was shown that the terminal open ends could form a 5‐member ring, 5‐member fused ring or 6‐member ring structures (Figure 21D). While the 6‐member ring structure remained comparatively inert, the 5‐member ring and the 5‐member fused rings showed considerable HER activity depending upon the location of the valence orbitals with respect to the location of the highest occupied molecular orbital (HOMO). It was found that for a fixed hydrogen coverage over the open ends of CNTs, sites in the 5‐member ring (and fused ring) structures with the smallest energy difference between the HOMO and the valence band demonstrated the highest activity as compared to sites on the 6‐membered ring structures following a Volmer‐Heyrovsky mechanism as shown in Figure 21E. The experimental validation of such structures is important to correlate with the theoretical findings, though these studies are entirely missing from the reported literature on metal‐free CNTs‐based HER. At about the same time, Laasonen and Holmberg also studied the HER on pristine and N‐doped CNTs in acidic solutions using DFT calculations.^[^ ^532^ ^]^ This study also included a method to correct the calculations for maintaining a constant electrode potential during the reaction. HER mechanism followed the Volmer‐Heyrovsky pathway, similar to the observations suggested in their previous report,^[^ ^527^ ^]^ while the rate‐determining step is the Heyrovsky reaction. This study also demonstrated that both N‐doped CNT and CNT are active electrocatalysts towards HER reaction. However, at saturated monolayer coverage of hydrogen, there is no difference in the activity of N‐doped and pristine MWCNTs which is in direct contrast to the experimental study reported by Davodi et al. and described in the doped CNT section above.^[^ ^526^ ^]^ The same authors then introduced the solvent dynamics to study the hydrogen evolution on the N‐doped CNT at the solid‐liquid interface and found that the introduction of solvent influenced the stability of the transition states and the products through energetics as well as entropic contributions of the solvent reorganization.^[^ ^533^ ^]^ DFT calculations of a few walled CNTs at 0.5 at% N doping show that the C atoms between the N atoms are the most catalytically active sites with the corresponding free energy of adsorption ≈0.23 eV. Co‐doping more than one heteroatom can also improve the HER efficiency due to the higher concentration of defects and codoping of B and N on the CNTs showed lower onset potential and overpotential than the codoped rGO structures.^[^ ^534^ ^]^ These calculations have also shown that a large number of defect states are present near the Fermi level that originates due to the presence of point defects B and N. The carbon atoms adjacent to these defect states function as catalytically active sites. Recently, it was also shown that different curvatures of N‐doped CNTs could also increase the HER activity of the CNTs as increasing the curvature increased the H binding ability of both pure and doped CNTs.^[^ ^535^ ^]^

### 6.4 Fullerene‐Based Electrocatalytic HER

Fullerenes possess unique chemical and physical properties with their spherical cage and hollow interior structure that have been exploited in a wide variety of applications. However, for electrocatalytic HER, fullerene has been selectively hybridized with other functional materials, and only a few works of fullerene‐based composites and isolated theoretical calculation works have been reported so far. As a derivative of fullerene, fullerenol demonstrated promising HER activity due to its better stability and solubility in water and affinity for hydrogen ions, hence it has also attracted interest in HER research.^[^ ^536^ ^]^ For example, fullerenol with C_60_(OH)_8_ composition was deposited from its potassium salt and immobilized on the glassy carbon electrode with the help of Nafion to prevent its loss to the solution during testing while allowing hydrogen ion diffusion. However, the overpotential in 0.5 m H_2_SO_4_ was only 250 mV at 1 mA cm^‐2^ density with a Tafel slope of 78 mV per decade.

Endohedral C_60_ fullerene with encapsulated metal atoms has been screened as an efficient electrocatalyst for HER application.^[^ ^537^ ^]^ By comparing the differential H* adsorption energy for single‐metal atom encapsulated fullerene for more than 20 different transition and alkali metals, it was found that at least 8 different metals (Na, K, Rb, Cs, Sc, Ti, Mn, Fe) showed very low H* adsorption energy. These metal atoms demonstrated differential H* adsorption energy within a range of ‐0.1 to 0.1 eV that is comparable to many metal‐based catalysts like 2D MoS_2_.^[^ ^14^ ^]^ Among the metals encapsulated within the fullerene cage, Rb@C60 is the most ideal doped fullerene structure with differential free energy close to zero. In fact, all the alkali metals demonstrated good HER activity except Li due to its off‐center location within the fullerene structure arising from its small size. While the activity seemed to be exceptionally high, the authors used a rather simplistic model for HER, considering a two‐step reaction of the formation of an intermediate H* following single electron reduction of H^+^ followed by desorption. This is unlike the Volmer Tafel or Vomer Heyrovsky pathway, and any interaction between adsorbed hydrogen with other adsorbed hydrogen or H^+^ ions was not considered.

A recent article also compared the HER activity of endohedrally doped M@C_60_ fullerene using 70 different elements as dopants.^[^ ^538^ ^]^ In this study, the majority of elements were stabilized at the off‐center location within the fullerene ring and thus transferred charges preferentially to the nearest carbon atom. Hydrogen adsorption for HER activity was considered on these carbon sites. As compared to the previous paper, it was found that not only alkali metals but some of the group 16 and transition metals in group 4 also displayed extremely favorable hydrogen adsorption energy. A volcano plot developed by comparing the overpotential with hydrogen adsorption indicated that several atoms in M@C_60_ have binding energies more favorable or close to the platinum. This favorable adsorption energy is ascribed to the charge transfer from the dopant to the nearest carbon that results in the formation of a radical‐like carbon species on the surface. The electron transfer results in a weakening C—C double bond as confirmed by the increased C—C bond length (1.46–1.48 Å) of endohedrally doped fullerene compared to pristine fullerene (1.45 and 1.40 Å). This leaves behind p_z_ orbital for interaction with H s orbitals, facilitating their adsorption. Another theoretical article by Loius and co‐workers revealed the electrocatalytic HER activities of fullerene‐based nanostructured materials. Through DFT calculations, the HER activities of fullerene, calcium‐encapsulated fullerene, nickel‐doped calcium‐encapsulated fullerene, and silver‐decorated nickel‐doped calcium‐encapsulated fullerene were systematically evaluated. The results demonstrated that the Ag‐decorated and Ni‐doped fullerenes had lower energy gaps and increased electro‐catalytic activities compared to the others, with good surface stability and electro‐catalytic properties for HER, which demonstrated the importance of appropriate doping. However, the experimental work proving such observations is not yet realized and therefore highly anticipated.

### 6.5 Summary of Carbon Nanomaterials‐Based Electrocatalytic HER

The current trends in electrocatalysis from noble metal‐free carbon‐based materials are focused on the development of multifunctional catalysts for performing various activities from a single catalyst. These include strategies such as doping double or triple elements, the formation of hybrid nanostructures with other forms of carbon and the addition of surface heterogeneities in the form of surface functionalization or the addition of secondary phases of metals and metal oxides, sulfides and nitrides.^[^ ^482^, ^521^, ^526^, ^539^, ^540^, ^541^, ^542^, ^543^, ^544^, ^545^, ^546^, ^547^, ^548^, ^549^ ^]^ With respect to modifications in carbon structure, the emphasis going forward will continue to be on merging the chemistry and electronic properties of two or more forms of carbon by creating hybrids of graphene, CNTs, or fullerenes with carbon nitride, carbon quantum dots, boron carbon nitride and doped or functionalized variation of the same materials.^[^ ^547^, ^550^, ^551^ ^]^ In addition, functionalization of carbon structures such as graphene will enable the formation of hybrid heterostructures.^[^ ^552^ ^]^ Keeping in mind the practical applications of these structures in the form of electrodes, the structural engineering in carbon is also picking momentum with the fabrication of 3D structures and mesoporous forms of carbon using either sacrificial templates or creating an in‐situ template. The high surface area and interconnected porosity have the potential to increase the number of sites available to carry out the HER and shorten the diffusion length for the hydrogen ions to access the active sites for adsorption and further reaction. With the new theoretical evidence, the use of doped fullerenes may also need some attention at least in the hybrid forms in addition to the isolated reports with inorganic hybrids.

Another interesting development is the synthesis of hybrid carbon nanostructures‐based catalysts that can be used for HER in both acidic and alkaline mediums.^[^ ^553^ ^]^ This also brings us to the challenges of carbon structures in HER reaction, where the long‐term stability of carbon structures is always debated especially at higher potentials. Defects in graphene and CNTs have been used as active sites, whereas, these sites are also the primary route of carbon corrosion and instability. The bulk synthesis of graphene and doped form of graphene will continue to suffer from the variation in performance as evident from the various reported properties in literature and the standardization of synthesis processes at bulk scale and testing the repeatability of synthesis is also time consuming. Unfortunately, such attempts to solve existing challenges may not find traction at high impact factor publications and hence are often overlooked. In addition, detailed theoretical calculations are required to solve the existing challenges and introduce the solvent dynamics as well as the effect of process parameters on the overall HER evolution. For example, CNTs have always been criticized for the presence of metal impurities that have been related to their electrocatalytic properties.^[^ ^554^, ^555^, ^556^, ^557^ ^]^ Despite several attempts to completely eliminate traces of metal impurities, the synergistic effect of such unintentional dopants in the CNT has to be addressed through comprehensive DFT calculations. These challenges also present an opportunity to work further in the quest for the development of highly active, stable and efficient completely metal‐free carbon‐based catalysts.

## 7 Conclusions and Outlook

Energy is one of the essential requirements in modern‐day society, and nanotechnology has made a significant impact in the field of energy storage and conversion. The element carbon has always been the research focus due to its variety of characteristic properties and forms, especially after the discovery of graphene. The various forms of carbon are inherently suitable for many applications, including adsorption, separation, catalysis, gas capture, energy storage, and conversion. This review focuses on three nanostructured forms of carbon, i.e., graphene, CNTs, and fullerenes, which have garnered enormous attention for their applications in electrochemical energy storage and conversion. Energy storage and conversion is an effective strategy to harness renewable energy as well as store and convert it conveniently for future use. The storage or conversion power of the systems such as supercapacitors, batteries, and HER electrocatalysis depend mainly on the electrode/catalyst materials and the process conditions. From the materials perspective, it is desired to have materials that can deliver a high power and energy density, perform with a high consistency over many cycles, and show high stability exposed to chemicals and heat.

The advanced carbon nanostructures based on graphene, CNTs, and fullerenes offer a plentiful of advantages in terms of their physicochemical and structural properties that could be fine‐tuned for their applications in energy storage and conversion. These materials possess unique morphologies in the shape of thin nanosheets, tubular sheets, or balls with sub‐nano to micrometer channels that allow for enhanced movement of the charges. The features such as high surface area, heteroatom defects, high thermal and chemical stability, and tunable electronic properties are a few other facets of these exciting materials. With a load of recent research over the last decades or so, these materials have unambiguously become a platform as high‐performing materials for energy storage and conversion. The structural and porosity tunability of these materials on both the inner and outer surfaces results in exciting new materials with high‐end properties and electrochemical catalytic activity. The construction of hybrids or composites of these materials has allowed for the development of a) new materials which possess the interior surface of the pores decorated with functional groups for enhanced performance, selectivity, and efficiency, b) the modified materials with surface modifications on the outer surface for enhanced electrocatalytic performance.

Although vastly explored to date, challenges still exist with the use of graphene, CNTs, and fullerenes for energy storage and conversion. Future research may be directed to address several aspects of the field.

1) More research is required to explore efficient and scalable synthesis methods to produce carbon‐based nanostructures with different modifications. These methods should maintain the properties of the pristine materials and improve their compatibility with other energy storage components, such as electrodes and electrolytes. Moreover, the effect of the size, shape, and distribution on the energy storage performance should be thoroughly investigated, which can lead to the design of carbon‐based nanostructures with optimized properties for specific energy storage and conversion applications.
2) The stability of carbon‐based devices should be enhanced. Graphene with high surface areas and mechanical strength has to achieve enduring electrochemical storage over a long period before broad commercialization. It will also be helpful to determine the effect of the electrocatalytic activity of such nanostructures on HER. The testing of these nanostructures in both aqueous and non‐aqueous electrolytes is significant. Furthermore, the prevention of the restacking of the graphene layers to retain the complete characteristics of the 2D graphene by using suitable stabilizing agents between the layers could be explored. Future research could also address the factors that could enhance the energy storage and conversion of graphene‐based composites with either polymers or transition metal compounds.
3) CNTs exist in various forms, such as SWCNTs, MWCNTs, and CNT arrays. Each of them exhibits different properties and therefore shows different abilities for energy storage and conversion. Various forms of CNTs could be modified by the introduction of defects, surface functionalization, heteroatom doping, and functionalization of the inner and outer pore surfaces. Hence it becomes significantly essential to direct future research toward controlling the structure vs property vs functionalization relationships for enhanced performance in energy storage and conversion.
4) Most fullerene studies are limited in solar cell fields as efficient electron transport materials,^[^ ^558^, ^559^, ^560^, ^561^ ^]^ but relatively less explored for their energy storage and conversion abilities compared to graphene and CNTs. Therefore, more research uncovering the structural, physical‐chemical and porosity features of fullerenes is crucial for realizing their full potential for energy storage and conversion. The structure‐property relationships for the composites of fullerene with other materials, such as transition metal compounds, need more exploration. Future research could unravel the full potential of fullerenes and their composites for energy storage and conversion.
5) Dimensional mixing by hybridizing three types of carbon nanostructures can further add values and new functionalities. Additionally, they can be hybridized with conventional/novel nanomaterials (e.g., nanodiamonds/graphdiyne) to generate 1D–0D, 2D–0D, 1D–2D–0D hybrids. Carbon‐based hybrid aerogels can also be another promising route for energy applications, in which CNTs and fullerenes can simultaneously be introduced along with graphene during the aerogelling process. For example, graphene–CNT hybrid aerogels have been employed as supercapacitor with optimal performance.^[^ ^562^ ^]^ These advanced materials have high thermal transport behavior.^[^ ^563^ ^]^ Adequate functionalization/activation of these carbon‐based hybrid nanomaterials can lead to enhanced storage and conversion. Opportunities are still open for hybridizing these carbon materials with newly discovered Xenes (e.g., silicene, antomonene, stannene, etc.), 2D oxides, 2D nitrides, 2D phosphides, 2D borides etc. Thus, a) morphology manipulation (e.g., partially unzipping nanotubes), b) surface area maximization via aerogel formation, c) surface functionalization (oxidative/amide/halide/hydrogenation etc.), d) heteroatom doping (N,P,S,B, etc.) or co‐doping (N—S, P—B, etc.), and e) hybridization with various carbon allotropes and various classes of 2D materials and other inorganic nanomaterials systems can be implemented to obtain optimized performances in energy devices.
6) More latest and advanced applications of these carbon nanomaterials have to be explored. With fruitful excellent and unique properties as previously mentioned, these carbon materials should not be only limited in conventional applications like catalysis and energy storage. One typical example could be emerging power generation devices, such as moisture‐enabled‐electric nanogenerators.^[^ ^564^ ^]^ The surface of carbon materials can be flexibly functionalized with versatile hydrophilic groups, thus capturing low‐level moisture and generating electricity. Negative air ions are another hot and eye‐drawing topic related to pollutant removal and human health. For example, it has been reported that graphene‐based composites showed high efficiency of negative ions release.^[^ ^565^ ^]^ Last but not least, in this post‐pandemic remote lifestyle, AI and machine learning are to advance, but the downscaling of the transistor size is gradually reaching the physical limit. In this context, 2D graphene^[^ ^566^ ^]^ and 1D CNTs^[^ ^567^ ^]^ are believed to be next‐generation materials to replace traditional silicon‐based electronics.

Overall, more efforts are required to gain insights into the fundamental mechanisms of energy storage and conversion using graphene, CNTs, and fullerenes, three primary carbon forms, through advanced characterization tools. The future of these advanced carbon‐based nanostructures is bright, and new endeavors in this research field will likely lead to scientific breakthroughs that will enhance their prospects for energy storage and conversion on a larger scale that could pave the way for commercialization.

## Conflict of Interest

The authors declare no conflict of interest.

## Author Contributions

G.K., G.S., and X.G. contributed equally to this work. G.K., G.S., X.G., J.M.L., A.K., K.R., and S.J.—preparation and revision. J.Y. and P.K.—reviewing and editing. A.V.—supervision, reviewing, and editing.
